# Proceedings of the 3rd Biennial Conference of the Society for Implementation Research Collaboration (SIRC) 2015: advancing efficient methodologies through community partnerships and team science: Seattle, WA, USA. 24-26 September 2015

**DOI:** 10.1186/s13012-016-0428-0

**Published:** 2016-06-23

**Authors:** Cara Lewis, Doyanne Darnell, Suzanne Kerns, Maria Monroe-DeVita, Sara J. Landes, Aaron R. Lyon, Cameo Stanick, Shannon Dorsey, Jill Locke, Brigid Marriott, Ajeng Puspitasari, Caitlin Dorsey, Karin Hendricks, Andria Pierson, Phil Fizur, Katherine A. Comtois, Lawrence A. Palinkas, Patricia Chamberlain, Gregory A. Aarons, Amy E. Green, Mark. G. Ehrhart, Elise M. Trott, Cathleen E. Willging, Maria E. Fernandez, Nicholas H. Woolf, Shuting Lily Liang, Natalia I. Heredia, Michelle Kegler, Betsy Risendal, Andrea Dwyer, Vicki Young, Dayna Campbell, Michelle Carvalho, Yvonne Kellar-Guenther, Laura J. Damschroder, Julie C. Lowery, Sarah S. Ono, Kathleen F. Carlson, Erika K. Cottrell, Maya E. O’Neil, Travis L. Lovejoy, Joanna J. Arch, Jill L. Mitchell, Cara C. Lewis, Brigid R. Marriott, Kelli Scott, Jennifer Schurer Coldiron, Eric J. Bruns, Alyssa N. Hook, Benjamin C. Graham, Katelin Jordan, Rochelle F. Hanson, Angela Moreland, Benjamin E. Saunders, Heidi S. Resnick, Shannon Wiltsey Stirman, Cassidy A. Gutner, Jennifer Gamarra, Dawne Vogt, Michael Suvak, Jennifer Schuster Wachen, Katherine Dondanville, Jeffrey S. Yarvis, Jim Mintz, Alan L. Peterson, Elisa V. Borah, Brett T. Litz, Alma Molino, Stacey Young McCaughan, Patricia A. Resick, Nancy Pandhi, Nora Jacobson, Neftali Serrano, Armando Hernandez, Elizabeth Zeidler- Schreiter, Natalie Wietfeldt, Zaher Karp, Michael D. Pullmann, Barbara Lucenko, Bridget Pavelle, Jacqueline A. Uomoto, Andrea Negrete, Molly Cevasco, Suzanne E. U. Kerns, Robert P. Franks, Christopher Bory, Edward J. Miech, Teresa M. Damush, Jason Satterfield, Derek Satre, Maria Wamsley, Patrick Yuan, Patricia O’Sullivan, Helen Best, Susan Velasquez, Miya Barnett, Lauren Brookman-Frazee, Jennifer Regan, Nicole Stadnick, Alison Hamilton, Anna Lau, Jennifer Regan, Alison Hamilton, Nicole Stadnick, Miya Barnett, Anna Lau, Lauren Brookman-Frazee, Nicole Stadnick, Anna Lau, Miya Barnett, Jennifer Regan, Scott Roesch, Lauren Brookman-Frazee, Byron J. Powell, Thomas J. Waltz, Matthew J. Chinman, Laura Damschroder, Jeffrey L. Smith, Monica M. Matthieu, Enola K. Proctor, JoAnn E. Kirchner, Thomas J. Waltz, Byron J. Powell, Matthew J. Chinman, Laura J. Damschroder, Jeffrey L. Smith, Monica J. Matthieu, Enola K. Proctor, JoAnn E. Kirchner, Monica M. Matthieu, Craig S. Rosen, Thomas J. Waltz, Byron J. Powell, Matthew J. Chinman, Laura J. Damschroder, Jeffrey L. Smith, Enola K. Proctor, JoAnn E. Kirchner, Sarah C. Walker, Asia S. Bishop, Mariko Lockhart, Allison L. Rodriguez, Luisa Manfredi, Andrea Nevedal, Joel Rosenthal, Daniel M. Blonigen, Anne M. Mauricio, Thomas D. Dishion, Jenna Rudo-Stern, Justin D. Smith, Jill Locke, Courtney Benjamin Wolk, Colleen Harker, Anne Olsen, Travis Shingledecker, Frances Barg, David Mandell, Rinad S. Beidas, Marissa C. Hansen, Maria P. Aranda, Isabel Torres-Vigil, Bryan Hartzler, Bradley Steinfeld, Tory Gildred, Zandrea Harlin, Fredric Shephard, Matthew S. Ditty, Andrea Doyle, John A. Bickel, Katharine Cristaudo, Dan Fox, Sonia Combs, David H. Lischner, Richard A. Van Dorn, Stephen J. Tueller, Jesse M. Hinde, Georgia T. Karuntzos, Maria Monroe-DeVita, Roselyn Peterson, Doyanne Darnell, Lucy Berliner, Shannon Dorsey, Laura K. Murray, Yevgeny Botanov, Beverly Kikuta, Tianying Chen, Marivi Navarro-Haro, Anthony DuBose, Kathryn E. Korslund, Marsha M. Linehan, Colleen M. Harker, Elizabeth A. Karp, Sarah R. Edmunds, Lisa V. Ibañez, Wendy L. Stone, Jack H. Andrews, Benjamin D. Johnides, Estee M. Hausman, Kristin M. Hawley, Beth Prusaczyk, Alex Ramsey, Ana Baumann, Graham Colditz, Enola K. Proctor, Yevgeny Botanov, Beverly Kikuta, Tianying Chen, Marivi Navarro-Haro, Anthony DuBose, Kathryn E. Korslund, Marsha M. Linehan, Colleen M. Harker, Elizabeth A. Karp, Sarah R. Edmunds, Lisa V. Ibañez, Wendy L. Stone, Mimi Choy-Brown, Jack H. Andrews, Benjamin D. Johnides, Estee M. Hausman, Kristin M. Hawley, Beth Prusaczyk, Alex Ramsey, Ana Baumann, Graham Colditz, Enola K. Proctor, Rosemary D. Meza, Shannon Dorsey, Shannon Wiltsey-Stirman, Georganna Sedlar, Leah Lucid, Caitlin Dorsey, Brigid Marriott, Nelson Zounlome, Cara Lewis, Cassidy A. Gutner, Candice M. Monson, Norman Shields, Marta Mastlej, Meredith SH Landy, Jeanine Lane, Shannon Wiltsey Stirman, Natalie K. Finn, Elisa M. Torres, Mark. G. Ehrhart, Gregory A. Aarons, Carol A. Malte, Aline Lott, Andrew J. Saxon, Meredith Boyd, Kelli Scott, Cara C. Lewis, Jennifer D. Pierce, Agathe Lorthios-Guilledroit, Lucie Richard, Johanne Filiatrault, Kevin Hallgren, Shirley Crotwell, Rosa Muñoz, Becky Gius, Benjamin Ladd, Barbara McCrady, Elizabeth Epstein, John D. Clapp, Danielle E. Ruderman, Melanie Barwick, Raluca Barac, Stanley Zlotkin, Laila Salim, Marnie Davidson, Alicia C. Bunger, Byron J. Powell, Hillary A. Robertson, Christopher Botsko, Sara J. Landes, Brandy N. Smith, Allison L. Rodriguez, Lindsay R. Trent, Monica M. Matthieu, Byron J. Powell, Enola K. Proctor, Melanie S. Harned, Marivi Navarro-Haro, Kathryn E. Korslund, Tianying Chen, Anthony DuBose, André Ivanoff, Marsha M. Linehan, Antonio R. Garcia, Minseop Kim, Lawrence A. Palinkas, Lonnie Snowden, John Landsverk, Annika C. Sweetland, Maria Jose Fernandes, Edilson Santos, Cristiane Duarte, Afrânio Kritski, Noa Krawczyk, Caitlin Nelligan, Milton L. Wainberg, Gregory A. Aarons, David H. Sommerfeld, Benjamin Chi, Echezona Ezeanolue, Rachel Sturke, Lydia Kline, Laura Guay, George Siberry, Ian M. Bennett, Rinad Beidas, Rachel Gold, Johnny Mao, Diane Powers, Mindy Vredevoogd, Jurgen Unutzer, Jennifer Schroeder, Lane Volpe, Julie Steffen, Shannon Dorsey, Michael D Pullmann, Suzanne E. U. Kerns, Nathaniel Jungbluth, Lucy Berliner, Kelly Thompson, Eliza Segell, Pearl McGee-Vincent, Nancy Liu, Robyn Walser, Jennifer Runnals, R. Keith Shaw, Sara J. Landes, Craig Rosen, Janet Schmidt, Patrick Calhoun, Ruth L. Varkovitzky, Sara J. Landes, Amy Drahota, Jonathan I. Martinez, Brigitte Brikho, Rosemary Meza, Aubyn C. Stahmer, Gregory A. Aarons, Anna Williamson, Ronnie M. Rubin, Byron J. Powell, Matthew O. Hurford, Shawna L. Weaver, Rinad S. Beidas, David S. Mandell, Arthur C. Evans, Byron J. Powell, Rinad S. Beidas, Ronnie M. Rubin, Rebecca E. Stewart, Courtney Benjamin Wolk, Samantha L. Matlin, Shawna Weaver, Matthew O. Hurford, Arthur C. Evans, Trevor R. Hadley, David S. Mandell, Donald R. Gerke, Beth Prusaczyk, Ana Baumann, Ericka M. Lewis, Enola K. Proctor, Jenna McWilliam, Jacquie Brown, Michelle Tucker, Kathleen P Conte, Aaron R. Lyon, Meredith Boyd, Abigail Melvin, Cara C. Lewis, Freda Liu, Nathaniel Jungbluth, Amelia Kotte, Kaitlin A. Hill, Albert C. Mah, Priya A. Korathu-Larson, Janelle R. Au, Sonia Izmirian, Scott Keir, Brad J. Nakamura, Charmaine K. Higa-McMillan, Brittany Rhoades Cooper, Angie Funaiole, Eleanor Dizon, Eric J. Hawkins, Carol A. Malte, Hildi J. Hagedorn, Douglas Berger, Anissa Frank, Aline Lott, Carol E. Achtmeyer, Anthony J. Mariano, Andrew J. Saxon, Kate Wolitzky-Taylor, Richard Rawson, Richard Ries, Peter Roy-Byrne, Michelle Craske, Dena Simmons, Catalina Torrente, Lori Nathanson, Grace Carroll, Justin D. Smith, Kimbree Brown, Karina Ramos, Nicole Thornton, Thomas J. Dishion, Elizabeth A. Stormshak, Daniel S. Shaw, Melvin N. Wilson, Mimi Choy-Brown, Emmy Tiderington, Bikki Tran Smith, Deborah K. Padgett, Ronnie M. Rubin, Marilyn L. Ray, Abraham Wandersman, Andrea Lamont, Gordon Hannah, Kassandra A. Alia, Matthew O. Hurford, Arthur C. Evans, Lisa Saldana, Holle Schaper, Mark Campbell, Patricia Chamberlain, Valerie B. Shapiro, B.K. Elizabeth Kim, Jennifer L. Fleming, Paul A. LeBuffe, Sara J. Landes, Cara C. Lewis, Allison L. Rodriguez, Brigid R. Marriott, Katherine Anne Comtois, Cara C. Lewis, Cameo Stanick, Bryan J. Weiner, Heather Halko, Caitlin Dorsey

**Affiliations:** Department of Psychological and Brain Sciences, Indiana University, 1101 E. 10th St., Bloomington, IN 47405 USA; Department of Psychiatry & Behavioral Sciences, University of Washington, 1959 NE Pacific Street, Box 356560, Rm BB1644, Seattle, WA 98195-6560 USA; Division of Public Behavioral Health and Justice Policy, University of Washington, 2815 Eastlake Ave., E. Suite 200, Seattle, WA 98102 USA; National Center for PTSD, VA Palo Alto Health Care System, 795 Willow Road, PTSD-334, Menlo Park, CA 94025 USA; Department of Psychiatry, University of Arkansas for Medical Sciences, 4301 West Markham St., Little Rock, AR 72205 USA; Psychology Department, University of Montana, 32 Campus Drive, Skaggs 143, Missoula, MT 59812 USA; Department of Psychology, University of Washington, Guthrie Hall, Seattle, WA 98195 USA; Department of Psychology, La Salle University, 1900 West Olney Avenue, Philadelphia, PA 19141 USA; Department of Speech and Hearing Sciences, University of Washington, Box 357920, Seattle, WA 98195 USA; Department of Psychological Sciences, University of Missouri, 320 S. 6th St., Columbia, MO 65211 USA; Department of Children Youth and Families, School of Social Work, University of Southern California, Los Angeles, CA 90089 USA; Oregon Social Learning Center, Eugene, OR 97401 USA; Department of Psychiatry, University of California, San Diego, La Jolla, CA 92093 USA; Child and Adolescent Services Research Center (CASRC), San Diego, CA 92123 USA; Department of Psychology, San Diego State University, San Diego, CA 92182 USA; Pacific Institute for Research and Evaluation, Behavioral Health Research Center of the Southwest, Albuquerque, NM 87102 USA; School of Public Health, University of Texas Health Science Center at Houston, Houston, TX 77030 USA; Woolf Consulting, Santa Barbara, CA 93102 USA; Rollins School of Public Health, Emory University, Atlanta, GA 30322 USA; University of Colorado Cancer Center at Denver, Aurora, CO 80045 USA; South Carolina Primary Health Care Association, Columbia, SC 29203 USA; Veterans Affairs (VA) Ann Arbor Center for Clinical Management Research, Ann Arbor, MI 48105 USA; Personalizing Options through Veteran Engagement (PROVE) Quality Enhancement Research Initiative (QUERI) Program, Ann Arbor, MI 48105 USA; Center to Improve Veteran Involvement in Care (CIVIC), Veterns Affairs (VA) Portland Health Care System (VAPORHCS), Portland, OR 97239 USA; Department of Family Medicine, Oregon Health & Science University, Portland, OR 97239 USA; Division of General Internal Medicine, Department of Medicine, Oregon Health & Science University, Portland, OR 97239 USA; Division of Epidemiology, School of Public Health, Oregon Health & Science University, Portland, OR 97239 USA; Department of Psychiatry, Oregon Health & Science University, Portland, OR 97239 USA; Department of Medical Informatics & Clinical Epidemiology, Oregon Health & Science University, Portland, OR 97239 USA; School of Public Health, Oregon Health & Science University, Portland, OR 97239 USA; Department of Psychology and Neuroscience, University of Colorado Boulder, Boulder, CO 80309 USA; Rocky Mountain Cancer Centers-Boulder, Boulder, CO 80303 USA; Department of Psychological and Brain Sciences, Indiana University, Bloomington, IN 47405 USA; Department of Psychology, University of Washington, Seattle, WA 98195 USA; Department of Psychological Sciences, University of Missouri, Columbia, MO 65211 USA; Division of Public Behavioral Health and Justice Policy, University of Washington, Seattle, WA 98102 USA; National Center for Post-Traumatic Stress Disorder (PTSD), Dissemination and Training Division, Veterans Affairs (VA) Palo Alto Health Care System, Menlo Park, CA 94304 USA; Department of Psychiatry and Behavioral Sciences, National Crime Victims Research and Treatment Center, Medical University of South Carolina, Charleston, SC 29412 USA; National Center for Post-Traumatic Stress Disorder (PTSD), Veterans Affairs (VA) Palo Alto Healthcare System and Stanford University, Menlo Park, CA 94024 USA; National Center for PTSD, VA Boston Healthcare System and Boston University, Boston, MA 02130 USA; Department of Psychology, University of California, Los Angeles, Los Angeles, CA 90095 USA; Department of Psychology, Suffolk University, Boston, MA 02108 USA; Department of Psychiatry, The University of Texas Health Science Center at San Antonio, San Antonio, TX 78229 USA; Headquarters, Carl R. Darnall Army Medical Center, Fort Hood, TX 76544 USA; Department of Epidemiology and Biostatistics, The University of Texas Health Science Center at San Antonio, San Antonio, TX 78229 USA; Office of Research and Development, South Texas Veterans Health Care System, San Antonio, TX 78229 USA; School of Social Work, The University of Texas at Austin, Austin, TX 78712 USA; Department of Psychiatry and Behavioral Sciences, Duke University Medical Center, Durham, NC 27701 USA; Department of Family Medicine and Community Health, University of Wisconsin-Madison, Madison, WI 53715 USA; Institute for Clinical and Translational Research and School of Nursing, University of Wisconsin-Madison, Madison, WI 53705 USA; Foundation for Health Leadership & Innovation, Cary, NC 27513 USA; Group Health Cooperative of South Central Wisconsin, Madison, WI 53703 USA; Access Community Health Centers, Madison, WI 53713 USA; Department of Psychiatry and Behavioral Sciences, University of Washington, Seattle, WA 98109 USA; Washington State Department of Social and Health Services Research and Data Analysis Division, Olympia, WA 98504 USA; Judge Baker Children’s Center, Boston, MA 02120 USA; Department of Veterans Affairs (VA) Health Services Research and Development (HSR&D) PRIS-M Quality Enhancement Research Initiative (QUERI), Indianapolis, Indiana 46202 USA; VA HSR&D Center for Health Information and Communication (CHIC), Richard L. Roudebush VA Medical Center, Indianapolis, Indiana 46202 USA; Department of Internal Medicine, Indiana University School of Medicine, Indianapolis, Indiana 46202 USA; Regenstrief Institute, Indianapolis, Indiana 46202 USA; Department of Emergency Medicine, Indiana University School of Medicine, Indianapolis, Indiana 46202 USA; Department of Medicine, University of California, San Francisco, CA USA; Department of Psychiatry, University of California, San Francisco, CA USA; Treatment Implementation Collaborative, LLC, Seattle, WA 98136 USA; Department of State Hospitals, Sacramento, CA 95814 USA; Department of Psychology, University of California, Los Angeles, Los Angeles, CA 90095 USA; Department of Psychiatry, University of California, San Diego, La Jolla, CA 92093 USA; Child and Adolescent Services Research Center, San Diego, CA 92123 USA; Department of Psychiatry and Biobehavioral Sciences, David Geffen School of Medicine, University of California, Los Angeles, Los Angeles, CA 90095 USA; Department of Psychology, University of California, Los Angeles, Los Angeles, CA 90095 USA; Department of Psychiatry and Biobehavioral Sciences, David Geffen School of Medicine, University of California, Los Angeles, CA 90095 USA; Department of Psychiatry, University of California, San Diego, La Jolla, CA 92093 USA; Child and Adolescent Services Research Center, San Diego, CA 92123 USA; Department of Psychiatry, University of California, San Diego, La Jolla, CA 92093 USA; Child and Adolescent Services Research Center, San Diego, CA 92123 USA; Department of Psychology, University of California, Los Angeles, Los Angeles, CA 90095 USA; Department of Psychology, San Diego State University, San Diego, CA 92182 USA; Department of Health Policy and Management, Gillings School of Global Public Health, University of North Carolina at Chapel Hill, Chapel Hill, NC 27599 USA; Department of Psychology, Eastern Michigan University, Ypsilanti, MI 48197 USA; Veterans Affairs (VA) Center for Clinical Management Research, Ann Arbor, MI 48105 USA; RAND, Pittsburgh, PA 15213 USA; VA Quality Enhancement Research Initiative (QUERI) for Personalizing Options through Veteran Engagement (PROVE), Ann Arbor, MI 48105 USA; VA Quality Enhancement Research Initiative (QUERI) for Team-Based Behavioral Health, North Little Rock, AR 72114 USA; Central Arkansas Veterans Healthcare System, North Little Rock, AR 72114 USA; School of Social Work, Saint Louis University, St. Louis, MO 63104 USA; Brown School, Washington University in St. Louis, St. Louis, MO 63130 USA; Department of Psychiatry, University of Arkansas for Medical Sciences, Little Rock, AR 72205 USA; Department of Psychology, Eastern Michigan University, Ypsilanti, MI 48197 USA; Veterans Affairs (VA) A Center for Clinical Management Research, Ann Arbor, MI 48105 USA; Department of Health Policy and Management, University of North Carolina at Chapel Hill, Chapel Hill, NC 27599 USA; RAND, Pittsburgh, PA 15213 USA; VA Quality Enhancement Research Initiative (QUERI) for PeRsonalizing Options through Veteran Engagement (PROVE), Ann Arbor, MI 48105 USA; VA Quality Enhancement Research Initiative (QUERI) for Team-Based Behavioral Health, North Little Rock, AR 72114 USA; Central Arkansas Veterans Healthcare System, North Little Rock, AR 72114 USA; School of Social Work, Saint Louis University, St. Louis, MO 63103 USA; Central Arkansas Health Care System, North Little Rock, AR 72114 USA; Brown School, Washington University in St. Louis, St. Louis, MO 63130 USA; Department of Psychiatry, University of Arkansas for Medical Sciences, Little Rock, AR 72205 USA; School of Social Work, Saint Louis University, St. Louis, MO 63103 USA; Central Arkansas Health Care System, North Little Rock, AR 72114 USA; Dissemination & Training Division National Center for Posttraumatic Stress Disorder (PTSD), Veterans Affairs (VA) Palo Alto Health Care System, Menlo Park, CA 94025 USA; Department of Psychology, Eastern Michigan University, Ypsilanti, MI 48197 USA; VA Center for Clinical Management Research, Ann Arbor, MI 48105 USA; Department of Health Policy and Management, University of North Carolina at Chapel Hill, Chapel Hill, NC 27599 USA; RAND, Pittsburgh, PA 15213 USA; VA Center for Clinical Management Research and VA Quality Enhancement Research Initiative (QUERI) for PeRsonalizing Options through Veteran Engagement (PROVE), Ann Arbor, MI 48105 USA; VA Quality Enhancement Research Initiative (QUERI) for Team-Based Behavioral Health, Central Arkansas Veterans Healthcare System, North Little Rock, AR 72114 USA; Brown School, Washington University in St. Louis, St. Louis, MO 63130 USA; Department of Psychiatry, University of Arkansas for Medical Sciences, Little Rock, AR 72205 USA; Department of Psychiatry and Behavioral Sciences, University of Washington, Seattle, WA 98102 USA; Seattle Youth Violence Prevention Initiative, Department of Education and Early Learning, City of Seattle, Seattle, WA 98104 USA; National Center for Post-traumatic Stress Disorder (PTSD), Veterans Affairs (VA) Palo Alto Health Care System, Menlo Park, CA 94025 USA; Health Services Research & Development (HSR&D) Center for Innovation to Implementation, Department of Veterans Affairs, Palo Alto Health Care System, Menlo Park, CA 94025 USA; Veterans Health Administration (VHA) Veterans Justice Programs, Department of Veterans Affairs, Washington D.C., 20002 USA; Department of Clinical Psychology, Palo Alto University, Palo Alto, CA 94304 USA; Department of Psychology, Resource for Advancing Children’s Health (REACH) Institute, Arizona State University, Tempe, AZ 85287 USA; Department of Psychiatry and Behavioral Sciences, Feinberg School of Medicine, Northwestern University, Chicago, IL 60657 USA; Department of Speech and Hearing Sciences, University of Washington, Seattle, WA 98195 USA; Department of Psychiatry, University of Pennsylvania, Philadelphia, PA 19104 USA; Department of Psychology, University of Washington, Seattle, WA 98195 USA; New York University School of Medicine, New York, NY 10016 USA; School of Social Work, California State University, Long Beach, CA 90802 USA; School of Social Work, University of Southern California, Los Angeles, CA 90089 USA; Graduate College of Social Work, University of Houston, Houston, TX 77004 USA; Alcohol & Drug Abuse Institute, University of Washington, Seattle, WA 98105 USA; Group Health Cooperative, Seattle, WA USA; The Ebright Collaborative, LLC, Wilmington, DE 19802 USA; School of Social Policy and Practice, University of Pennsylvania, Philadelphia, PA 19104 USA; Lutheran Community Services Northwest, SeaTac, WA 98188 USA; Valant Medical Solutions, Seattle, WA 98121 USA; Evidence Treatment Centers of Seattle, Seattle, WA 98101 USA; University of Washington, Seattle, WA USA; RTI International, Durham, NC 27709 USA; Department of Psychiatry & Behavioral Sciences, University of Washington, Seattle, WA 98102 USA; Department of Psychiatry & Behavioral Sciences, Harborview Medical Center, University of Washington, Seattle, WA 98122 USA; Harborview Center for Sexual Assault and Traumatic Stress, University of Washington, Seattle, WA 98122 USA; Department of Psychology, University of Washington, Seattle, WA 98195 USA; Department of Mental Health, Johns Hopkins Bloomberg School of Public Health, Baltimore, MD 21205 USA; Behavioral Research & Therapy Clinics, Department of Psychology, University of Washington, Seattle, WA 98195 USA; Linehan Institute/Behavioral Tech, LLC, Seattle, WA 98195 USA; Department of Psychology, University of Washington, Seattle, WA 98195 USA; Silver School of Social Work, New York University, New York, NY 10003 USA; Department of Psychological Sciences, University of Missouri, Columbia, Missouri 65211 USA; George Warren Brown School of Social Work, Washington University in St. Louis, St. Louis, MO 63130 USA; School of Medicine, Washington University in St. Louis, St. Louis, MO 63130 USA; Institute for Public Health, Washington University in St. Louis, St. Louis, MO 63130 USA; Department of Psychology, University of Washington, Seattle, WA 98195 USA; National Center for PTSD, VA Palo Alto Healthcare System and Stanford University, Menlo Park, CA 94024 USA; Department of Psychiatry and Behavioral Sciences, University of Washington, Seattle, WA 98102 USA; Department of Psychological and Brain Sciences, Indiana University, Bloomington, IN 47933 USA; Department of Psychological Sciences, University of Missouri, Columbia, MO 65211 USA; Department of Counseling and Educational Psychology, Indiana University, Bloomington, IN 47405 USA; Department of Psychiatry, Boston University School of Medicine, Boston, MA USA; National Center for PTSD at VA Boston Healthcare System, Boston, MA USA; Department of Psychology, Ryerson University, Toronto, Canada; Veterans Affairs Canada, Montreal, Canada; National Center for Post-Traumatic Stress Disorder (PTSD), Veterans Affairs (VA) Palo Alto Healthcare System, Palo Alto, CA USA; Department of Psychiatry, Stanford University, Menlo Park, CA 94304 USA; Department of Psychiatry, University of California, San Diego, La Jolla, CA 92093 USA; Child and Adolescent Services Research Center (CASRC), San Diego, CA 92123 USA; Department of Psychology, San Diego State University, San Diego, CA 92182 USA; Center of Excellence in Substance Abuse Treatment and Education, Veterans Affairs Puget Sound Health Care System, Seattle, WA 98108 USA; Department of Psychiatry and Behavioral Sciences, University of Washington, Seattle, WA USA; Department of Psychological and Brain Sciences, Indiana University, Bloomington, IN 47405 USA; Special Education, University of Washington, Seattle, WA 98101 USA; Institut de recherche en santé publique de l’Université de Montréal (IRSPUM), Montréal, Québec H3C 3J7 Canada; Research Centre, Institut universitaire de gériatrie de Montréal, Montréal, Québec H3W 1W5 Canada; Faculty of Nursing, Université de Montréal, Montréal, Québec H3C 3J7 Canada; School of Rehabilitation, Faculty of Medicine, Université de Montréal, Montréal, Québec H3C 3J7 Canada; Department of Psychiatry and Behavioral Sciences, University of Washington, Seattle, WA 98195 USA; Boston Veteran Affairs Health Care System, Boston, MA 02130 USA; Center on Alcoholism, Substance Abuse, and Addictions, University of New Mexico, Albuquerque, NM 87131 USA; Department of Psychology, Washington State University Vancouver, Vancouver, WA 98686 USA; Department of Psychiatry, University of Massachusetts Medical School, Worcester, MA 01655 USA; College of Social Work, The Ohio State University, Columbus, Ohio 43210 USA; Research Institute, Hospital for Sick Children, Toronto, Ontario M5G 1X8 Canada; Child and Youth Mental Health Research Unit, Psychiatry, Hospital for Sick Children, Toronto, Ontario M5G 1X8 Canada; Department of Psychiatry, University of Toronto, Toronto, Ontario M5T 1R8 Canada; Dalla Lana School of Public Health, University of Toronto, Toronto, Ontario M5G 1X8 Canada; SickKids Centre for Global Child Health, Hospital for Sick Children, Toronto, Ontario M5G 1X8 Canada; Department of Nutritional Sciences, University of Toronto, Toronto, Ontario M5G 1X8 Canada; Department of Paediatrics, University of Toronto, Toronto, Ontario M5G 1X8 Canada; Health and Nutrition, Save the Children Canada, Toronto, Ontario M2P 2A8 Canada; Maternal, Newborn and Child Health, CARE Canada, Ottawa, Ontario K2E 7X6 Canada; College of Social Work, Ohio State University, Columbus, Ohio 43210 USA; Department of Health Policy and Management, Gillings School of Global Public Health, University of North Carolina, Chapel Hill, NC 27599 USA; Altarum Institute, Ann Arbor, Michigan 20910 USA; National Center for Post Traumatic Stress Disorder (PTSD), Veterns Affairs (VA) Palo Alto Health Care System, Menlo Park, CA 94025 USA; Department of Psychiatry, University of Arkansas for Medical Sciences, Little Rock, AR 72205 USA; School of Social Work, Saint Louis University, Saint Louis, MO 63103 USA; Mental Health Services, Central Arkansas Health Care System, North Little Rock, AR 72114 USA; Department of Health Policy and Management, Gillings School of Global Public Health, University of North Carolina at Chapel Hill, Chapel Hill, NC 27599 USA; Brown School, Washington University in St. Louis, St. Louis, MO 63130 USA; Department of Psychology, University of Washington, Seattle, WA 98195 USA; Behavioral Tech, LLC, Seattle, WA 98105 USA; School of Social Policy and Practice, University of Pennsylvania, Philadelphia, PA 19104 USA; Department of Social Work, Chinese University of Hong Kong, Hong Kong, China; School of Social Work, University of Southern California, Los Angeles, CA 90089 USA; School of Public Health, University of California-Berkeley, Oakland, CA 94610 USA; School of Social Work, Washington University, St. Louis, MO 63105 USA; Department of Psychiatry, Columbia College of Physicians and Surgeons and New York State Psychiatric Institute, New York, NY 10032 USA; Itaboraí Municipality of Health, Itaboraí, Brazil; Federal University of Rio de Janeiro, Rio de Janeiro, Brazil; Department of Psychiatry, University of California, San Diego, La Jolla, CA 92093 USA; Department of Obstetrics and Gynecology, University of North Carolina at Chapel Hill, Chapel Hill, NC 27599 USA; School of Community Health Science, University of Nevada Las Vegas, Las Vegas, NV 89154 USA; NIH Fogarty International Center, Bethesda, MD 20892 USA; Elizabeth Glaser Pediatric AIDS Foundation, Washington, DC, 20036 USA; National Institute of Health (NIH) National Institute of Child Health and Human Development, Bethesda, MD 20892 USA; Department of Psychiatry and Behavioral Sciences, University of Washington, Seattle, WA 98195 USA; Department of Family Medicine, University of Washington, Seattle, WA 98195 USA; Department of Psychiatry, University of Pennsylvania, Philadelphia, PA 19104 USA; Center for Health Research, Kaiser Permanente, Portland, OR 97227 USA; The Implementation Group, Louisville, CO 80027 USA; Invest in Kids, Denver, CO 80203 USA; Department of Psychology, University of Washington, Seattle, WA 98195 USA; Psychiatry and Behavioral Sciences, University of Washington, Seattle, WA 98102 USA; Harborview Center for Sexual Assault and Traumatic Stress, Harborview, Seattle, WA 98104 USA; National Center for Post-Traumatic Stress Disorder (PTSD), Veterans Affairs (VA) Palo Alto Health Care System, Menlo Park, CA 94025 USA; Department of Psychology, University of California Berkeley, Berkeley, CA 94720 USA; VA Mid-Atlantic Mental Illness Research Educational and Clinical Center (MIRECC), Durham VA Medical Center, Durham, NC 27705 USA; Department of Psychiatry and Behavioral Sciences, Duke University School of Medicine, Durham, NC 27710 USA; Department of Psychiatry, University of Arkansas for Medical Sciences, Little Rock, AR 72205 USA; Department of Psychiatry and Behavioral Sciences, Stanford University School of Medicine, Stanford, CA 94305 USA; Veterans Affairs (VA) Puget Sound Health Care System – American Lake Division, Tacoma, WA 98493 USA; Department of Psychiatry and Behavioral Science, University of Washington School of Medicine, Seattle, WA 98105 USA; National Center for Post-traumatic stress disorder (PTSD), VA Palo Alto Health Care System, Menlo Park, CA 94025 USA; Department of Psychiatry, University of Arkansas for Medical Sciences, Little Rock, AR 72205 USA; Department of Psychology, San Diego State University, San Diego, CA 92182 USA; Child and Adolescent Service Research Center, San Diego, CA 92123 USA; Department of Psychology, California State University Northridge, Northridge, CA 91330 USA; Department of Psychology, University of Washington, Seattle, WA 98195 USA; Department of Psychiatry and Behavioral Sciences, University of California Davis, Medical Investigation of Neurodevelopmental Disorders (MIND) Institute, Davis, CA 95616 USA; Department of Psychiatry, University of California, San Diego, San Diego, CA 92093 USA; The Sax Institute, Sydney, NSW 2000 Australia; Department of Behavioral Health and Intellectual disAbilities Services, Philadelphia, PA 19107 USA; University of North Carolina, Chapel Hill, NC 27599 USA; Community Care Behavioral Health Organization, Pittsburgh, 15222 USA; University of Pennsylvania, Philadelphia, PA 19104 USA; Department of Health Policy and Management, Gillings School of Global Public Health, University of North Carolina at Chapel Hill, Chapel Hill, NC 27599 USA; Center for Mental Health Policy and Services Research, Department of Psychiatry, Perelman School of Medicine, University of Pennsylvania, Philadelphia, PA 19104 USA; Department of Behavioral Health and Intellectual disAbility Services, Philadelphia, PA 19107 USA; Scattergood Foundation, Philadelphia, PA 19102 USA; Community Care Behavioral Health Organization, Philadelphia, PA 19107 USA; Brown School of Social Work, Washington University in St. Louis, St. Louis, MO 63130 USA; Triple P International, Brisbane, Queensland Australia; Queensland University of Technology, Brisbane, Queensland Australia; Menzies Centre for Health Policy, University of Sydney, Sydney, New South Wales 2004 Australia; University of Washington, Seattle, WA 98115 USA; Indiana University, Bloomington, IN 47405 USA; University of Hawaii at Manoa, Honolulu, HI 96822 USA; University of Hawaii at Hilo, Hilo, HI 96720 USA; Child and Adolescent Mental Health Division (CAMHD), Honolulu, HI 96720 USA; Department of Human Development, Washington State University, Pullman, WA 99164 USA; Health Services Research & Development (HSR&D) Seattle Center of Innovation for Veteran- Centered and Value-Driven Care, Seattle, WA USA; Center of Excellence in Substance Abuse Treatment and Education, Veterans Affairs (VA) Puget Sound Health Care System, Seattle, WA USA; Minneapolis VA Health Care System, Minneapolis, VA USA; VA Quality Enhancement Research Initiative for Substance Use Disorders, Palo Alto, CA USA; Department of Psychiatry and Behavioral Sciences, University of Washington, Seattle, WA 98195 USA; Primary and Specialty Medical Care Service, VA Puget Sound Health Care System, Seattle, WA 98108 USA; Veteran Integrated Service Network 20 Pain Medicine and Functional Rehabilitation Center, VA Puget Sound Health Care System, Seattle, WA 98108 USA; Department of Medicine, University of Washington, Seattle, WA 98195 USA; Department of Psychiatry and Biobehavioral Sciences, University of California-Los Angeles, Los Angeles, CA USA; Department of Psychiatry, University of Washington, Seattle, WA USA; Department of Psychology, University of California-Los Angeles, Los Angeles, CA USA; Department of Psychology, Yale University, New Haven, CT 06511 USA; Department of Psychiatry and Behavioral Sciences, Feinberg School of Medicine, Northwestern University, Chicago, IL 60657 USA; Oregon Social Learning Center, Eugene, OR 97401 USA; University Health Services, University of California Berkeley, Berkeley, CA 94720 USA; Oregon Research Institute, Eugene, OR 97403 USA; REACH Institute, Department of Psychology, Arizona State University, Tempe, AZ 85287 USA; Prevention Science Institute, University of Oregon, Eugene, OR 97403 USA; Department of Psychology, University of Pittsburgh, Pittsburgh, PA 15260 USA; Department of Psychology, University of Virginia, Charlottesville, VA 22904 USA; Silver School of Social Work, New York University, New York, NY 10003 USA; School of Social Work, Rutgers University, New Brunswick, NJ 08901 USA; School of Social Service Administration, University of Chicago, Chicago, IL 60637 USA; Department of Behavioral Health and Intellectual disAbilities Services, Philadelphia, PA 19107 USA; Finger Lakes Law and Social Policy Center, Ithaca, NY 14850 USA; University of South Carolina, Columbia, SC 29208 USA; Gordon Hannah Consulting, Pittsburgh, PA 15201 USA; Community Care Behavioral Health Organization, Pittsburgh, 15222 USA; Oregon Social Learning Center, Eugene, Oregon 97401 USA; School of Social Welfare, University of California Berkeley, Berkeley, CA 94618 USA; Devereux Center for Resilient Children, Devereux Foundation, Villanova, PA 19085 USA; National Center for PTSD, VA Palo Alto Health Care System, Menlo Park, CA 94025 USA; Department of Psychiatry, University of Arkansas for Medical Sciences, Little Rock, AR 72205 USA; Department of Psychological and Brain Sciences, Indiana University, Bloomington, IN 47405 USA; Department of Psychiatry and Behavioral Sciences, University of Washington School of Medicine, Seattle, WA 98104 USA; Psychological and Brain Sciences, Indiana University, Bloomington, IN 47405 USA; Department of Psychology, University of Montana, Missoula, MT 59812 USA; University of North Carolina at Chapel Hill, Chapel Hill, NC 27599 USA

## Abstract

Introduction to the 3rd Biennial Conference of the Society for Implementation Research Collaboration: advancing efficient methodologies through team science and community partnerships

Cara Lewis, Doyanne Darnell, Suzanne Kerns, Maria Monroe-DeVita, Sara J. Landes, Aaron R. Lyon, Cameo Stanick, Shannon Dorsey, Jill Locke, Brigid Marriott, Ajeng Puspitasari, Caitlin Dorsey, Karin Hendricks, Andria Pierson, Phil Fizur, Katherine A. Comtois

A1: A behavioral economic perspective on adoption, implementation, and sustainment of evidence-based interventions

Lawrence A. Palinkas

A2: Towards making scale up of evidence-based practices in child welfare systems more efficient and affordable

Patricia Chamberlain

A3: Mixed method examination of strategic leadership for evidence-based practice implementation

Gregory A. Aarons, Amy E. Green, Mark. G. Ehrhart, Elise M. Trott, Cathleen E. Willging

A4: Implementing practice change in Federally Qualified Health Centers: Learning from leaders’ experiences

Maria E. Fernandez, Nicholas H. Woolf, Shuting (Lily) Liang, Natalia I. Heredia, Michelle Kegler, Betsy Risendal, Andrea Dwyer, Vicki Young, Dayna Campbell, Michelle Carvalho, Yvonne Kellar-Guenther

A3: Mixed method examination of strategic leadership for evidence-based practice implementation

Gregory A. Aarons, Amy E. Green, Mark. G. Ehrhart, Elise M. Trott, Cathleen E. Willging

A4: Implementing practice change in Federally Qualified Health Centers: Learning from leaders’ experiences

Maria E. Fernandez, Nicholas H. Woolf, Shuting (Lily) Liang, Natalia I. Heredia, Michelle Kegler, Betsy Risendal, Andrea Dwyer, Vicki Young, Dayna Campbell, Michelle Carvalho, Yvonne Kellar-Guenther

A5: Efficient synthesis: Using qualitative comparative analysis and the Consolidated Framework for Implementation Research across diverse studies

Laura J. Damschroder, Julie C. Lowery

A6: Establishing a veterans engagement group to empower patients and inform Veterans Affairs (VA) health services research

Sarah S. Ono, Kathleen F. Carlson, Erika K. Cottrell, Maya E. O’Neil, Travis L. Lovejoy

A7: Building patient-practitioner partnerships in community oncology settings to implement behavioral interventions for anxious and depressed cancer survivors

Joanna J. Arch, Jill L. Mitchell

A8: Tailoring a Cognitive Behavioral Therapy implementation protocol using mixed methods, conjoint analysis, and implementation teams

Cara C. Lewis, Brigid R. Marriott, Kelli Scott

A9: Wraparound Structured Assessment and Review (WrapSTAR): An efficient, yet comprehensive approach to Wraparound implementation evaluation

Jennifer Schurer Coldiron, Eric J. Bruns, Alyssa N. Hook

A10: Improving the efficiency of standardized patient assessment of clinician fidelity: A comparison of automated actor-based and manual clinician-based ratings

Benjamin C. Graham, Katelin Jordan

A11: Measuring fidelity on the cheap

Rochelle F. Hanson, Angela Moreland, Benjamin E. Saunders, Heidi S. Resnick

A12: Leveraging routine clinical materials to assess fidelity to an evidence-based psychotherapy

Shannon Wiltsey Stirman, Cassidy A. Gutner, Jennifer Gamarra, Dawne Vogt, Michael Suvak, Jennifer Schuster Wachen, Katherine Dondanville, Jeffrey S. Yarvis, Jim Mintz, Alan L. Peterson, Elisa V. Borah, Brett T. Litz, Alma Molino, Stacey Young McCaughanPatricia A. Resick

A13: The video vignette survey: An efficient process for gathering diverse community opinions to inform an intervention

Nancy Pandhi, Nora Jacobson, Neftali Serrano, Armando Hernandez, Elizabeth Zeidler- Schreiter, Natalie Wietfeldt, Zaher Karp

A14: Using integrated administrative data to evaluate implementation of a behavioral health and trauma screening for children and youth in foster care

Michael D. Pullmann, Barbara Lucenko, Bridget Pavelle, Jacqueline A. Uomoto, Andrea Negrete, Molly Cevasco, Suzanne E. U. Kerns

A15: Intermediary organizations as a vehicle to promote efficiency and speed of implementation

Robert P. Franks, Christopher Bory

A16: Applying the Consolidated Framework for Implementation Research constructs directly to qualitative data: The power of implementation science in action

Edward J. Miech, Teresa M. Damush

A17: Efficient and effective scaling-up, screening, brief interventions, and referrals to treatment (SBIRT) training: a snowball implementation model

Jason Satterfield, Derek Satre, Maria Wamsley, Patrick Yuan, Patricia O’Sullivan

A18: Matching models of implementation to system needs and capacities: addressing the human factor

Helen Best, Susan Velasquez

A19: Agency characteristics that facilitate efficient and successful implementation efforts

Miya Barnett, Lauren Brookman-Frazee, Jennifer Regan, Nicole Stadnick, Alison Hamilton, Anna Lau

A20: Rapid assessment process: Application to the Prevention and Early Intervention transformation in Los Angeles County

Jennifer Regan, Alison Hamilton, Nicole Stadnick, Miya Barnett, Anna Lau, Lauren Brookman-Frazee

A21: The development of the Evidence-Based Practice-Concordant Care Assessment: An assessment tool to examine treatment strategies across practices

Nicole Stadnick, Anna Lau, Miya Barnett, Jennifer Regan, Scott Roesch, Lauren Brookman-Frazee

A22: Refining a compilation of discrete implementation strategies and determining their importance and feasibility

Byron J. Powell, Thomas J. Waltz, Matthew J. Chinman, Laura Damschroder, Jeffrey L. Smith, Monica M. Matthieu, Enola K. Proctor, JoAnn E. Kirchner

A23: Structuring complex recommendations: Methods and general findings

Thomas J. Waltz, Byron J. Powell, Matthew J. Chinman, Laura J. Damschroder, Jeffrey L. Smith, Monica J. Matthieu, Enola K. Proctor, JoAnn E. Kirchner

A24: Implementing prolonged exposure for post-traumatic stress disorder in the Department of Veterans Affairs: Expert recommendations from the Expert Recommendations for Implementing Change (ERIC) project

Monica M. Matthieu, Craig S. Rosen, Thomas J. Waltz, Byron J. Powell, Matthew J. Chinman, Laura J. Damschroder, Jeffrey L. Smith, Enola K. Proctor, JoAnn E. Kirchner

A25: When readiness is a luxury: Co-designing a risk assessment and quality assurance process with violence prevention frontline workers in Seattle, WA

Sarah C. Walker, Asia S. Bishop, Mariko Lockhart

A26: Implementation potential of structured recidivism risk assessments with justice- involved veterans: Qualitative perspectives from providers

Allison L. Rodriguez, Luisa Manfredi, Andrea Nevedal, Joel Rosenthal, Daniel M. Blonigen

A27: Developing empirically informed readiness measures for providers and agencies for the Family Check-Up using a mixed methods approach

Anne M. Mauricio, Thomas D. Dishion, Jenna Rudo-Stern, Justin D. Smith

A28: Pebbles, rocks, and boulders: The implementation of a school-based social engagement intervention for children with autism

Jill Locke, Courtney Benjamin Wolk, Colleen Harker, Anne Olsen, Travis Shingledecker, Frances Barg, David Mandell, Rinad S. Beidas

A29: Problem Solving Teletherapy (PST.Net): A stakeholder analysis examining the feasibility and acceptability of teletherapy in community based aging services

Marissa C. Hansen, Maria P. Aranda, Isabel Torres-Vigil

A30: A case of collaborative intervention design eventuating in behavior therapy sustainment and diffusion

Bryan Hartzler

A31: Implementation of suicide risk prevention in an integrated delivery system: Mental health specialty services

Bradley Steinfeld, Tory Gildred, Zandrea Harlin, Fredric Shephard

A32: Implementation team, checklist, evaluation, and feedback (ICED): A step-by-step approach to Dialectical Behavior Therapy program implementation

Matthew S. Ditty, Andrea Doyle, John A. Bickel III, Katharine Cristaudo

A33: The challenges in implementing muliple evidence-based practices in a community mental health setting

Dan Fox, Sonia Combs

A34: Using electronic health record technology to promote and support evidence-based practice assessment and treatment intervention

David H. Lischner

A35: Are existing frameworks adequate for measuring implementation outcomes? Results from a new simulation methodology

Richard A. Van Dorn, Stephen J. Tueller, Jesse M. Hinde, Georgia T. Karuntzos

A36: Taking global local: Evaluating training of Washington State clinicians in a modularized cogntive behavioral therapy approach designed for low-resource settings

Maria Monroe-DeVita, Roselyn Peterson, Doyanne Darnell, Lucy Berliner, Shannon Dorsey, Laura K. Murray

A37: Attitudes toward evidence-based practices across therapeutic orientations

Yevgeny Botanov, Beverly Kikuta, Tianying Chen, Marivi Navarro-Haro, Anthony DuBose, Kathryn E. Korslund, Marsha M. Linehan

A38: Predicting the use of an evidence-based intervention for autism in birth-to-three programs

Colleen M. Harker, Elizabeth A. Karp, Sarah R. Edmunds, Lisa V. Ibañez, Wendy L. Stone

A39: Supervision practices and improved fidelity across evidence-based practices: A literature review

Mimi Choy-Brown

A40: Beyond symptom tracking: clinician perceptions of a hybrid measurement feedback system for monitoring treatment fidelity and client progress

Jack H. Andrews, Benjamin D. Johnides, Estee M. Hausman, Kristin M. Hawley

A41: A guideline decision support tool: From creation to implementation

Beth Prusaczyk, Alex Ramsey, Ana Baumann, Graham Colditz, Enola K. Proctor

A42: Dabblers, bedazzlers, or total makeovers: Clinician modification of a common elements cognitive behavioral therapy approach

Rosemary D. Meza, Shannon Dorsey, Shannon Wiltsey-Stirman, Georganna Sedlar, Leah Lucid

A43: Characterization of context and its role in implementation: The impact of structure, infrastructure, and metastructure

Caitlin Dorsey, Brigid Marriott, Nelson Zounlome, Cara Lewis

A44: Effects of consultation method on implementation of cognitive processing therapy for post-traumatic stress disorder

Cassidy A. Gutner, Candice M. Monson, Norman Shields, Marta Mastlej, Meredith SH Landy, Jeanine Lane, Shannon Wiltsey Stirman

A45: Cross-validation of the Implementation Leadership Scale factor structure in child welfare service organizations

Natalie K. Finn, Elisa M. Torres, Mark. G. Ehrhart, Gregory A. Aarons

A46: Sustainability of integrated smoking cessation care in Veterans Affairs posttraumatic stress disorder clinics: A qualitative analysis of focus group data from learning collaborative participants

Carol A. Malte, Aline Lott, Andrew J. Saxon

A47: Key characteristics of effective mental health trainers: The creation of the Measure of Effective Attributes of Trainers (MEAT)

Meredith Boyd, Kelli Scott, Cara C. Lewis

A48: Coaching to improve teacher implementation of evidence-based practices (EBPs)

Jennifer D. Pierce

A49: Factors influencing the implementation of peer-led health promotion programs targeting seniors: A literature review

Agathe Lorthios-Guilledroit, Lucie Richard, Johanne Filiatrault

A50: Developing treatment fidelity rating systems for psychotherapy research: Recommendations and lessons learned

Kevin Hallgren, Shirley Crotwell, Rosa Muñoz, Becky Gius, Benjamin Ladd, Barbara McCrady, Elizabeth Epstein

A51: Rapid translation of alcohol prevention science

John D. Clapp, Danielle E. Ruderman

A52: Factors implicated in successful implementation: evidence to inform improved implementation from high and low-income countries

Melanie Barwick, Raluca Barac, Stanley Zlotkin, Laila Salim, Marnie

Davidson

A53: Tracking implementation strategies prospectively: A practical approach

Alicia C. Bunger, Byron J. Powell, Hillary A. Robertson

A54: Trained but not implementing: the need for effective implementation planning tools

Christopher Botsko

A55: Evidence, context, and facilitation variables related to implementation of Dialectical Behavior Therapy: Qualitative results from a mixed methods inquiry in the Department of Veterans Affairs

Sara J. Landes, Brandy N. Smith, Allison L. Rodriguez, Lindsay R. Trent, Monica M. Matthieu

A56: Learning from implementation as usual in children’s mental health

Byron J. Powell, Enola K. Proctor

A57: Rates and predictors of implementation after Dialectical Behavior Therapy Intensive Training

Melanie S. Harned, Marivi Navarro-Haro, Kathryn E. Korslund, Tianying Chen, Anthony DuBose, André Ivanoff, Marsha M. Linehan

A58: Socio-contextual determinants of research evidence use in public-youth systems of care

Antonio R. Garcia, Minseop Kim, Lawrence A. Palinkas, Lonnie Snowden, John Landsverk

A59: Community resource mapping to integrate evidence-based depression treatment in primary care in Brazil: A pilot project

Annika C. Sweetland, Maria Jose Fernandes, Edilson Santos, Cristiane Duarte, Afrânio Kritski, Noa Krawczyk, Caitlin Nelligan, Milton L. Wainberg

A60: The use of concept mapping to efficiently identify determinants of implementation in the National Institute of Health--President’s Emergent Plan for AIDS Relief Prevention of Mother to Child HIV Transmission Implementation Science Alliance

Gregory A. Aarons, David H. Sommerfeld, Benjamin Chi, Echezona Ezeanolue, Rachel Sturke, Lydia Kline, Laura Guay, George Siberry

A61: Longitudinal remote consultation for implementing collaborative care for depression

Ian M. Bennett, Rinad Beidas, Rachel Gold, Johnny Mao, Diane Powers, Mindy Vredevoogd, Jurgen Unutzer

A62: Integrating a peer coach model to support program implementation and ensure long- term sustainability of the Incredible Years in community-based settings

Jennifer Schroeder, Lane Volpe, Julie Steffen

A63: Efficient sustainability: Existing community based supervisors as evidence-based treatment supports

Shannon Dorsey, Michael D Pullmann, Suzanne E. U. Kerns, Nathaniel Jungbluth, Lucy Berliner, Kelly Thompson, Eliza Segell

A64: Establishment of a national practice-based implementation network to accelerate adoption of evidence-based and best practices

Pearl McGee-Vincent, Nancy Liu, Robyn Walser, Jennifer Runnals, R. Keith Shaw, Sara J. Landes, Craig Rosen, Janet Schmidt, Patrick Calhoun

A65: Facilitation as a mechanism of implementation in a practice-based implementation network: Improving care in a Department of Veterans Affairs post-traumatic stress disorder outpatient clinic

Ruth L. Varkovitzky, Sara J. Landes

A66: The ACT SMART Toolkit: An implementation strategy for community-based organizations providing services to children with autism spectrum disorder

Amy Drahota, Jonathan I. Martinez, Brigitte Brikho, Rosemary Meza, Aubyn C. Stahmer, Gregory A. Aarons

A67: Supporting Policy In Health with Research: An intervention trial (SPIRIT) - protocol and early findings

Anna Williamson

A68: From evidence based practice initiatives to infrastructure: Lessons learned from a public behavioral health system’s efforts to promote evidence based practices

Ronnie M. Rubin, Byron J. Powell, Matthew O. Hurford, Shawna L. Weaver, Rinad S. Beidas, David S. Mandell, Arthur C. Evans

A69: Applying the policy ecology model to Philadelphia’s behavioral health transformation efforts

Byron J. Powell, Rinad S. Beidas, Ronnie M. Rubin, Rebecca E. Stewart, Courtney Benjamin Wolk, Samantha L. Matlin, Shawna Weaver, Matthew O. Hurford, Arthur C. Evans, Trevor R. Hadley, David S. Mandell

A70: A model for providing methodological expertise to advance dissemination and implementation of health discoveries in Clinical and Translational Science Award institutions

Donald R. Gerke, Beth Prusaczyk, Ana Baumann, Ericka M. Lewis, Enola K. Proctor

A71: Establishing a research agenda for the Triple P Implementation Framework

Jenna McWilliam, Jacquie Brown, Michelle Tucker

A72: Cheap and fast, but what is “best?”: Examining implementation outcomes across sites in a state-wide scaled-up evidence-based walking program, Walk With Ease

Kathleen P Conte

A73: Measurement feedback systems in mental health: Initial review of capabilities and characteristics

Aaron R. Lyon, Meredith Boyd, Abigail Melvin, Cara C. Lewis, Freda Liu, Nathaniel Jungbluth

A74: A qualitative investigation of case managers’ attitudes toward implementation of a measurement feedback system in a public mental health system for youth

Amelia Kotte, Kaitlin A. Hill, Albert C. Mah, Priya A. Korathu-Larson, Janelle R. Au, Sonia Izmirian, Scott Keir, Brad J. Nakamura, Charmaine K. Higa-McMillan

A75: Multiple pathways to sustainability: Using Qualitative Comparative Analysis to uncover the necessary and sufficient conditions for successful community-based implementation

Brittany Rhoades Cooper, Angie Funaiole, Eleanor Dizon

A76: Prescribers’ perspectives on opioids and benzodiazepines and medication alerts to reduce co-prescribing of these medications

Eric J. Hawkins, Carol A. Malte, Hildi J. Hagedorn, Douglas Berger, Anissa Frank, Aline Lott, Carol E. Achtmeyer, Anthony J. Mariano, Andrew J. Saxon

A77: Adaptation of Coordinated Anxiety Learning and Management for comorbid anxiety and substance use disorders: Delivery of evidence-based treatment for anxiety in addictions treatment centers

Kate Wolitzky-Taylor, Richard Rawson, Richard Ries, Peter Roy-Byrne, Michelle Craske

A78: Opportunities and challenges of measuring program implementation with online surveys

Dena Simmons, Catalina Torrente, Lori Nathanson, Grace Carroll

A79: Observational assessment of fidelity to a family-centered prevention program: Effectiveness and efficiency

Justin D. Smith, Kimbree Brown, Karina Ramos, Nicole Thornton, Thomas J. Dishion, Elizabeth A. Stormshak, Daniel S. Shaw, Melvin N. Wilson

A80: Strategies and challenges in housing first fidelity: A multistate qualitative analysis

Mimi Choy-Brown, Emmy Tiderington, Bikki Tran Smith, Deborah K. Padgett

A81: Procurement and contracting as an implementation strategy: Getting To Outcomes® contracting

Ronnie M. Rubin, Marilyn L. Ray, Abraham Wandersman, Andrea Lamont, Gordon Hannah, Kassandra A. Alia, Matthew O. Hurford, Arthur C. Evans

A82: Web-based feedback to aid successful implementation: The interactive Stages of Implementation Completion (SIC)^TM^ tool

Lisa Saldana, Holle Schaper, Mark Campbell, Patricia Chamberlain

A83: Efficient methodologies for monitoring fidelity in routine implementation: Lessons from the Allentown Social Emotional Learning Initiative

Valerie B. Shapiro, B.K. Elizabeth Kim, Jennifer L. Fleming, Paul A. LeBuffe

A84: The Society for Implementation Research Collaboration (SIRC) implementation development workshop: Results from a new methodology for enhancing implementation science proposals

Sara J. Landes, Cara C. Lewis, Allison L. Rodriguez, Brigid R. Marriott, Katherine Anne Comtois

A85: An update on the Society for Implementation Research Collaboration (SIRC) Instrument Review Project

## Introduction to the 3rd Biennial Conference of the Society for Implementation Research Collaboration: advancing efficient methodologies through team science and community partnerships

### Cara Lewis^1,2^ (clewis11@uw.edu), Doyanne Darnell^2^ (darnelld@uw.edu), Suzanne Kerns^2,3^ (sekerns@uw.edu), Maria Monroe-DeVita^2,3^ (mmdv@uw.edu), Sara J. Landes^2,4,5^ (sjlandes@uams.edu), Aaron R. Lyon^2^ (lyona@uw.edu), Cameo Stanick^6^ (cameo.stanick@umontana.edu), Shannon Dorsey^7^ (dorsey2@uw.edu), Jill Locke^9^ (jjlocke@uw.edu), Brigid R. Marriott^10^ (bmvv5@mail.missouri.edu), Ajeng Puspitasari^1^ (apuspita@indiana.edu), Caitlin N. Dorsey^1^ (cadorsey@indiana.edu), Karin Hendricks^2^ (keh8@uw.edu), Andria Pierson^2^ (apierson@uw.edu), Phil Fizur^8^ (fizurp1@student.lasalle.edu), Katherine A. Comtois^2^ (comtois@uw.edu)

#### ^1^Department of Psychological and Brain Sciences, Indiana University, 1101 E. 10th St., Bloomington, IN 47405, USA; ^2^Department of Psychiatry & Behavioral Sciences, University of Washington, 1959 NE Pacific Street, Box 356560, Rm BB1644 Seattle, WA 98195-6560, USA; ^3^Division of Public Behavioral Health and Justice Policy, University of Washington, 2815 Eastlake Ave., E. Suite 200, Seattle, WA 98102, USA; ^4^National Center for PTSD, VA Palo Alto Health Care System, 795 Willow Road, PTSD-334 Menlo Park, CA 94025, USA; ^5^ Department of Psychiatry, University of Arkansas for Medical Sciences, 4301 West Markham St. Little Rock, AR 72205, USA; ^6^Psychology Department, University of Montana, 32 Campus Drive, Skaggs 143, Missoula, MT 59812, USA; ^7^Department of Psychology University of Washington, Guthrie Hall, Seattle, WA 98195, USA; ^8^La Salle University Department of Psychology 1900 West Olney Avenue Philadelphia, PA 19141, USA; ^9^Department of Speech and Hearing Sciences, University of Washington, Box 357920, Seattle, WA 98195, USA; ^10^Department of Psychological Sciences, University of Missouri, 320 S. 6^th^ St. Columbia, MO 65211, USA

##### **Correspondence:** Cara C. Lewis (clewis11@uw.edu) – ^2^Department of Psychiatry & Behavioral Sciences, University of Washington, 1959 NE Pacific Street, Box 356560, Rm BB1644 Seattle, WA 98195-6560, USA

It is well documented that the majority of adults, children and families in need of evidence-based behavioral health interventions^i^ do not receive them [1, 2] and that few robust empirically supported methods for implementing evidence-based practices (EBPs) exist. The Society for Implementation Research Collaboration (SIRC) represents a burgeoning effort to advance the innovation and rigor of implementation research and is uniquely focused on bringing together researchers and stakeholders committed to evaluating the implementation of complex evidence-based behavioral health interventions. Through its diverse activities and membership, SIRC aims to foster the promise of implementation research to better serve the behavioral health needs of the population by identifying rigorous, relevant, and efficient strategies that successfully transfer scientific evidence to clinical knowledge for use in real world settings [3].

SIRC began as a National Institute of Mental Health (NIMH)-funded conference series in 2010 (previously titled the “Seattle Implementation Research Conference”; $150,000 USD for 3 conferences in 2011, 2013, and 2015) with the recognition that there were multiple researchers and stakeholders^i^ working in parallel on innovative implementation science projects in behavioral health, but that formal channels for communicating and collaborating with one another were relatively unavailable. There was a significant need for a forum within which implementation researchers and stakeholders could learn from one another, refine approaches to science and practice, and develop an implementation research agenda using common measures, methods, and research principles to improve both the frequency and quality with which behavioral health treatment implementation is evaluated. SIRC’s membership growth is a testament to this identified need with more than 1000 members from 2011 to the present.^ii^

SIRC’s primary objectives are to: (1) foster communication and collaboration across diverse groups, including implementation researchers, intermediaries^i^, as well as community stakeholders (SIRC uses the term “EBP champions” for these groups) – and to do so across multiple career levels (e.g., students, early career faculty, established investigators); and (2) enhance and disseminate rigorous measures and methodologies for implementing EBPs and evaluating EBP implementation efforts. These objectives are well aligned with Glasgow and colleagues’ [4] five core tenets deemed critical for advancing implementation science: collaboration, efficiency and speed, rigor and relevance, improved capacity, and cumulative knowledge. SIRC advances these objectives and tenets through in-person conferences, which bring together multidisciplinary implementation researchers and those implementing evidence-based behavioral health interventions in the community to share their work and create professional connections and collaborations.

**Conference Theme**

The overarching goals of this conference series are to foster collaboration and to advance behavioral health implementation evaluation and methodological development. To this end, the Society for Implementation Research Collaboration (SIRC) leadership encourages the majority of attendees to present their work with at least 50 % of attendees serving as first-authors of presentations. The first two conference themes were 1) “Key Issues in Evidence-Based Practice (EBP) Implementation Methods and Research” and 2) “Solving Implementation Research Dilemmas.” This supplement summarizes and describes the main activities and outcomes of the third conference, held in September 2015, with the theme “Advancing Efficient Methodologies through Team Science^i^ and Community Partnerships^i^.” Specifically, the 2015 conference highlighted work by SIRC members who leveraged team science and community partnerships to advance efficient methodologies. The presentation slides and video recordings are available on the SIRC website.

**Pre-Conference Activities**

The first half of the pre-conference was devoted to a series of Implementation Development Workshops (IDW). IDWs are dedicated to members invited to be part of the Society for Implementation Research Collaboration (SIRC)’s Network of Expertise (NoE). Members of the NoE include students (*N =* 38), new investigators (*N =* 36), established investigators (*N =* 108), evidence-based practice (EBP) champions (*N =* 13*)*, and intermediaries (*N =* 11). In line with SIRC objectives, the IDW provides a platform for leveraging the collective wisdom of our NoE in the planning stages of a project to identify creative solutions to enhance the rigor and relevance of methods and measurement used in project evaluation and to increase competitiveness for external funding [5]. IDWs are highly structured and guided by the Behavioral Research in Diabetes Group Exchange Model developed by the PsychoSocial Aspects of Diabetes Study Group [6]. Specifically, new and established investigators and EBP champions provide brief presentations on their developing projects or proposals using a “zero technology” format--only a single page handout summarizing their work and highlighting three target questions to guide the discussion is allowed. Following the presentation, a facilitated discussion ensues to provide presenters with high value and focused feedback to inform and shape works in progress. At the 2015 conference, three rooms were run simultaneously, accommodating 12 unique project/proposal discussions.^iii^

Following the IDWs, SIRC hosted four concurrent workshop sessions, open to all conference attendees. Workshops were consistent with SIRC’s emphasis on research-practice partnerships and advancing implementation science methodology and measurement. Two workshops paneled by EBP champions and implementation researchers provided a forum that aligned with the tenet of improving capacity, defined as training future implementation researchers, and sharing advances made through implementation science with stakeholders [7]: (1) *Sustainability* – *Making EBPs Work in the Long Run*; and (2) *Practical, Empirically Based Resources for Integrating Routine Outcome Monitoring into Clinical Practice*. A third workshop highlighted an innovative, efficient research design and methodology: *Getting SMART*^*iv*^*about Adaptive Implementation Intervention*. Also consistent with the spirit of developing efficient methodologies, the final workshop provided an overview of innovative technologies used in healthcare and suggestions for how to incorporate these technologies into implementation research: *Transformative Healthcare Technologies: What Implementation Researchers and Practitioners Need to know about mHealth, Electronic Health Records (EHR), and Big Data Analytics*.

**Conference Activities**

The main conference began with two plenary presentations. The first discussed a behavioral economic perspective on adoption, implementation, and sustainment of evidence-based practices (EBPs) [8]. The second focused on efficient and affordable scale up of EBPs in child welfare systems [9]. In addition, the conference included four plenary symposia that featured international leaders in implementation science and cutting edge topics aligned with the conference theme of advancing efficient methodologies. The first symposium addressed strategic leadership on EBP implementation [10-12]; the second on common elements to support scale-up and sustainment [13, 14; L. Murray, unpublished observation, March 9, 2016]; the third on data-driven, theoretically-informed processes for efficient and effective international implementation [15; R. Mildon & A. Shlonsky, unpublished observation, March 8, 2016; D. Flynn, unpublished observation, March 8, 2016]; and the fourth on innovative implementation methodologies such as procurement and contracting, automated feedback, and web-based tools [16, 17; D. Atkins, unpublished observation, March 8, 2016].

The conference also held six concurrent breakout sessions with five symposia occurring simultaneously, each with three to four presentations from a variety of speakers including EBP champions, policymakers, and investigators (student, new, and established). The breakout sessions appeared to address three content areas centered on advancing efficient methodologies: key efficiency issues, available and affordable tools, and research design and statistical solutions (Table 1).

**Table 1:** General content areas of breakout session presentations

Key Efficiency Issues	Using Available and Affordable Tools	Research Design and Statistical Solutions
Fidelity and supervision for multiple EBPs in one setting	Use of electronic health records, administrative data, progress monitoring, or other existing program resources to promote evaluation	Adaptive and SMART designs
Models and methods for reducing staff turnover	Continuous quality improvement or examples of iterative small scale trials of change	Analysis of qualitative data
Training efficiency	Use decision support tools	Hybrid designs
Feasible models and methods for long-term implementation and sustainment	Use of computer-based/online implementation methodologies	Methods that can rapidly inform health care practice
Community-led evaluations	Technological solutions	
Matching models of implementation to system needs and capacities		

**Activities to Advance Students and New Investigators**

The 2015 Society for Implementation Research Collaboration (SIRC) conference provided structured opportunities to connect students (undergraduate, post-baccalaureate, graduate students learning implementation science) as well as new investigators (researchers early in their career and/or new to implementation research who have not yet served as Principal Investigator on a large implementation science grant) with established members of our Network of Expertise (NoE). On the first day, SIRC hosted a lunch that matched students and new investigators with more senior members of SIRC including established implementation investigators, intermediaries, and evidence-based practice (EBP) champions. Lunch groups were structured such that three students and new investigators met with one established investigator to facilitate discussion of multiple topics.

Additionally, the conference held a poster presentation reception that accommodated 28 posters. Although open to all levels for presentation of work, the poster presentations often highlight the work of student and new investigators, allowing a more intimate setting to garner advice and feedback on their projects from established investigators and EBP champions. To further elevate the work of student and new investigators, at the 2015 conference, SIRC recognized one student (Rosemary Meza [18]) and two new investigators (Leopoldo J. Cabassa [19]; and Justin D. Smith [20]) for conducting high impact implementation science.

**Attendees**

In its first two years of conferences, the Society for Implementation Research Collaboration (SIRC) consistently received feedback that its intimate size is a strength. Accordingly, the 2015 conference registration was capped at 230 individuals as dictated by the space selection and 227 individuals attended with demographic data obtained through a survey administered as part of our registration process; data was missing from only three attendees.

Conference attendees ranged from very junior (e.g., undergraduate students) to expert and senior-level colleagues (e.g., full professors), with the majority of attendees having obtained a Ph.D. (*N* = 143, 63.0 %). Attendees predominantly identified as implementation researchers (*N* = 140, 62.5 %) and/or evidence-based intervention effectiveness researchers (*N* = 69, 30.4 %).

Although those primarily housed in academia comprised the majority of attendees (e.g., assistant professors [*N* = 28, 12.3 %], professors [N = 24, 10.6 %], doctoral graduate students [N = 24, 10.6 %]), the conference included a variety of stakeholders including evidence-based practice (EBP) champions (*N* = 38, 16.7 %) such as clinicians and agency leaders, and intermediaries (*N* = 4, 1.8 %) such as trainers and implementation practitioners.

Attendees had experience with implementing EBPs across diverse settings including, but not limited to, community mental health centers (*N* = 90, 39.6 %), specialty mental health clinic/outpatient mental health/private practice (*N* = 70, 30.8 %), schools (*N* = 44, 19.4 %), and the U.S. Department of Veterans Affairs (VA; *N* = 40, 17.6 %). Moreover, attendees expressed expertise or substantial experience with a range of topics related to implementation from economics to large scale roll-out or scale-up, with the greatest number of attendees endorsing expertise in research design (*N* = 69, 30.4 %), organizational factors (*N* = 62, 27.3 %), and training (*N* = 66, 29.1 %). At least 69 attendees (30.4 %) had received funding outside of their institution to train others to implement an EBP or evaluate an EBP implementation. Attendees indicated expertise in or substantial experience with all eight implementation outcomes^v^ identified by Proctor et al. [21], with fidelity the most endorsed (*N* = 92, 40.5 %) and cost the least endorsed outcome (*N* = 16, 7.0 %). Finally, attendees have utilized various methods and designs to evaluate the implementation of an EBP. The most frequently endorsed methods and designs consisted of qualitative interview/focus groups (*N* = 115, 50.7 %), pre-post evaluation (*N* = 98, 43.2 %), surveys/standardized assessments (*N* = 91, 40.1 %), and needs assessments (*N* = 81, 35.7 %). Given that the majority of SIRC attendees also presented at the conference (51.7 %), these data largely reflect the expertise of the presenters at the 2015 conference.

**Future Directions for SIRC**

The Society for Implementation Research Collaboration (SIRC) is in the process of transitioning from its National Institute of Mental Health (NIMH) conference funding to society status. To support SIRC’s longevity, we began charging for membership^ii^ in January 2016 and achieved 161 founding members in the first month. SIRC is being led by an internally elected group of officers over the next two years to ensure a smooth transition from NIMH funding to an internally sustainable financial structure.^vi^ In 2017, SIRC members will be asked to nominate incoming officers and a formal vote will take place. In addition to planning for our next conference, SIRC has prioritized several initiatives to benefit its members and the field of implementation science as a whole.

**Journal**

As the field of implementation science grows, the number of publication outlets and their focus is unable to meet the demands of the innovative work being produced, particularly for those working in behavioral health. The Society for Implementation Research Collaboration (SIRC) proposes to establish an open access, double-blind peer-reviewed, online journal that publishes rigorous and pragmatic original empirical research related to methods of facilitating the implementation and sustainment of evidence-based practices (EBPs) in behavioral health policy and practice. An informal meeting was convened at SIRC 2015 to solicit feedback on the scope and focus of the proposed journal. Moving forward, SIRC will engage an international feedback initiative using web-based surveys to further focus the journal’s scope, assess general interest, as well as identify manuscript priorities and members interested in supporting the journal on the planning committee, advisory board, editorial board, as a reviewer, etc. Please visit our website for more information on the proposed journal.^vii^

**SIRC training institute for collaborative science (STICS)**

The Society for Implementation Research Collaboration (SIRC) is developing a collaborative research training institute that expands the scope of the implementation research workforce and promotes rigorous, locally-relevant and pragmatic science. Using interprofessional education and principles from the science of team science, STICS will train teams that include an evidence-based practice (EBP) champion, intermediary, and researcher with mentors from each role.

STICS is designed as a compliment to other implementation research training opportunities as it explicitly includes non-researchers in the process. An abbreviated version of STICS will be piloted in collaboration with leaders of the Australasian Implementation Network at their Biennial Australasian Implementation Conference in October 2016.

**SIRC resource library**

The Society for Implementation Research Collaboration (SIRC) is also developing a series of pages on the website which summarize and provide useful resources focused on specific implementation research topics. Initial areas of foci will include the best introductory materials for implementation science (already available), approaches to efficiently measuring fidelity across multiple evidence-based practices (EBPs), strategies for performance monitoring, etc. We will link relevant videos of SIRC conference presentations to each resource page as well as key articles and chapters, policy briefs, funding announcements, and website links. To maintain quality, all materials in the Resource Library will be created or recommended by members of the SIRC Network of Expertise.

**EBP champion task force**

Evidence-based practice (EBP) champions are clinician, administrative, and policy leaders, as well as intermediaries that have successfully implemented EBPs and are interested in linking with implementation researchers to support the practical relevance of the emerging training and implementation methods and evaluation strategies. The EBP Champion Task Force began in March of 2014 and includes Society for Implementation Research Collaboration (SIRC) officers (*N* = 7) and EBP champions (*N* = 8) who advise SIRC on how to maximize its relevance to EBP champions and to increase their involvement. The task force was critical in conference planning, for instance. Perspectives and interests from the task force led to broadening the score of the Implementation Development Workshop (IDW) to incorporate EBP champion projects, the integration of EBP champion and researcher symposium throughout the conference, and the development of the pre-conference workshops targeting issues of sustainability and progress monitoring. Between conferences, the task force will focus on growing the EBP champion membership and developing initiatives of high relevance to our EBP champion members.

**Instrument review project**

With new National Institute of Mental Health (NIMH) funding (R01MH106510), the Society for Implementation Research Collaboration (SIRC) will pursue its long-term goal to develop a comprehensive battery of reliable, valid, and pragmatic measures that researchers and stakeholders can use to advance the science and practice of implementation. The overarching objective of this project is to put forth a measurement-focused research agenda for implementation science (i.e., which constructs possess psychometrically strong and pragmatic measures, which require further development) as well as measures and methods to accomplish this work [22]. In addition to publishing the results of this project, the methods, measures, and results will be made available to SIRC members in our online repository. Results from our preliminary work are currently available on the members section of the SIRC website.^viii^

**Summary**

This supplement allowed for a compilation of the abstracts of the oral and poster presentations of the 2015 Society for Implementation Research Collaboration (SIRC) Conference, “Advancing Efficient Methodologies through Team Science and Community Partnerships,” in the service of advancing cumulative knowledge.^ix^ In addition to this supplement, SIRC aims to expand the reach and accessibility of the conference material to those who were not in attendance. SIRC maintains a large presence on social media with live Twitter updates during the conference (#SIRC2015) and announcements of SIRC initiatives between conferences (@implementcollab). We look forward to the 4^th^ Biennial SIRC conference, to be held in Seattle in September 2017.

List of Abbreviations SIRC: Society for Implementation Research Collaboration; EBP: Evidence-Based Practice; NIMH: National Institutes of Mental Health; IDW: Implementation Development Workshop; STICS: SIRC Training Institute for Collaborative Science

**Competing Interests**

There are no competing interests to report.

**Authors Contributions**

All authors (CL, DD, SK, MMD, SJL, ARL, CS, SD, JL, BM, AP, CND, KH, AP, PF, KC) contributed to activities that made this conference and the manuscript possible. Authors contributed to: website development; the creation of the member survey; data collection, cleaning, and analysis; designing the conference theme and activities; reviewing submitted abstracts; preparing and leading workshops; coordinating the conference and volunteers; leading task forces or initiatives; and drafting sections of the manuscript. All authors (CL, DD, SK, MMD, SJL, ARL, CS, SD, JL, BM, AP, CND, KH, AP, PF, KC) reviewed the manuscript, provided critical feedback, and approved the final version of the manuscript.

Acknowledgements

The preparation of this manuscript was supported, in kind, through the National Institute of Mental Health Award Number R13MH086159 granted to PI: KA Comtois. Research reported in this publication was also supported by the National Institute of Mental Health under Award Number R01MH106510 granted to PI: CC Lewis. Drs. Lyon and Dorsey are investigators with the Implementation Research Institute (IRI), at the George Warren Brown School of Social Work, Washington University in St. Louis, through an award from the National Institute of Mental Health under Award Number R25MH080916 and the Department of Veterans Affairs, Health Services Research & Development Service, Quality Enhancement Research Initiative (QUERI). This publication was also made possible in part by two Mentored Career Development Awards from the National Institute of Mental Health, awarded to Drs. Lyon (K08MH095939) and Locke (K01MH100199). Finally, Dr. Dorsey’s time on this project was supported through the National Institutes of Mental Health Award Number R01MH095749. The content is solely the responsibility of the authors and does not necessarily represent the official views of the National Institutes of Health.

Endnotes

^i^ Manuscript definitions can be found here. Behavioral Health Interventions: These are treatments targeted at mental health and substance use conditions as well as behavioral factors associated with physical symptoms and chronic illnesses. Team science is large-scale, team-based research built to target complex and multi-faceted problems that cannot be solved by a single discipline. Community partnerships refer to a collaboration among stakeholder groups and academics in the service of advancing a shared goal. Intermediaries are also known as trainers, internal and external facilitators, implementation practitioners and purveyors. Intermediaries provide training and consultation and otherwise assist community settings to implement evidence-based practices. Stakeholders are an individual, group, or organization who may affect, be affected by, or perceive itself to be affected by a decision, activity, or outcome of a project, program, or portfolio.

^ii^To become a SIRC member please go to: https://www.societyforimplementationresearchcollaboration.org/sirc-membership/

^iii^Attendees at the IDW were formed into groups by the conference co-directors (CCL & KAC) to strike a balance between new and established investigators as well as domestic and international representation. In addition, SIRC core members familiar to the format were widely dispersed across the three rooms. Finally, this was the first set of IDWs in which SIRC encouraged EBP champions to present developing projects. To accommodate this shift, the two EBP champion presenters were included in the same room, along with a higher portion of champion attendees. Each group consisted of an average of 15 attendees, each with four presenters (who served as attendees when not presenting their work).

^iv^SMART: Sequential, Multiple, Assignment Randomized Trial

^v^Proctor and colleagues presented eight implementation research outcomes: acceptability, adoption, appropriateness, feasibility, fidelity, implementation cost, penetration, and sustainability.

^vi^For more information on the organization structure of SIRC and specific officer positions please go to: https://www.societyforimplementationresearchcollaboration.org/what-is-sirc/

^vii^For more information on the proposed journal and the link to the survey please go to: https://www.societyforimplementationresearchcollaboration.org/sirc-projects/proposed-journal-behavioral-health-implementation-research/

^viii^For more information and results from our preliminary work with the Instrument Review Project please go to: https://www.societyforimplementationresearchcollaboration.org/sirc-projects/sirc-instrument-project/

^ix^Important to note is that the supplement represents a subset of the conference abstracts (63.24 %; 86 of 136). All authors were invited to have their abstracts included, but some had already published full manuscripts of the presented work; other presentation abstracts did not include data or were summaries of several completed studies that did not fit the structured abstract format, and a few authors declined due to their work not yet being advanced enough for inclusion of data for publication.

**References**

1. World Health Organization. Investing in Mental Health. Geneva: WHO. 2003.

2. Kieling C, Baker-Henningham H, Belfer M, Conti G, Ertem I, et al. Child and adolescent mental health worldwide: evidence for action. The Lancet. 2001;378:1515-25.

3. Eccles MP, Mittman BS. Welcome to implementation science. Implement Sci. 2006;1:1- 3.

4. Glasgow RE, Vinson C, Chambers D, Khoury MJ, Kaplan RM, Hunter C. National Institutes of Health approaches to dissemination and implementation science: current and future directions. Am J of Pub Health. 2012;102:1274-81.

5. Marriott BR, Rodriguez AL, Landes SJ, Lewis CC, Comtois KA. A methodology for enhancing implementation science proposals: Comparison of Face-to-Face versus Virtual Workshops. Implement Sci. A83.

6. PsychoSocial Aspects of Diabetes Group. Behavioral Research in Diabetes Group Exchange. http://www.psad-easd.eu/bridge/. Accessed 11 March 2016.

7. Johnson K, Hays C, Center H, Daley C. Building capacity and sustainable prevention innovations: A sustainability planning model. Eval and Pro Planning. 2004;27:135-49.

8. Palinkas LA. A behavioral economic perspective on adoption, implementation, and sustainment of evidence-based interventions. Implement Sci. A1.

9. Chamberlain P. Towards making scale up of EBPs in child welfare systems more efficient and affordable. Implement Sci. A2.

10. Aarons GA, Green AE, Ehrhart MG, Trott EM, Willging CE. Mixed method examination of strategic leadership for evidence-based practice implementation. Implement Sci. A3.

11. Fernández ME, Woolf NH, Liang S, Heredia NI, Kegler M, et al. Implementing practice change in federally qualified health centers: learning from leaders’ experiences. Implement Sci. A4.

12. Damschroder LJ, Lowery JC. Efficient synthesis: using qualitative comparative analysis (QCA) and the CFIR across diverse studies. Implement Sci. A5.

13. Monroe-DeVita M, Peterson R, Darnell D, Berliner L, Dorsey S, Murray LK. Taking global local: Evaluating training of Washington State clinicians in a modularized CBT approach designed for low-resource settings. Implement Sci. A36.

14. Bolton P, Lee C, Haroz EE, Murray L, Dorsey S, et al. A transdiagnostic community-based mental health treatment for comorbid disorders: development and outcomes of a randomized controlled trial among Burmese refugees in Thailand. PLoS Med. 2014 Nov 11; 11(11):e1001757.

15. Barwick M, Barac R, Zlotkin S, Salim L, Marnie D. Factors implicated in successful implementation: evidence to inform improved implementation from high and low income countries. Implement Sci. A52.

16. Rubin RM, Ray ML, Wandersman A, Lamont A, Hannah G, et al. Procurement and contracting as an implementation strategy: Getting to outcomes contracting. Implement Sci. A80.

17. Saldana L, Schaper H, Campbell M, Chamberlain P. Web-based feedback to aid successful implementation: The interactive Stages of Implementation Completion (SIC) tool. Implement Sci. A81.

18. Meza RD, Dorsey S, Wiltsey Stirman S, Sedlar G, Lucid L. Dabblers, bedazzlers, or total makeovers: clinician modification of a common elements CBT approach. Implement Sci. A42.

19. Cabassa LJ, Gomes AP, Meyreles Q, Capitelli L, Younge R, et al. Using the collaborative intervention planning framework to adapt a health-care manager intervention to a new population and provider group to improve the health of people with serious mental illness. Implement Sci. 2014;9:178.

20. Smith JD. Observational assessment of fidelity to a family-centered prevention program: Differentiation and predictive validity. Implement Sci. A78.

21. Proctor E, Silmere H, Raghavan R, Hovmand P, Aarons G, Bunger A, Griffey R, Hensley M. Outcomes for implementation research: conceptual distinctions, measurement challenges, and research agenda. Adm Policy Ment. Health. 2011:38(2):65-76.

22. Lewis CC, Stanick CF, Martinez RG, Weiner BJ, Kim M, et al. The Society for Implementation Research Collaboration Instrument Review Project: A methodology to promote rigorous evaluation. Implement Sci. 2015;10:2.

## A1 A behavioral economic perspective on adoption, implementation, and sustainment of evidence-based interventions

### Lawrence A. Palinkas (palinkas@usc.edu)

#### Department of Children Youth and Families, School of Social Work, University of Southern California, Los Angeles, CA, 90089, USA

**Background**

Current models of evidence-based intervention (EBI) implementation offer an “etic” approach to understanding and identifying potential facilitators and barriers to adoption, implementation and sustainment. However, these models do not necessarily reflect the “emic” priorities or decision- making processes of clinic, agency, and systems leaders. This study drew upon two mixed methods investigations of EBI implementation, one for child mental health in New York State and one for HIV prevention in Mexico, to illustrate the application of principles of behavioral economics in understanding how and why EBIs are adopted, implemented, and sustained.

**Materials and methods**

Semi-structured interviews were conducted with 76 CEOs and program directors of 34 mental health clinics in New York State and 121 directors and staff of 12 reproductive health services clinics in Mexico. Transcripts were analyzed using a grounded theory approach to identify predominant themes related to implementation progress and effectiveness.

**Results**

In both settings, the decision to adopt, implement, or sustain EBIs was based on stakeholder assessments of implementation costs and benefits, capacity to implement, and acceptability of the EBI to the organization, service providers and the clients served. Analysis of qualitative data also revealed the application of six different principles of behavioral economics in decision-making processes: temporal discounting, loss aversion, monetary incentives, use of heuristics, decision fatigue, framing, and external influences. However, both the assessment and application were local context-dependent.

**Conclusions**

Emic models of local stakeholder priorities and decision-making processes overlap with etic or global implementation models and frameworks, but help to explain contextual influences.

## A2 Towards making scale up of evidence-based practices in child welfare systems more efficient and affordable

### Patricia Chamberlain (pattic@oslc.org)

#### Oregon Social Learning Center, Eugene, OR, 97401, USA

**Background**

Experiences in two states to scale up multiple evidence-based practices (EBPs) in child welfare were described. Lessons learned in state #1 informed efforts in state #2.

**Materials and methods**

The state #2 implementation was streamlined: 1) A brief foundational training covering EBP principles was provided to the entire child welfare workforce as a strategy for achieving culture change, 2) Intensive training was provided to select case workers, whereas in state #1, all caseworkers received the intensive training, 3) An integrated method to monitor fidelity and promote quality improvement was used [1]. Monthly reports to counties and system leadership detailed key outcomes including reach, fidelity, client engagement, and staff participation in consultation sessions. A full transfer strategy [2], from developers to case workers, was used to build sustainability. The full transfer method had successful caseworkers in cohort 1 trained as local coaches thereby assuming the previous functions of the developers. Local coaches received additional training; initially, their activities were “shadowed” by developers. System administrative data and a telephone checklist were used as to monitor key outcomes such as length of stay in foster care and disruption from placement homes.

**Results**

In state #1, caseworkers and casework supervisors (*N* = 250) were intensively trained over a nine-month period to deliver the EBPs. In state #2, thus far 280 caseworkers and supervisors have received foundational training and 58 have received intensive training over an eight-month period in the EBPs.

**Conclusions**

Implementation strategies can be streamlined to decrease cost, increase efficiency, and promote sustainability**.**

**References**

1. Chamberlain P, Feldman SW, Wulczyn F, Saldana L, Forgatch M. Implementation and evaluation of linked parenting models in a large urban child welfare system. Child Abuse Negl. 2015 Oct 23.

2. Forgatch MS, DeGarmo DS. Sustaining fidelity following the nationwide PMTO™ implementation in Norway. Prev Sci. 2011 Sep 1; 12(3):235-46.

## A3 Mixed method examination of strategic leadership for evidence-based practice implementation

### Gregory A. Aarons^1,2^ (gaarons@ucsd.edu), Amy E. Green^1,2^, Mark G. Ehrhart^3^, Elise M. Trott^4^, Cathleen E. Willging^4^

#### ^1^Department of Psychiatry, University of California, San Diego, La Jolla, CA, 92093, USA; ^2^Child and Adolescent Services Research Center (CASRC), San Diego, CA, 92123, USA; ^3^Department of Psychology, San Diego State University, San Diego, CA, 92182, USA; ^4^Pacific Institute for Research and Evaluation, Behavioral Health Research Center of the Southwest, Albuquerque, NM, 87102, USA

##### **Correspondence:** Gregory A. Aarons (gaarons@ucsd.edu) – Child and Adolescent Services Research Center (CASRC), San Diego, CA, 92123, USA

**Background**

Leadership that supports effective evidenced-based practice (EBP) implementation and sustainment is a critical concern. The recently developed Implementation Leadership Scale (ILS) [1] is a valid and reliable 12-item scale with four subscales: proactive leadership, knowledgeable leadership, supportive leadership, and perseverant leadership. The ILS factor structure was developed using exploratory factor analysis (EFA) and supported using confirmatory factor analysis (CFA) with a sample of 459 mental health clinicians.

**Materials and methods**

In the current study, we analyzed quantitative and qualitative data from a large mixed-method study of EBP sustainment to examine the utility and structure of the ILS. Participants included home visitors from 25 community-based organizations across 10 child welfare service systems implementing the EBP SafeCare® to prevent child neglect. Home visitors (*N* = 190) completed the ILS as part of an annual web-survey during the same year qualitative focus groups (*N* = 18) were conducted, focusing on implementation and sustainment of SafeCare. During focus groups, home visitors were asked to respond to the prompt, “How have leaders influenced the ongoing use of SafeCare?”

**Results**

A CFA of the ILS confirmed the original factor structure. Qualitative data supported the four ILS subscales. The theme of “accessible leadership” emerged from the qualitative data and is an area for future research.

**Conclusions**

The dimensions of implementation and sustainment leadership are similar, and consistent across the Exploration, Preparation, Implementation, Sustainment (EPIS) implementation framework phases [2]. Thus, the ILS may be useful in examining and supporting both implementation and sustainment.

**References**

1. Aarons GA, Ehrhart MG, Farahnak LR. The Implementation Leadership Scale (ILS): Development of a brief measure of unit level implementation leadership. Implement Sci. 2014;9(1):45.

2. Aarons GA, Hurlburt M, Horwitz SM. Advancing a conceptual model of evidence-based practice implementation in public service sectors. Adm Policy Ment Hlth. 2011;38(1):4-23.

## A4 Implementing practice change in Federally Qualified Health Centers: Learning from leaders’ experiences

### Maria E. Fernandez^1^ (maria.e.fernandez@uth.tmc.edu), Nicholas H. Woolf^2^, Shuting (Lily) Liang^3^, Natalia I. Heredia^1^, Michelle Kegler^3^, Betsy Risendal^4^, Andrea Dwyer^4^, Vicki Young^5^, Dayna Campbell^5^, Michelle Carvalho^3^, Yvonne Kellar-Guenther^4^

#### ^1^School of Public Health, University of Texas Health Science Center at Houston, Houston, TX, 77030, USA; ^2^Woolf Consulting, Santa Barbara, CA, 93102, USA; ^3^Rollins School of Public Health, Emory University, Atlanta, GA, 30322, USA; ^4^University of Colorado Cancer Center at Denver, Aurora, CO, 80045, USA; ^5^South Carolina Primary Health Care Association, Columbia, SC, 29203, USA

##### **Correspondence:** Maria E. Fernandez (maria.e.fernandez@uth.tmc.edu) – School of Public Health, University of Texas Health Science Center at Houston, Houston, TX, 77030, USA

**Background**

With changes related to the Affordable Care Act and other initiatives, Federally Qualified Health Centers (FQHCs) are experiencing increased pressure to implement practice changes. We explored factors influencing the implementation of evidence-based interventions for cancer prevention and control in FQHCs.

**Materials and methods**

We conducted a qualitative study of FQHC leaders (*N* = 59) who described their experiences implementing evidence-based practices. We asked questions about inner and outer setting variables using a modified Appreciative Inquiry approach. We conducted grounded and thematic analyses of barriers and facilitators, and identified levers of change most useful for practice change using ATLAS.ti.

**Results**

Leaders reported factors influencing successful implementation of change, including necessary and sufficient staff and leadership characteristics; the role of mandates, financial consequences, and leaders’ personal passions in prioritizing change; and the significance of external relationships and collaborations. Remaining challenges included staff knowledge and capacity; the impact of practice change on existing provider and staff time constraints; and the continuing need for automated and systematic procedures.

**Conclusions**

Analysis revealed lessons from success and challenges, the interaction of individual and organizational factors in each area, and the potential of electronic medical records. Findings can be used in implementation interventions by helping define implementation actors and activities, identifying determinants of these behaviors, and identifying methods to improve the implementation.

## A5 Efficient synthesis: Using qualitative comparative analysis and the Consolidated Framework for Implementation Research across diverse studies

### Laura J. Damschroder^1,2^, Julie C. Lowery^1,2^

#### ^1^Veterans Affairs (VA) Ann Arbor Center for Clinical Management Research, Ann Arbor, MI, 48105, USA; ^2^Personalizing Options through Veteran Engagement (PROVE) Quality Enhancement Research Initiative (QUERI) Program, Ann Arbor, MI, 48105, USA

##### **Correspondence:** Laura J. Damschroder (laura.damschroder@va.gov) – Personalizing Options through Veteran Engagement (PROVE) Quality Enhancement ResearchInitiative (QUERI) Program, Ann Arbor, MI, 48105, USA

**Background**

Syntheses are needed to understand what works where and why across diverse implementation studies. However, even with increasing numbers of published syntheses of organizational interventions, most highlight gaps in knowledge of contextual factors that influence implementation success.

**Materials and methods**

We synthesized findings from six implementation studies of different programs that all systematically assessed context using the Consolidated Framework for Implementation Research (CFIR). Qualitative Comparative Analysis (QCA) methods were used to analyze ratings and outcomes data from program implementations at 53 Veterans Affairs medical centers.

**Results**

Many CFIR constructs had missing ratings. Only 16 of the 39 CFIR constructs had coded ratings across a majority of the 53 cases. Combinations of constructs leading to successful implementation varied depending on the combination of constructs included in the analyses.

Taking time to reflect and evaluate during implementation, compatibility with clinical processes and values, and not having negative ratings of leadership engagement were most commonly associated with success.

**Conclusions**

Use of the CFIR within an individual studies enabled a synthesis across studies using QCA methods. The CFIR offers a means of standardizing definitions of key constructs across studies, while QCA acknowledges the interactive and complex influence of context on implementation success in a way that is impossible to do using traditional correlation-based statistical approaches. A growing repository of cases, all using a consistent framework, can help to identify complex pathways to success across diverse contexts.

**Acknowledgments**

This study was funded by the Department of Veterans Affairs (VA), Diabetes Quality Enhancement Research Initiative (QUERI). The contents do not represent the views of the U.S. Department of Veterans Affairs or the United States Government.

## A6 Establishing a veterans engagement group to empower patients and inform Veterans Affairs (VA) health services research

### Sarah S. Ono^1,2,3^, Kathleen F. Carlson^1,4^, Erika K. Cottrell^1,2^, Maya E. O’Neil^1,5,6^, Travis L. Lovejoy^1,5,7^

#### ^1^Center to Improve Veteran Involvement in Care (CIVIC), Veterans Affairs (VA) Portland Health Care System (VAPORHCS), Portland, OR, 97239, USA; ^2^Department of Family Medicine, Oregon Health & Science University, Portland, OR, 97239, USA; ^3^Division of General Internal Medicine, Department of Medicine, Oregon Health & Science University, Portland, OR, 97239, USA; ^4^Division of Epidemiology, School of Public Health, Oregon Health & Science University, Portland, OR, 97239, USA; ^5^Department of Psychiatry, Oregon Health & Science University, Portland, OR, 97239, USA; ^6^Department of Medical Informatics & Clinical Epidemiology, Oregon Health & Science University, Portland, OR, 97239, USA; ^7^School of Public Health, Oregon Health & Science University, Portland, OR, 97239, USA

##### **Correspondence:** Sarah S. Ono (sarah.ono@va.gov) – Division of General Internal Medicine, Department of Medicine, Oregon Health & Science University, Portland, OR, 97239, USA

**Background**

In 2013, Department of Veterans Affairs (VA) funded 19 Centers of Innovation (COINs), each with unique research foci and partnerships between researchers, clinicians, and operations leaders. Portland VA’s COIN, the Center to Improve Veteran Involvement in Care (CIVIC), emphasizes community based participatory research principles and patient engagement in research at all stages. To support this goal CIVIC set out to implement a veteran engagement group (VEG).

**Materials and methods**

CIVIC established a VEG, now composed of seven Veteran patients, using an approach we called a “seed committee” informed by influences ranging from anthropology to agriculture. The seed committee involved five well-connected community members who provided feedback on early VEG planning and facilitated the recruitment process. These were individuals with limited time, but positioned to refine recruitment materials and identify potential VEG members during a limited commitment (4 months).

**Results**

The resulting group of patients assembled offers feedback to CIVIC investigators on study conduct and dissemination of results, a process we are actively tracking to better understand the bi-directional goals and impacts of this work. We describe our process of implementing the VEG using a novel interim seed committee and address issues germane to participatory research in VA and non-VA settings, such as ensuring a representative group composition, navigating institutional review boards, compensation for members, and maintaining sound research ethics.

**Conclusions**

At a time when there is growing promotion of patient engagement, there is also a growing need for models to demonstrate this engagement in action. The implementation of the CIVIC VEG offers one such example.

## A7 Building patient-practitioner partnerships in community oncology settings to implement behavioral interventions for anxious and depressed cancer survivors

### Joanna J. Arch^1^, Jill L. Mitchell^2^

#### ^1^Department of Psychology and Neuroscience, University of Colorado Boulder, Boulder, CO, 80309, USA; ^2^Rocky Mountain Cancer Centers-Boulder, Boulder, CO, 80303, USA

##### **Correspondence:** Joanna J. Arch (joanna.arch@colorado.edu) – Department of Psychology and Neuroscience, University of Colorado Boulder, Boulder, CO, 80309, USA

**Background**

Many cancer survivors with anxiety and depression symptoms are treated in oncology care settings that lack systems to recognize such symptoms or offer behavioral interventions.

**Materials and methods**

Over the past four years we have fostered a collaboration with the administrators, providers, and patients at a 21-office community oncology care network to address this challenge. We initially targeted a network office site and provider team that championed external collaborations, to establish screening procedures and conduct a pilot study.

**Results**

We aligned our goal to implement a screener for anxiety and depression symptoms among cancer survivors with the site’s goal to implement a distress screener at survivorship appointments (*N* > 200 screened to date). Upon establishing a successful screening system, we partnered with an onsite social worker champion (J.L.M.) to develop and evaluate a behavioral intervention for positively screened patients (*N* = 51), in a format and length adapted to the needs of the site, with content iteratively refined in response to patient feedback. The intervention showed large effects on anxiety and depression outcomes. We communicated these findings to much of the network, thus building support from network administrative, physician, and social work teams to identify anxious and depressed patients, implement the intervention, and recently, to conduct a funded clinical trial in the network, using patient screening and recruitment strategies tailored to the needs and capacities of each site.

**Conclusions**

Our work demonstrates one approach to partnering with a community-based cancer care network to implement a behavioral intervention that addresses anxiety and depression among cancer survivors.

## A8 Tailoring a Cognitive Behavioral Therapy implementation protocol using mixed methods, conjoint analysis, and implementation teams

### Cara C. Lewis^1,2^, Brigid R. Marriott^1,3^, Kelli Scott^1^

#### ^1^Department of Psychological and Brain Sciences, Indiana University, Bloomington, IN, 47405, USA; ^2^Department of Psychology, University of Washington, Seattle, WA, 98195, USA; ^3^Department of Psychological Sciences, University of Missouri, Columbia, MO, 65211, USA

##### **Correspondence:** Cara C. Lewis (clewis11@uw.edu) – Department of Psychology, University of Washington, Seattle, WA, 98195, USA

**Background**

A recent Cochrane review revealed that tailored implementation outperformed standardized approaches [1]. However, few tailoring methodologies exist. This study presents data from a mixed-methods prospective tailored implementation of Cognitive Behavioral Therapy (CBT) in youth residential settings.

**Materials and methods**

Clinicians and staff completed surveys (*N* = 70) and participated in focus groups (*N* = 53) guided by the Framework of Dissemination Context of Diffusion [2] as part of a needs assessment.

Mixed methods analysis revealed 76 unique contextual barriers. Administrators prioritized barriers according to feasibility and importance. These prioritized barriers (*N* = 23) were subjected to a conjoint analysis wherein implementation strategies were collaboratively selected by researchers and implementation team members. Researchers rated strategies based on feasibility and impact on CBT adherence [3]. Strategies (*N* = 36) were matched with prioritized barriers to form an implementation blueprint. Implementation teams led strategy enactment prior to CBT implementation. The needs assessment surveys were re-administered to clinicians and staff (*N* = 49) at one year follow up.

**Results**

Wilcoxon Signed Rank Tests comparing the two assessment points within individuals (*N =* 16) revealed significant improvements (*p* < .05) across four determinants of practice (e.g., teamwork, staff efficacy). Mann Whitney U Tests comparing the two assessments in the independent groups (*N =* 84) revealed significant improvements (*p* < .05) across 24 determinants (e.g., efficacy, community) and a decline in one determinant (openness to new practices).

**Conclusions**

Needs assessment and conjoint analysis procedures enabled prioritization and selection of strategies to address barriers prior to CBT implementation. The work of the implementation teams resulted in improvements in the majority of the initially identified determinants.

**References**

1. Baker R, Camosso-Stefinovic J, Gillies C, Shaw EJ, Cheater F, Flottorp S, Robertson N. Tailored interventions to overcome identified barriers to change: effects on professional practice and health care outcomes. Cochrane Database Syst Rev. 2010 Mar 1; 3(3).

2. Mendel P, Meredith LS, Schoenbaum M, Sherbourne CD, Wells KB. Interventions in organizational and community context: a framework for building evidence on dissemination and implementation in health services research. Adm. Policy Ment Health 2008; 35:21–37.

3. Wensing M, Oxman A, Baker R, Godycki-Cwirko M, Flottorp S, Szecsenyi J, Grimshaw J, Eccles M. Tailored implementation for chronic diseases (TICD): a project protocol. Implement Sci. 2011 Sep 7; 6:103.

## A9 Wraparound Structured Assessment and Review (WrapSTAR): An efficient, yet comprehensive approach to Wraparound implementation evaluation

### Jennifer Schurer Coldiron, Eric J. Bruns, Alyssa N. Hook

#### Division of Public Behavioral Health and Justice Policy, University of Washington, Seattle, WA, 98102, USA

##### **Correspondence:** Jennifer Schurer Coldiron (jscold@uw.edu) – Division of Public Behavioral Health and Justice Policy, University of Washington, Seattle, WA, 98102, USA

**Background**

Wraparound [1] is a well-established model for care coordination for youth with complex emotional and behavioral needs and their families. While several measures of fidelity and outcomes exist, they have been used sporadically by the field and have, until now, been used in isolation, providing little in the way of comprehensive information for improvement.

**Materials and methods**

We synthesized extant Wraparound guidelines and consulted national experts and the implementation science literature. Forty-five indicators of high-quality practice across four domains—outcomes, fidelity, implementation, and system support—were created, and a measurement strategy for each was developed, harnessing validated measures when available. The resulting protocol, the Wraparound Structured Assessment and Review (WrapSTAR), was then piloted and further refined.

**Results**

An initial pilot indicates that the protocol is feasible, with minimal burden to provider personnel, and yields actionable information for stakeholders that can be used to develop targeted quality improvement efforts. Recent experiences taking the protocol to scale in one state and teaching another state to conduct the review independently provide further evidence of the approach's utility and efficiency.

**Conclusions**

A comprehensive and external review of a Wraparound provider organization is useful and feasible. Additionally, there may be an opportunity to use the indicators to develop a self- assessment toolkit, further widening the protocol’s application.

**References**

1. Bruns EJ, Walker JS, Zabel M, Matarese M, Estep K, Harburger D, Mosby M, Pires SA. Intervening in the lives of youth with complex behavioral health challenges and their families: The role of the wraparound process. Am J Community Psychol. 2010 Dec 1; 46(3-4):314-31.

## A10 Improving the efficiency of standardized patient assessment of clinician fidelity: A comparison of automated actor-based and manual clinician-based ratings

### Benjamin C. Graham, Katelin Jordan

#### National Center for Posttraumatic Stress Disorder (PTSD), Dissemination and Training Division, Veterans Affairs (VA) Palo Alto Health Care System, Menlo Park, CA, 94304, USA

##### **Correspondence:** Benjamin C. Graham (benjamincgraham@gmail.com) – National Center for Posttraumatic Stress Disorder (PTSD), Dissemination and Training Division, Veterans Affairs (VA) Palo Alto Health Care System, Menlo Park, CA, 94304, USA

**Background**

Assessment of skills following evidence-based practice (EBP) training is of critical importance to dissemination. Standardized patient (SP) methodology offers more ecologically valid measurement compared to questionnaires, but is manually-conducted, time-consuming and un-scalable. Clinician behavior is dynamic and challenging to code, whereas scripted actor statements are not. Automated, actor-based scoring may offer a parsimonious yet effective SP scoring method. This presentation compares automated actor-based scoring to manual clinician- based rating of transcribed SP interviews, based on a training study of mental health clinicians (*N* = 420) treating veterans with post-traumatic stress disorder (PTSD).

**Materials and methods**

This study compared inter-rater reliability within a five-person team of raters to an automated approach based on actor statements. Keywords and phrases were entered into scoring algorithms for six targeted skill areas related to chain analysis/case formulation. Automated scoring paralleled manual rating, allowing for comparison of the method to manual ratings as an additional ‘team member’.

**Results**

Across six skill areas, the traditional rating team established inter-rater reliability on all six criteria (Gwet’s AC1 = .84-.96). When the automated method was included, it performed as an adequate, if not exemplary, ‘team member’ on five of the six skills (Gwet’s AC1 = .71-.93) but failed in one (Gwet’s AC1 = .41). A preliminary cost analysis suggests that this approach can greatly reduce costs of SP assessment.

**Conclusions**

The use of technology-based assessment of skills following training is an important frontier in the promotion of EBPs. While not all skill areas warrant automation, results suggest some skills can be appraised parsimoniously via automation. In general, automated scoring performed best for skills assessing specific rather than general experiences (e.g., asking a client about their bodily sensations vs. the general external events preceding a problem behavior). This study provides methodological support for broader efforts to incorporate technology in the rapid appraisal of training.

**Acknowledgements**

Congressionally Directed Medical Research Programs, Randomized Controlled Trial of Cognitive Behavioral Therapy Training for Posttraumatic Stress Disorder Providers [W81XWH-12-1-0531], Josef Ruzek (Co-PI) & Raymond Rosen (Co-PI)

## A11 Measuring fidelity on the cheap

### Rochelle F. Hanson, Angela Moreland, Benjamin E. Saunders, Heidi S. Resnick

#### Department of Psychiatry and Behavioral Sciences, National Crime Victims Research and Treatment Center, Medical University of South Carolina, Charleston, SC, 29412, USA

##### **Correspondence:** Rochelle F. Hanson (hansonrf@musc.edu) – Department of Psychiatry and Behavioral Sciences, National Crime Victims Research and Treatment Center, Medical University of South Carolina, Charleston, SC, 29412, USA

**Background**

One significant challenge to implementation researchers is determining a cost-effective, yet reliable and valid measure of treatment fidelity [1]. While observational measurement represents the ‘gold standard,’ such methods are expensive, time consuming, and generally not feasible or sustainable in community-based settings. Research is needed to examine whether self-report adherence measures are feasible and can yield useful information for training and implementation studies. This presentation examined data on clinician self-reported use of a trauma-focused, evidence-based treatment, Trauma-Focused Cognitive Behavioral Therapy (TF-CBT) [2] throughout participation in a learning collaborative (LC) [3] and its relationship with outcomes (post-traumatic stress disorder and depression) [4-6].

**Materials and methods**

A total of 311 clinicians from eight TF-CBT LCs attended a first training session. Training required completion of at least two TF-CBT cases; weekly, online TF-CBT use metrics; and administration of pre- and post-treatment measures of post-traumatic stress disorder (PTSD) and depression.

**Results**

A total of 388 cases had pre-post data and at least one weekly metric (weekly metrics completed per case *M* = 10.97; *SD* = 5.03). Clinicians completed an average of 8.86 (of 11) TF-CBT components and at least 10/11 components with 50.8 % of clients. Self-reported use of the overall model, as well as trauma narrative, in vivo mastery, and enhancing safety components were significantly related to pre-post treatment declines in PTSD and depression (*p* < .05).

**Conclusions**

Despite study limitations (i.e., lack of comparison condition, reliance on self-report), positive associations between self-reported use of TF-CBT and patient treatment outcomes yield promising directions for measuring treatment fidelity in a cost-effective, feasible, and sustainable manner.

**References**

1. Schoenwald S, Garland A. A review of treatment adherence measurement methods. Psychol Assessment. 2013; 25:146-156.

2. Cohen JA, Mannarino AP, Deblinger E. Treating trauma and traumatic grief in children and adolescents: A clinician's guide. Guilford Press; 2006.

3. Saunders BE, Hanson RF. Treatment of child abuse: Common ground for mental health, medical, and legal practitioners. 2^nd^ ed. JHU Press; 2014. 235-45 p.

4. Sternberg AM, Brymer MJ, Decker KB, Pynoos RS. The University of California at Los Angeles Post-traumatic Stress Disorder Reaction Index. Curr Psychiatry Rep. 2004; 6:96- 100.

5. Angold A, Costello EJ, Messer SC, Pickles A, Winder F, Silver D. The development of a short questionnaire for use in epidemiological studies of depression in children and adolescents. Int J Method Psych. 1995; 5:237-49.

6. Foa EB, Johnson KM, Feeny NC, Treadwell KRH. The Child PTSD Symptom Scale: A preliminary examination of its psychometric properties J Clin Child Adolesc. 2001; 30(3):376-84.

## A12 Leveraging routine clinical materials to assess fidelity to an evidence-based psychotherapy

### Shannon Wiltsey Stirman^1^, Cassidy A. Gutner^2^, Jennifer Gamarra^3^, Dawne Vogt^2^, Michael Suvak^4^, Jennifer Schuster Wachen^2^, Katherine Dondanville^5^, Jeffrey S. Yarvis^6^, Jim Mintz^5,7^, Alan L. Peterson^5,8^, Elisa V. Borah^9^, Brett T. Litz^2^, Alma Molino^5^, Stacey Young McCaughan^5,^ Patricia A. Resick^10^

#### ^1^National Center for Posttraumatic Stress Disorder (PTSD), Veterans Affairs (VA) Palo Alto Health Care System and Stanford University, Menlo Park, CA, 94024, USA; ^2^National Center for PTSD, VA Boston Healthcare System and Boston University, Boston, MA, 02130, USA; ^3^Department of Psychology, University of California, Los Angeles, Los Angeles, CA, 90095, USA; ^4^Department of Psychology, Suffolk University, Boston, MA, 02108, USA; ^5^Department of Psychiatry, The University of Texas Health Science Center at San Antonio, San Antonio, TX, 78229, USA; ^6^Headquarters, Carl R. Darnall Army Medical Center, Fort Hood, TX, 76544, USA; ^7^Department of Epidemiology and Biostatistics, The University of Texas Health Science Center at San Antonio, San Antonio, TX, 78229, USA; ^8^Office of Research and Development, South Texas Veterans Health Care System, San Antonio, TX, 78229, USA; ^9^School of Social Work, The University of Texas at Austin, Austin, TX, 78712, USA; ^10^Department of Psychiatry and Behavioral Sciences, Duke University Medical Center, Durham, NC, 27701, USA

##### **Correspondence:** Shannon Wiltsey Stirman (sws1@stanford.edu) – National Center for Posttraumatic Stress Disorder (PTSD), Veterans Affairs (VA) Palo Alto Health Care System and Stanford University, Menlo Park, CA, 94024, USA

**Background**

Fidelity monitoring and support is a central component of many implementation models. A critical barrier to efforts to monitor and support treatment fidelity in routine care settings and large systems is a lack of availability of feasible, scalable, and valid fidelity measurement strategies [1]. Development of reliable, low-burden methods of fidelity assessment is an important step in promoting sustained implementation fidelity for complex interventions in routine care.

**Materials and methods**

We developed a system to assess fidelity (adherence and competence) in an evidence-based psychotherapy by rating clinical notes and worksheets. External raters assessed clinical notes, along with worksheets that were completed with therapist guidance within sessions. Worksheets completed independently by clients for homework were also rated to differentiate between therapist and clients’ contributions to worksheet quality. We examined feasibility, efficiency, reliability, criterion-related validity (correlation with observer ratings of session video), and predictive validity (whether ratings predicted symptom change) using data from a clinical trial of Cognitive Processing Therapy conducted in a military setting (*N* = 106).

**Results**

The rating system required an average of seven minutes per session (versus 50-60 for video observation). Intra-class correlations indicated good to excellent rater agreement. Adherence and competence ratings were highly correlated with observer ratings for worksheet-related items.

Symptoms did not predict subsequent therapist fidelity, but therapist fidelity in certain sessions predicted subsequent symptom change. Client skill on homework worksheets did not predict subsequent symptom change.

**Conclusions**

This system of assessing fidelity using routine clinical materials has potential as a reliable, valid, efficient, and scalable fidelity monitoring strategy.

**Acknowledgements**

This project was supported through a grant from the National Institutes of Health, R21 MH 099169 (Shannon Wiltsey Stirman). A portion of this work was supported by funding to the STRONG STAR Consortium by the U.S. Department of Defense through the U.S. Army Medical Research and Materiel Command, Congressionally Directed Medical Research Programs, Psychological Health and Traumatic Brain Injury Research Program awards W81XWH-08-02-109 (Alan Peterson), W81XWH-08-02-0114 (Brett Litz) and W81XWH-08-

02-0116 (Patricia Resick). The views expressed herein are solely those of the authors and do not reflect an endorsement by or the official policy of the U.S. Army, the Department of Defense, the Department of Veterans Affairs, or the U.S. Government.

**Reference**

1. Schoenwald SK, Garland AF, Chapman JE, Frazier SL, Sheidow AJ, Southam-Gerow MA. Toward the effective and efficient measurement of implementation fidelity. Admn Policy Ment Health. 2011 Jan 1; 38(1):32-43.

## A13 The video vignette survey: An efficient process for gathering diverse community opinions to inform an intervention

### Nancy Pandhi^1^, Nora Jacobson^2^, Neftali Serrano^3^, Armando Hernandez^4^, Elizabeth Zeidler-Schreiter^5^, Natalie Wietfeldt^1^, Zaher Karp^1^

#### ^1^Department of Family Medicine and Community Health, University of Wisconsin-Madison, Madison, WI, 53715, USA; ^2^Institute for Clinical and Translational Research and School of Nursing, University of Wisconsin-Madison, Madison, WI, 53705, USA; ^3^Foundation for Health Leadership & Innovation, Cary, NC, 27513, USA;^4^Group Health Cooperative of South Central Wisconsin, Madison, WI, 53703, USA; ^5^Access Community Health Centers, Madison, WI, 53713, USA

##### **Correspondence:** Nancy Pandhi (nancy.pandhi@fammed.wisc.edu) – Department of Family Medicine and Community Health, University of Wisconsin-Madison, Madison, WI, 53715, USA

**Background**

Gathering diverse community opinions to inform interventions is highly desirable. Methods that produce quality results while remaining timely and cost-effective are needed. The study’s objective was to use a video vignette survey to elicit perceptions of two models integrating behavioral health care into primary care.

**Materials and methods**

Working closely with behavioral heath and primary care leaders at three health systems, scripts depicting two fully integrated behavioral health models were developed. Various stakeholders, including a community advisory group drawn from nontraditional research populations, vetted preliminary videos. Final videos using local actors were embedded in a survey disseminated online and in-person via tablet computers. Participants viewed a single video matching the model used by their self-identified health system’s model. The survey asked three open-ended questions about likes, dislikes, and desired outcomes.

**Results**

The survey was completed by 381 individuals. Thirty percent responded online, 43 % in clinic waiting rooms, and 27 % at community locations. Thirty-five percent identified as low income, 28 % as non-white, and 44 % as having a mental health diagnosis. Content analysis of responses identified preferences were categorized in four domains: access to care, care experience, future services, and dignity. Concern about screening questionnaires and behavioral health provider type differed between the two models. This process took seven months and non-staff costs totaled ~ $7,000.

**Conclusions**

Video vignette surveys are a promising, efficient method for gathering diverse community perspectives to inform intervention design.

**Acknowledgements**

This project was supported by the Clinical and Translational Science Award (CTSA) program, through the National Institute of Health (NIH) National Center for Advancing Translational Sciences (NCATS), grant UL1TR000427.

## A14 Using integrated administrative data to evaluate implementation of a behavioral health and trauma screening for children and youth in foster care

### Michael D. Pullmann^1^, Barbara Lucenko^2^, Bridget Pavelle^2^, Jacqueline A. Uomoto^1^, Andrea Negrete^1^, Molly Cevasco^1^, Suzanne E. U. Kerns^1^

#### ^1^Department of Psychiatry and Behavioral Sciences, University of Washington, Seattle, WA, 98109, USA; ^2^Washington State Department of Social and Health Services Research and Data Analysis Division, Olympia, WA, 98504, USA

##### **Correspondence:** Michael D. Pullmann (pullmann@uw.edu) – Department of Psychiatry and Behavioral Sciences, University of Washington, Seattle, WA, 98109, USA

**Background**

Effective statewide implementation of approaches to identifying and treating youth in foster care with behavioral health needs requires monitoring of process and outcomes. Administrative data provide high-quality and efficient alternatives for describing populations served across systems.

**Materials and methods**

Washington State maintains integrated client data across multiple service systems, including child welfare, physical and behavioral health, and juvenile justice. This infrastructure was leveraged in an evaluation of a behavioral health and trauma symptom screening and referral protocol for youth entering foster care.

**Results**

There is a high degree of overlap among service needs for foster youth. From 3-17 years of age, they are 3.5-4 times more likely to have behavioral health treatment needs than youth with Medicaid coverage who are not in foster care. Over half (62 %) of youth entering foster care were recommended for treatment after screening. Of those recommended for treatment based on screening, 57 % received behavioral health services within six months, compared to 33 % of those who were not recommended for treatment. Many youth appeared resilient, with 37 % scoring below cutoff on all behavioral health measures at intake and six months later.

**Conclusions**

Linking administrative datasets is useful for evaluation, especially for populations with cross-system service needs. Based on these findings, universal screening for mental, emotional, and behavioral problems in foster care is both feasible and useful, but screening is resource intensive and results are not absolute. In our analysis, screening positive increased the likelihood of receiving services but did not guarantee service receipt.

**Acknowledgements**

Funded by the US Department of Health and Human Services, Administration for Children and Families, Children’s Bureau, Grant #90-C01103

## A15 Intermediary organizations as a vehicle to promote efficiency and speed of implementation

### Robert P. Franks, Christopher Bory

#### Judge Baker Children's Center, Boston, MA, 02120, USA

##### **Correspondence:** Robert P. Franks (rfranks@jbcc.harvard.edu) – Judge Baker Children's Center, Boston, MA, 02120, USA

**Background**

Intermediary organizations can promote efficiency and speed of implementation through the Active Implementation Framework as defined by the National Implementation Research Network (NIRN). Intermediaries have been identified by Franks and Bory as integral to the implementation of evidence-based practices. Research has supported seven common roles and activities that characterize the work of intermediaries including: 1) consultation activities, 2) best practice model development, 3) purveyors of evidence-based practices, 4) quality assurance, 5) outcome evaluation, 6) training, public awareness and education and 7) policy and systems development. These roles and activities can support the active implementation process.

**Materials and methods**

This presentation utilized case examples from a survey of 68 intermediary organizations conducted by the authors to illustrate their role in the active implementation process.

**Results**

Intermediaries support active implementation in several key ways. Intermediaries can help select effective interventions and co-create capacity by: creating well-defined implementation teams, structuring implementation methods, and helping to create and facilitate enabling contexts that result in socially significant outcomes. Specifically, intermediaries play a critical role by structuring and driving the change process and developing and using tools to support implementation. Intermediaries facilitate competency, organizational, and leadership drivers through structured implementation approaches and engagement of key stakeholders.

Intermediaries also promote fidelity and sustainability through quality improvement activities.

**Conclusions**

Intermediaries can play a significant role in supporting the active implementation process. By driving the efficiency and speed of implementation and by acting as a facilitator of the active implementation process intermediaries contribute to positive social outcomes and sustained practice change.

## A16 Applying the Consolidated Framework for Implementation Research constructs directly to qualitative data: The power of implementation science in action

### Edward J. Miech^1,2,3,4,5^, Teresa M. Damush^1,2,3,4^

#### ^1^Department of Veterans Affairs (VA) Health Services Research and Development (HSR&D) PRIS-M Quality Enhancement Research Initiative (QUERI), Indianapolis, Indiana, 46202, USA; ^2^VA HSR&D Center for Health Information and Communication (CHIC), Richard L. Roudebush VA Medical Center, Indianapolis, Indiana, 46202, USA; ^3^Department of Internal Medicine, Indiana University School of Medicine, Indianapolis, Indiana, 46202, USA; ^4^Regenstrief Institute, Indianapolis, Indiana, 46202, USA; ^5^Department of Emergency Medicine, Indiana University School of Medicine, Indianapolis, Indiana, 46202, USA

##### **Correspondence:** Edward J. Miech (edward.miech@va.gov) – Department of Emergency Medicine, Indiana University School of Medicine, Indianapolis, Indiana, 46202, USA

**Background**

An innovative new analytic strategy in implementation science is the direct application of constructs from the Consolidated Framework for Implementation Research (CFIR) [1] to qualitative data, including the assigning of valence (i.e., positive or negative) and magnitude (i.e., weak or strong) to individual CFIR constructs and then using these ratings to analyze the association of CFIR constructs with implementation outcomes.

**Materials and methods**

An eight-person study team based in Indianapolis undertook the task of systematically rating 33 site visits (representing over 300 transcribed interviews) with 20 CFIR constructs for valence and magnitude as part of the VA-funded Rich-context Evaluation of INSPIRE (RE-INSPIRE) project in 2014-15. The project held weekly, in-person, two-hour meetings to assign facility-level ratings for each site visit. During meetings the team followed a structured discussion format and then assigned ratings using a real-time, digital secret ballot in the form of an Audience Response System. Ratings were not final until unanimity had been reached; if the team did not reach agreement the first time, further discussion ensued followed by additional votes. All team meetings were audiorecorded and transcribed.

**Results**

Over 650 CFIR ratings were assigned one-by-one through this team approach.

**Conclusions**

RE-INSPIRE is the largest study to date to apply CFIR constructs directly to qualitative data and the first to assign CFIR ratings as a team. The study pioneered the use of an Audience Response System to harness the individual expertise of team members yet adhere to a standard of team consensus. This project contributed new methods to implementation science for systematically assigning CFIR ratings.

**Reference**

1. Damschroder LJ, Aron DC, Keith RE, Kirsh SR, Alexander JA, Lowery JC. Fostering implementation of health services research findings into practice: a consolidated framework for advancing implementation science. Implement Sci 2009; 4:50.

## A17 Efficient and effective scaling-up, screening, brief interventions, and referrals to treatment (SBIRT) training: a snowball implementation model

### Jason Satterfield^1^, Derek Satre^2^, Maria Wamsley^1^, Patrick Yuan^1^, Patricia O’Sullivan^1^

#### ^1^Department of Medicine, University of California, San Francisco, CA, USA; ^2^Department of Psychiatry, University of California, San Francisco, CA, USA

##### **Correspondence:** Jason Satterfield (jason.satterfield@ucsf.edu) – Department of Medicine, University of California, San Francisco, CA, USA

**Background**

Medical clinics often “re-invent the wheel” when promoting new evidence-based behavioral practices rather than building on the gains of prior implementation efforts. Our purpose was to determine if a “snowball implementation” model with near-peer consultations and community partnerships could be an effective approach for efficiently scaling-up screening, brief interventions, and referrals to treatment (SBIRT) skills training programs across diverse settings [1].

**Materials and methods**

We conducted a five-year case study of “snowball implementation” involving five medical residency training programs interested in implementing SBIRT for substance use disorders into their clinical practices. Each year, one program implemented SBIRT training using materials and processes developed by the prior year’s program. Qualitative interviews of key informants and review of program materials assessed important implementation processes and outcomes drawn from the Consolidated Framework for Implementation Research (CFIR) [2].

**Results**

All programs successfully implemented SBIRT training and systems adaptations. Early programs invested more time and resources in developing materials and processes but each program “handed off” products and lessons learned to subsequent programs. Internal champions effectively used near-peer consultations, enabling them to design more effective and efficient program-specific implementations to successfully train residents in SBIRT.

**Conclusions**

By creating a near-peer community, programs evolved successful program-specific implementations, gleaning lessons from each other. This model could inform others regarding how to build implementations collaboratively rather than relying solely on individual or local strategies.

**References**

1. Chamberlain P, Roberts R, Jones H, Marsenich L, Sosna T, Price JM. Three collaborative models for scaling up evidence-based practices. Adm Policy Ment Health 2012; 39:278– 290.

2. Damschroder LJ, Aron DC, Keith RE, Kirsh SR, Alexander JA, Lowery JC. Fostering implementation of health services research findings into practice: A consolidated framework for advancing implementation science. Implement Sci. 2009; 4:50.

## A18 Matching models of implementation to system needs and capacities: addressing the human factor

### Helen Best^1^, Susan Velasquez^2^

#### ^1^Treatment Implementation Collaborative, LLC, Seattle, WA, 98136, USA; ^2^Department of State Hospitals, Sacramento, CA, 95814, USA

##### **Correspondence:** Helen Best (hbest@ticllc.org) – Treatment Implementation Collaborative, LLC, Seattle, WA, 98136, USA

**Introduction**

While Dialectical Behavior Therapy (DBT) has been widely disseminated, most of the large scale system initiatives have faced formidable obstacles which make the implementation extremely challenging.

**Materials and methods**

In a large scale installation of DBT in the California State Hospital System, six pillars impacting implementation and sustainment that are interdependent and are often incongruent across time have been defined.

**Results**

The need for an overarching plan addressing the fit of the treatment, funding, administrative and clinical support, all supported by high quality training, consultation and supervision is well documented and in play within this implementation of DBT. Yet the six levels of support required to move from planning to outcomes requires constant and ongoing tending. These areas are: central office (DSH), hospital level executive administration, discipline silo’s, units implementing DBT, clinicians learning to provide DBT, and system flow (patient fit, beds, mandates, incidents, etc.).

**Conclusions**

The implications of the present work illustrate the impact of Good, Cheap and Fast against the backdrop of time, funding and scalability to discuss how these hierarchical layers play critical roles across day-to-day implementation of an EBP. Installation can be achieved. Sustainability is most impacted by the human factor as decisions roll across all levels and impact day-to-day treatment outcomes and endurance. There is a critical need to address ongoing development of implementation champions and/or teams across all systemic levels, highlighted through learning from Napa State Hospital in particular.

## A19 Agency characteristics that facilitate efficient and successful implementation efforts

### Miya Barnett^1^, Lauren Brookman-Frazee^2,3^, Jennifer Regan^1^, Nicole Stadnick^2,3^, Alison Hamilton^4^, Anna Lau^1^

#### ^1^Department of Psychology, University of California, Los Angeles, Los Angeles, CA, 90095, USA; ^2^Department of Psychiatry, University of California, San Diego, La Jolla, CA, 92093, USA; ^3^Child and Adolescent Services Research Center, San Diego, CA, 92123, USA; ^4^Department of Psychiatry and Biobehavioral Sciences, David Geffen School of Medicine, University of California, Los Angeles, Los Angeles, CA, 90095, USA

##### **Correspondence:** Miya Barnett (miyabarnett@psych.ucla.edu) – Department of Psychology, University of California, Los Angeles, Los Angeles, CA, 90095, USA

**Background**

To tailor implementation strategies to community needs, it is important to understand how agency characteristics impact evidence-based practice uptake. A system reform in Los Angeles County fiscally mandated use of specific practices. County representatives conducted agency site visits to document early implementation efforts. Based on Aarons et al.’s theory on the effect of inner context factors on implementation, it was hypothesized that agency size and client demographics would impact implementation experiences [1].

**Materials and methods**

A mixed-methods design integrated claims data and site visit narratives from 98 agencies to identify agency characteristics associated with implementation experiences. Qualitative analyses used coding consensus, co-occurrence, and comparison methodology to extract themes [2].

Agencies were characterized by: 1) size, with agencies serving <100 clients classified as small (*N* = 27), 100-500 as moderate (*N* = 45), and >500 as large (*N* = 26) and 2) having a higher proportion of Spanish-speaking clients (>20 %; *N* = 46).

**Results**

Agency size and proportion of Spanish-speaking clients were associated with implementation experiences. Specifically, large- and moderate-sized agencies described more innovative changes to infrastructure (e.g., utilizing technology to monitor implementation, identifying practice champions). Small agencies experienced more challenges (e.g., staff turnover). Agencies that served a higher proportion of Spanish-speaking clients needed to adapt practices, including translating materials and focusing on client engagement.

**Conclusions**

Structural agency characteristics should be considered when tailoring implementation strategies for community settings. Smaller agencies may benefit from support related to maintaining trained staff. Agencies that serve diverse clientele may benefit from support adapting and translating practices.

**References**

1. Aarons GA, Hurlburt M, Horwitz SM. Advancing a conceptual model of evidence-based practice implementation in public service sectors. Admn Policy Ment Health. 2011 Jan 1;38(1):4-23.

2. Willms DG, Best JA, Taylor DW, Gilbert JR, Wilson D, Lindsay EA, Singer J. A systematic approach for using qualitative methods in primary prevention research. Med Anthropol Q. 1990 Dec 1;4(4):391-409.

## A20 Rapid assessment process: Application to the Prevention and Early Intervention transformation in Los Angeles County

### Jennifer Regan^1^, Alison Hamilton^2^, Nicole Stadnick^3,4^, Miya Barnett^1^, Anna Lau^1^, Lauren Brookman-Frazee^3,4^

#### ^1^Department of Psychology, University of California, Los Angeles, Los Angeles, CA, 90095, USA; ^2^Department of Psychiatry and Biobehavioral Sciences, David Geffen School of Medicine, University of California, Los Angeles, CA, 90095, USA; ^3^Department of Psychiatry, University of California, San Diego, La Jolla, CA, 92093, USA; ^4^Child and Adolescent Services Research Center, San Diego, CA, 92123, USA

##### **Correspondence:** Jennifer Regan (jregan@psych.ucla.edu) – Department of Psychology, University of California, Los Angeles, Los Angeles, CA, 90095, USA

**Background**

The rapid assessment process (RAP) represents an efficient qualitative analytical method used to develop a preliminary understanding of complicated situations. This study illustrates multiple applications of RAP within a mixed-methods study examining the sustainment of evidence-based practices following a mental health system transformation in the Los Angeles County Department of Mental Health (LACDMH).

**Materials and methods**

First, RAP was applied to LACDMH site visit documents from early in the transformation to characterize implementation experiences. The second application used RAP to assess the utility of semi-structured interviews regarding therapist adaptations to specific practices. RAP procedures included: identifying consistent domain names for data units (e.g., interview prompts), developing a template for summarizing domains, applying summary templates to data, and creating a matrix to contrast domains by variables of interest (e.g., informant type, site).

**Results**

In Study 1, RAP identified emergent themes regarding early implementation experiences (e.g., infrastructure development to facilitate implementation and investments in training) and generated hypotheses that experiences differed by agency characteristics (e.g., size, decentralization). In Study 2, RAP confirmed that the interview protocol generated information for most questions but follow-up questions were needed to elicit detail about the nature of and motivation behind therapist adaptations.

**Conclusions**

RAP findings served valuable and distinct purposes in each application, identifying directions for further analysis in the early implementation documents and improving the clarity and design of interview guides. Overall, RAP is well-suited for projects that require quick integration, interpretation, and synthesis of data and can identify unique patterns through the simultaneous viewing of large quantities of data.

## A21 The development of the Evidence-Based Practice-Concordant Care Assessment: An assessment tool to examine treatment strategies across practices

### Nicole Stadnick^1,2^, Anna Lau^3^, Miya Barnett^3^, Jennifer Regan^3^, Scott Roesch^4^, Lauren Brookman-Frazee^1,2^

#### ^1^Department of Psychiatry, University of California, San Diego, La Jolla, CA, 92093, USA; ^2^Child and Adolescent Services Research Center, San Diego, CA 92123, USA; ^3^Department of Psychology, University of California, Los Angeles, Los Angeles, CA, 90095, USA; ^4^Department of Psychology, San Diego State University, San Diego, CA, 92182, USA

##### **Correspondence:** Nicole Stadnick (nstadnic@ucsd.edu) – Child and Adolescent Services Research Center, San Diego, CA 92123, USA

**Background**

Measuring the delivery of essential EBP (evidence-based practice) components is important to improve EBP implementation in mental health (MH) settings. Existing fidelity monitoring methods are labor-intensive and practice-specific. Efficient measures with flexible use across EBPs are needed in community settings. Consequently, the EBP Concordant Care Assessment (ECCA) is being developed to assess the extent to which community providers deliver strategies considered essential across child MH targets. This ECCA iteration was developed to include strategies across six practices implemented in a public children’s MH system: Cognitive Behavioral Interventions for Trauma in Schools, Child-Parent Psychotherapy, Managing and Adapting Practices, Seeking Safety, Trauma-Focused Cognitive Behavior Therapy, and Triple P.

**Materials and methods**

First, practice inventories and training materials were reviewed to inform strategy selection. Next, an adapted Delphi method was used to identify strategies considered essential by 22 practice experts (intervention developers or master trainers) who completed a survey in which they rated 63 strategies from (-3) absolutely interfering to (3) absolutely essential.

**Results**

Strategies were retained based on expert agreement on ratings of essential (1 to 3) or interfering (-1 to -3) (e.g., Psychoeducation, Exposure). Strategies were subsequently grouped into six “practice families” based on shared MH target (e.g., Trauma, Conduct). After content analysis to improve clarity, the final number of strategies was 54 (*M* strategies per family = 21).

**Conclusions**

The ECCA represents an efficient tool to assess delivery of multiple practices in community MH. Subsequent validation of therapist-reported ECCA with observational coding offers promise in facilitating large-scale EBP implementation in children’s MH.

## A22 Refining a compilation of discrete implementation strategies and determining their importance and feasibility

### Byron J. Powell^1^, Thomas J. Waltz^2,3^, Matthew J. Chinman^4^, Laura Damschroder^5^, Jeffrey L. Smith^6,7^, Monica M. Matthieu^7,8^, Enola K. Proctor^9^, JoAnn E. Kirchner^6,7,10^

#### ^1^Department of Health Policy and Management, Gillings School of Global Public Health, University of North Carolina at Chapel Hill, Chapel Hill, NC, 27599, USA; ^2^Department of Psychology, Eastern Michigan University, Ypsilanti, MI, 48197, USA; ^3^Veterans Affairs (VA) Center for Clinical Management Research, Ann Arbor, MI, 48105, USA; ^4^RAND, Pittsburgh, PA, 15213, USA; ^5^VA Quality Enhancement Research Initiative (QUERI) for Personalizing Options through Veteran Engagement (PROVE), Ann Arbor, MI, 48105, USA; ^6^VA Quality Enhancement Research Initiative (QUERI) for Team-Based Behavioral Health, North Little Rock, AR, 72114, USA; ^7^Central Arkansas Veterans Healthcare System, North Little Rock, AR, 72114, USA; ^8^School of Social Work, Saint Louis University, St. Louis, MO, 63104, USA; ^9^Brown School, Washington University in St. Louis, St. Louis, MO, 63130, USA; ^10^Department of Psychiatry, University of Arkansas for Medical Sciences, Little Rock, AR 72205, USA

##### **Correspondence:** Byron J. Powell (bjpowell@unc.edu) – Department of Health Policy and Management, Gillings School of Global Public Health, University of North Carolina at Chapel Hill, Chapel Hill, NC, 27599, USA

**Background**

Identifying feasible and effective implementation strategies remains a significant challenge. This is partly due to a lack of conceptual clarity in the field, and insufficient guidance about how to select appropriate strategies. The Expert Recommendations for Implementing Change (ERIC) project [1] aimed to: 1) establish expert consensus on implementation strategy terms, definitions, and categories, and 2) develop recommendations for strategies likely to be effective in integrating EBPs into VA mental health service settings. This abstract reports methods and findings from Aim 1.

**Materials and methods**

Purposive sampling was used to recruit a panel of implementation science and clinical experts (*N* = 71). The expert panel was engaged in a three-round modified Delphi process to generate consensus on strategies and definitions. Rounds 1 and 2 involved web-based surveys that prompted edits and additions to the strategy terms and definitions from Powell et al. [2]. The third round involved a live, web-based polling and consensus process. Experts were subsequently engaged in a concept mapping process to organize implementation strategies into conceptually distinct categories and to derive ratings of the importance and feasibility.

**Results**

The three-round modified Delphi process yielded a final compilation of 73 discrete implementation strategies and definitions [3]. The concept mapping process yielded nine distinct clusters, as well as feasibility and importance ratings for both individual discrete strategies and for broad categories of strategies [4].

**Conclusions**

The refined compilation [3] and ratings of feasibility and importance [4] can be used to build multi-faceted, multi-level implementation strategies for implementation research and practice.

**Acknowledgments**

This study was funded by the Department of Veterans Affairs (VA), Mental Health Quality Enhancement Research Initiative (MH QUERI). The results described are based on data analyzed by the authors and do not represent the views of the VA, Veterans Health Administration (VHA), or the United States Government.

**References**

1. Waltz TJ, Powell BJ, Chinman MJ, Smith JL, Matthieu MM, Proctor EK, Damschroder LJ, Kirchner JE. Expert recommendations for implementing change (ERIC): Protocol for a mixed methods study. Implement Sci. 2014; 9:1–12.

2. Powell BJ, McMillen JC, Proctor EK, Carpenter CR, Griffey RT, Bunger AC, Glass JE, York JL. A compilation of strategies for implementing clinical innovations in health and mental health. Med Care Res Rev. 2012; 69:123–157.

3. Powell BJ, Waltz TJ, Chinman MJ, Damschroder LJ, Smith JL, Matthieu MM, Proctor EK, Kirchner JE. A refined compilation of implementation strategies: Results from the Expert Recommendations for Implementing Change (ERIC) project. Implement Sci. 2015; 10:1–14.

4. Waltz TJ, Powell BJ, Matthieu MM, Damschroder LJ, Chinman MJ, Smith JL, Proctor EK, Kirchner JE. Use of concept mapping to characterize relationships among implementation strategies and assess their feasibility and importance: Results from the Expert Recommendations for Implementing Change (ERIC) study. Implement Sci. 2015; 10:1–8.

## A23 Structuring complex recommendations: Methods and general findings

### Thomas J. Waltz^1,2^, Byron J. Powell^3^, Matthew J. Chinman^4^, Laura J. Damschroder^2,5^, Jeffrey L. Smith^6,7^, Monica J. Matthieu^8,9,7^, Enola K. Proctor^10^, JoAnn E. Kirchner^6,7,11^

#### ^1^Department of Psychology, Eastern Michigan University, Ypsilanti, MI, 48197, USA; ^2^Veterans Affairs (VA) A Center for Clinical Management Research, Ann Arbor, MI, 48105, USA; ^3^Department of Health Policy and Management, University of North Carolina at Chapel Hill, Chapel Hill, NC, 27599, USA; ^4^RAND, Pittsburgh, PA, 15213, USA; ^5^VA Quality Enhancement Research Initiative (QUERI) for PeRsonalizing Options through Veteran Engagement (PROVE), Ann Arbor, MI, 48105, USA; ^6^VA Quality Enhancement Research Initiative (QUERI) for Team-Based Behavioral Health, North Little Rock, AR, 72114, USA; ^7^Central Arkansas Veterans Healthcare System, North Little Rock, AR, 72114, USA; ^8^School of Social Work, Saint Louis University, St. Louis, MO, 63103, USA; ^9^Central Arkansas Health Care System, North Little Rock, AR, 72114, USA; ^10^Brown School, Washington University in St. Louis, St. Louis, MO, 63130, USA; ^11^Department of Psychiatry, University of Arkansas for Medical Sciences, Little Rock, AR, 72205, USA

##### **Correspondence:** Thomas J. Waltz (twaltz1@emich.edu) – Veterans Affairs (VA) A Center for Clinical Management Research, Ann Arbor, MI, 48105, USA

**Background**

In the absence of an adequate evidence base for constructing multiple element implementation supports for practice initiatives, it is desirable to have a structured process for obtaining expert recommendations. The Expert Recommendations for Implementing Change (ERIC) project [1] had as its second aim to obtain such recommendations for supporting three high priority Veterans Health Administration mental health practices. Earlier phases of the ERIC project provided the foundation for the strategies included in the recommendation process [2,3].

**Materials and methods**

Menu-based choice (MBC) tasks were used to provide a highly structured environment for making complex recommendations. Participants were provided with descriptions of the practice changes and hypothetical Veterans Affairs (VA) practice settings. Structured worksheets for the MBC task were used to facilitate the building multiple strategy implementation approaches.

Experts indicated how essential each of 73 implementation strategies were for each practice change.

**Results**

The reported results focused on strategies for which there was majority consensus (≥50 %) ratings as absolutely essential or absolutely inessential. Twenty-seven strategies received majority consensus as being absolutely essential for one or more of the practice changes. Seven of the absolutely essential strategies applied to all three practice changes. Fourteen strategies received majority consensus as being absolutely inessential for any of the practice changes.

**Conclusions**

The MBC method produced unique recommendations for the practices included in this study. The variations in the recommendations were consistent with the needs of these different practices. These results suggest that MBC is a promising tool for obtaining and characterizing expert consensus when planning implementation initiatives.

**Acknowledgments**

This study was funded by the Department of Veterans Affairs (VA), Mental Health Quality Enhancement Research Initiative (MH QUERI). The results described are based on data analyzed by the authors and do not represent the views of the VA, Veterans Health Administration (VHA), or the United States Government.

**References**

1. Waltz TJ, Powell BJ, Chinman MJ, Smith JL, Matthieu MM, Proctor EK, Damschroder LJ, Kirchner JE. Expert recommendations for implementing change (ERIC): Protocol for a mixed methods study. Implement Sci. 2014 Mar 26;9:1-2.

2. Powell BJ, Waltz TJ, Chinman MJ, Damschroder LJ, Smith JL, Matthieu MM, Proctor EK, Kirchner JE. A refined compilation of implementation strategies: Results from the Expert Recommendations for Implementing Change (ERIC) project. Implement Sci. 2015;10:1–14.

3. Waltz TJ, Powell BJ, Matthieu MM, Damschroder LJ, Chinman MJ, Smith JL, Proctor EK, Kirchner JE. Use of concept mapping to characterize relationships among implementation strategies and assess their feasibility and importance: Results from the Expert Recommendations for Implementing Change (ERIC) study. Implement Sci. 2015;10:1–8.

## A24 Implementing prolonged exposure for post-traumatic stress disorder in the Department of Veterans Affairs: Expert recommendations from the Expert Recommendations for Implementing Change (ERIC) project

### Monica M. Matthieu^1,2^, Craig S. Rosen^3^, Thomas J. Waltz^4,5^, Byron J. Powell^6^, Matthew J. Chinman^7^, Laura J. Damschroder^5,8^, Jeffrey L. Smith^9^, Enola K. Proctor^10^, JoAnn E. Kirchner^9,11^

#### ^1^School of Social Work, Saint Louis University, St. Louis, MO, 63103, USA; ^2^Central Arkansas Health Care System, North Little Rock, AR, 72114, USA; ^3^Dissemination & Training Division National Center for Posttraumatic Stress Disorder (PTSD), Veterans Affairs (VA) Palo Alto Health Care System, Menlo Park, CA, 94025, USA; ^4^Department of Psychology, Eastern Michigan University, Ypsilanti, MI 48197, USA; ^5^VA Center for Clinical Management Research, Ann Arbor, MI 48105, USA; ^6^Department of Health Policy and Management, University of North Carolina at Chapel Hill, Chapel Hill, NC 27599, USA; ^7^RAND, Pittsburgh, PA, 15213, USA; ^8^VA Center for Clinical Management Research and VA Quality Enhancement Research Initiative (QUERI) for PeRsonalizing Options through Veteran Engagement (PROVE), Ann Arbor, MI, 48105, USA; ^9^VA Quality Enhancement Research Initiative (QUERI) for Team-Based Behavioral Health, Central Arkansas Veterans Healthcare System, North Little Rock, AR, 72114, USA; ^10^Brown School, Washington University in St. Louis, St. Louis, MO, 63130, USA; ^11^Department of Psychiatry, University of Arkansas for Medical Sciences, Little Rock, AR, 72205, USA

##### **Correspondence:** Monica M. Matthieu (mmatthie@slu.edu) – Central Arkansas Health Care System, North Little Rock, AR, 72114, USA

**Background**

The Expert Recommendations for Implementing Change (ERIC) project [1] utilized rigorous methods to support a highly structured and transparent recommendation process that actively engaged key stakeholders throughout the project’s execution. This abstract describes the ERIC recommendations for implementation of one evidence based psychotherapy for treating post-traumatic stress disorder (PTSD) among veterans in the Veterans Health Administration (VHA).

**Materials and methods**

The ERIC project purposively recruited a panel of implementation science and clinical experts (*N* = 71) who participated in consensus building activities using existing definitions [2] to generate an expanded compilation of strategies [3]. Stakeholders (*N* = 22) affiliated with the National Center for PTSD (NC-PTSD) engaged in an iterative process of evaluating strategies utilized to implement prolonged exposure (PE) for PTSD, then compared and contrasted the strategies actually used with highly structured expert recommendations obtained from the ERIC project.

**Results**

Two strategies deemed “absolutely essential” based on ratings from the ERIC project were not endorsed by stakeholders: 1) conduct local needs assessment and 2) develop a formal implementation blueprint, likely because of the national dissemination model used for PE, which did not include these strategies. Conversely, all five strategies deemed “absolutely inessential” in the ERIC project were also not endorsed by stakeholders.

**Conclusions**

This confirmatory review of the ERIC recommendation results versus the actual implementation strategies used to implement PE for PTSD in VHA healthcare settings offers support for the use of structured recommendation methods to aid in the selection of implementation strategies.

**Acknowledgments**

This study was funded by the Department of Veterans Affairs (VA), Mental Health Quality Enhancement Research Initiative (MH QUERI). The results described are based on data analyzed by the authors and do not represent the views of the VA, Veterans Health Administration (VHA), or the United States Government.

**References**

1. Waltz TJ, Powell BJ, Chinman MJ, Smith JL, Matthieu MM, Proctor EK, Damschroder LJ, Kirchner JE. Expert recommendations for implementing change (ERIC): Protocol for a mixed methods study. Implement Sci. 2014;9:1–12.

2. Powell BJ, McMillen JC, Proctor EK, Carpenter CR, Griffey RT, Bunger AC, Glass JE, York JL. A compilation of strategies for implementing clinical innovations in health and mental health. Med Care Res Rev. 2012;69:123–157.

3. Powell BJ, Waltz TJ, Chinman MJ, Damschroder LJ, Smith JL, Matthieu MM, Proctor EK, Kirchner JE. A refined compilation of implementation strategies: Results from the Expert Recommendations for Implementing Change (ERIC) project. Implement Sci. 2015;10:1–14.

4. Waltz TJ, Powell BJ, Matthieu MM, Damschroder LJ, Chinman MJ, Smith JL, Proctor EK, Kirchner JE. Use of concept mapping to characterize relationships among implementation strategies and assess their feasibility and importance: Results from the Expert Recommendations for Implementing Change (ERIC) study. Implement Sci. 2015;10:1–8.

## A25 When readiness is a luxury: Co-designing a risk assessment and quality assurance process with violence prevention frontline workers in Seattle, WA

### Sarah C. Walker^1^, Asia S. Bishop^1^, Mariko Lockhart^2^

#### ^1^Department of Psychiatry and Behavioral Sciences, University of Washington, Seattle, WA, 98102, USA; ^2^Seattle Youth Violence Prevention Initiative, Department of Education and Early Learning, City of Seattle, Seattle, WA, 98104, USA

##### **Correspondence:** Sarah C. Walker (secwalkr@uw.edu) – Department of Psychiatry and Behavioral Sciences, University of Washington, Seattle, WA, 98102, USA

**Background**

The current study examined the feasibility of implementing a standardized violence risk assessment across multiple, independent service providers in the Seattle Youth Violence Prevention Initiative (SYVPI) who have not traditionally used actuarial tools to guide case management decisions.

**Materials and methods**

Twenty-eight community providers serving the initiative were interviewed in individual and group sessions using a semi-structured format. The respondents were case managers, intake and referral specialists and street outreach workers. The interviews focused on perceptions of a tool currently in use, its relevance to practice, as well as consistency in administration and interpretation.

**Results**

Results showed that without clear relevance to practice, risk assessment tools are unlikely to be used consistently or effectively in community-based prevention settings. Specific recommendations from the respondents included 1) shortening the tool; 2) reorganizing questions so sensitive items are asked later in the interview; 3) developing clear guidelines for how to translate results into case management plans and 4) developing a quality assurance infrastructure. Consequently, the original tool was modified to meet the needs of providers by addressing these issues.

**Conclusions**

This project illustrates the feasibility and benefits of a co-design process as an alternative to implementing previously developed products in new settings to encourage buy in among practitioners. The results suggest that risk and needs assessment tools for community agencies largely focused on youth development should be relatively brief, oriented towards case planning and have a quality assurance infrastructure.

## A26 Implementation potential of structured recidivism risk assessments with justice-involved veterans: Qualitative perspectives from providers

### Allison L. Rodriguez^1^, Luisa Manfredi^2^, Andrea Nevedal^2^, Joel Rosenthal^3^, Daniel M. Blonigen^2,4^

#### ^1^National Center for Posttraumatic Stress Disorder (PTSD), Veterans Affairs (VA) Palo Alto Health Care System, Menlo Park, CA, 94025, USA; ^2^Health Services Research & Development (HSR&D) Center for Innovation to Implementation, Department of Veterans Affairs, Palo Alto Health Care System, Menlo Park, CA, 94025, USA; ^3^Veterans Health Administration (VHA) Veterans Justice Programs, Department of Veterans Affairs, Washington D.C., 20002, USA; ^4^Department of Clinical Psychology, Palo Alto University, Palo Alto, CA, 94304, USA

##### **Correspondence:** Allison L. Rodriguez (allison.rodriguez@va.gov) – National Center for Posttraumatic Stress Disorder (PTSD), Veterans Affairs (VA) Palo Alto Health Care System, Menlo Park, CA, 94025, USA

**Background**

Utilization of structured assessments to evaluate justice-involved individuals’ recidivism risk is central to the Risk-Need-Responsivity model [1] of offender rehabilitation. The Veterans Health Administration’s (VHA) Veterans Justice Programs (VJP) specialists serve as first-line responders to justice-involved veterans in the reentry process, and aim to link clients with appropriate services, potentially reducing recidivism risk. Little is known about the perception of structured risk assessments (SRAs) or the possibility of implementing them within VHA. This study aimed to assess specialists’ perceptions of SRA helpfulness, as well as perceived barriers and facilitators to implementation.

**Materials and methods**

Qualitative semi-structured interviews were conducted with 63 randomly selected VJP specialists across the VHA, and standard content coding and pile sorting methods were used to identify themes. See Blonigen et al. [2] for more information.

**Results**

Few specialists use SRAs; however, most (70 %) indicated that they would be helpful. Themes of helpfulness included: triage and case management, facilitation of communication regarding clients, reductions in risk of adverse contact between low and high risk clients, provision of direct feedback to clients, and use of data to support quality improvement initiatives. Themes of potential barriers to implementation included: lack of time and resources, reliability concerns, scores oversimplifying client needs, scores discouraging treatment for riskier clients, and documentation concerns. Themes of potential facilitators included: leadership support and provision of education, training, and resources.

**Conclusions**

Findings call for the consideration of implementing SRAs at VHA to optimize care for justice-involved veterans. Qualitative themes offer insight into expected barriers and facilitators of such efforts.

**Acknowledgements**

This work was supported by the Department of Veterans Affairs (VA), Health Services Research & Development (HSR&D)/Quality Enhancement Research Initiative (QUERI) (RRP 12-507) awarded to the last author. The results described are based on data analyzed by the authors and does not represent the views of the VA, Veterans Health Administration (VHA), or the United States Government.

**References**

1. Andrews DA, Bonta J, Hoge RD. Classification for effective rehabilitation: Rediscovering psychology. Crim Justice Behav. 1990;17:19-52.

2. Blonigen DM, Rodriguez AL, Manfredi L, Britt J, Nevedal A, Finlay AK, Rosenthal J, Smelson D, Timko C. The availability and utility of services to address risk factors for recidivism among justice-involved veterans. Crim Justice Policy Rev. 2016

## A27 Developing empirically informed readiness measures for providers and agencies for the Family Check-Up using a mixed methods approach

### Anne M. Mauricio^1^, Thomas D. Dishion^1^, Jenna Rudo-Stern^1^, Justin D. Smith^2^

#### ^1^Department of Psychology, Resource for Advancing Children’s Health (REACH) Institute, Arizona State University, Tempe, AZ, 85287, USA; ^2^Department of Psychiatry and Behavioral Sciences, Feinberg School of Medicine, Northwestern University, Chicago, IL, 60657, USA

##### **Correspondence:** Anne M. Mauricio (anne.mauricio@asu.edu) – Department of Psychology, Resource for Advancing Children’s Health (REACH) Institute, Arizona State University, Tempe, AZ, 85287, USA

**Background**

This study explored the utility and validity of the Family Check-Up (FCU) Provider Readiness Assessment (PRA) to inform FCU provider selection. The FCU is a brief, assessment-driven, intervention that improves child problem behaviors [1,2,3]. The PRA is a survey that assesses provider attributes linked with uptake of evidence-based interventions (EBIs; e.g., attitudes about EBIs) [4] and acceptability of the FCU specifically (e.g., assessment-driven). A secondary goal of this study was to understand early-adopting provider’s perspectives about what facilitates FCU uptake in the domains of provider selection, training, and consultation [5].

**Materials and methods**

Fifteen early-adopting FCU providers representing publicly funded behavioral health agencies completed the PRA. They subsequently participated in focus groups (*N* = 3) to discuss the PRA’s validity and utility as a tool to select FCU providers and to explore facilitators of FCU uptake. We transcribed focus group data and conducted a thematic content analysis [6].

**Results**

Thematic results suggested the PRA is useful and valid but that administrators should not rely solely on the PRA and discount provider choice because top-down mandates to train in EBIs decrease buy-in. Consultation and training-related facilitators included adapting the consultation model so it can embed within standard supervisory practices and training providers to self-assess fidelity to decrease resistance to implementation monitoring.

**Conclusions**

The PRA is a useful tool to select providers with high readiness to adopt the FCU, if balanced with respect for providers’ professional autonomy. The perspectives of early-adopting providers can inform indices of agency readiness and help build capacity for FCU uptake.

**References**

1. Dishion TJ, Brennan LM, Shaw DS, McEachern AD, Wilson MN, Booil J. Prevention of problem behavior through annual family check-ups in early childhood: intervention effects from home to early elementary school. J Abnorm Child Psychol. 2014;42(3):343- 54.

2. Shaw DS, Dishion TJ, Supplee L, Gardner F, Arnds K. Randomized trial of a family- centered approach to the prevention of early conduct problems: 2-year effects of the family check-up in early childhood. J Consult Clin Psychol. 2006;74(1):1.

3. Smith JD, Stormshak EA, Kavanagh K. Results of a pragmatic effectiveness- implementation hybrid trial of the Family Check-Up in community mental health agencies. Adm Policy Ment Health. 2015;42(3):265-78.

4. Chaudoir SR, Dugan AG, Barr C. Measuring factors affecting implementation of health innovations: A systematic review of structural, organizational, provider, patient, and innovation level measures. Implement Sci. 2013;8(1):22.

5. Fixsen DL, Blase KA, Naoom SF, Wallace F. Core implementation components. Res Social Work Prac. 2009;19(5):531-40.

6. Braun V, Clarke V. Using thematic analysis in psychology. Qual Res Psychol. 2006;3(2):77-101.

## A28 Pebbles, rocks, and boulders: The implementation of a school-based social engagement intervention for children with autism

### Jill Locke^1^, Courtney Benjamin Wolk^2^, Colleen Harker^3^, Anne Olsen^4^, Travis Shingledecker^2^, Frances Barg^2^, David Mandell^2^, Rinad S. Beidas^2^

#### ^1^Department of Speech and Hearing Sciences, University of Washington, Seattle, WA, 98195, USA; ^2^Department of Psychiatry, University of Pennsylvania, Philadelphia, PA, 19104, USA; ^3^Department of Psychology, University of Washington, Seattle, WA, 98195, USA; ^4^New York University School of Medicine, New York, NY, 10016, USA

##### **Correspondence:** Jill Locke (jjlocke@uw.edu) – Department of Speech and Hearing Sciences, University of Washington, Seattle, WA, 98195, USA

**Background**

Few evidence-based practices for children with autism have been successfully implemented and sustained in schools [1]. This study examined the perspectives of school personnel on implementing a social engagement intervention for elementary-aged children with autism.

**Materials and methods**

Semi-structured interviews were conducted with administrators (*N* = 15), teachers (*N* = 10), and other school personnel (*N* = 14) who participated in a randomized controlled trial of a school-based social engagement intervention for children with autism. Participants were asked about: 1) school factors that affect the general implementation of evidence-based practices; 2) their specific experiences implementing the social engagement intervention; and 3) barriers to and facilitators of implementing the social engagement intervention.

**Results**

Data were analyzed using a modified grounded theory approach. General (e.g., implementation process, leadership, support, staff) and intervention-specific (e.g., staff, barriers, facilitators) implementation themes were identified. Common intervention-specific barriers included limited recess time, resources, and autism-specific training. Common facilitators included support (e.g., provision of materials or space, extra time for recess), communication between staff members and administrators about the intervention (e.g., planning meetings), receiving positive feedback about the intervention from colleagues, and directly observing student progress.

**Conclusions**

These findings suggest that a variety of factors should be considered when implementing evidence-based practices in schools and that implementing social engagement interventions for children with autism may require additional specific support for implementation. With complex autism evidence-based practices, successful implementation may be related to the implementation process and supports at the school setting rather than the core components of the intervention.

**Reference**

1. Dingfelder HE, Mandell DS. Bridging the research-to-practice gap in autism intervention: An application of diffusion of innovation theory. J Autism Dev Disord. 2011;41:597- 609.

## A29 Problem Solving Teletherapy (PST.Net): A stakeholder analysis examining the feasibility and acceptability of teletherapy in community based aging services

### Marissa C. Hansen^1^, Maria P. Aranda^2^, Isabel Torres-Vigil^3^

#### ^1^School of Social Work, California State University, Long Beach, CA, 90802, USA; ^2^School of Social Work, University of Southern California, Los Angeles, CA, 90089, USA; ^3^Graduate College of Social Work**,** University of Houston, Houston, TX, 77004, USA

##### **Correspondence:** Marissa C. Hansen (marissa.hansen@csulb.edu) – School of Social Work, California State University, Long Beach, CA, 90802, USA

**Background**

Effective psychosocial depression treatments exist for older-adults, yet individual and organizational barriers impact use [1]. Teletherapy services are a cost-savings approach to ease access for older-adults with limited mobility [2,3]. In this study, factors framed by Diffusion of Innovation Theory [4,5] were examined to understand perceived feasibility and acceptability considerations by staff and clients in using Problem Solving Teletherapy (PST.net) in urban community-based aging services.

**Materials and methods**

We conducted semi-structured interviews and focus groups with a purposive sample of stakeholders from an older-adult social service agency that included management staff (*N* = 4), clinicians (*N* = 5), and older-adult clients (*N* = 14). Questions were asked around perceived viability and effectiveness of a PST.net approach to support client needs and interests, while maximizing clinician capacity to provide care.

**Results**

Using methods informed by grounded theory, themes emerged around norms and attitudes on comfort with technology that impacted openness to use by providers and clients, clinical considerations that optimized interactions and client outcomes, and organizational limitations around infrastructure to manage technology use in daily operations. Participants recommended an adapted version of PST.net that centered on using the technology to provide supportive counseling and case-management with a mix of in-person and teletherapy contact.

**Conclusions**

Findings present implications of teletherapy in providing services to homebound urban dwelling older-adults and increasing capacity of providers in managing ongoing client needs [2,3].

Though PST.net as a singular modality was not viable, an adaptive version did appear feasible while meeting the varying levels of readiness of use and current trends of teletherapy in community-based care.

**Acknowledgement**

Herzstein Foundation and Consortium on Aging, Houston Texas, Roybal Institute on Aging, University Southern California, School of Social Work

**References**

1. Chapman DP, Perry GS. Depression as a major component of public health for older adults. Prev Chronic Dis. 2008;5(1):A22.

2. Darkins A, Ryan, P, Kobb R, Foster L, Edmonson E, Wakefield B, Lancaster AE. Care coordination/home telehealth: The systematic implementation of health informatics, home telehealth, and disease management to support the care of veteran patients with chronic conditions. Telemed E Health. 2008;14(10):1118-1126.

3. Gellis, ZD, Kenaley B, McGinty J, Bardelli E, Davitt, J, Ten Have T. Outcomes of a telehealth intervention for homebound older adults with heart or chronic respiratory failure: A randomized controlled trial. Gerontologist. 2012;52(4):541-552.

4. Powell BJ, McMillen JC, Proctor EK, Carpenter CR, Griffey RT, Bunger AC, Glass JE, York JL. A compilation of strategies for implementing clinical innovations in health and mental health. Med Care Res Rev. 2012 Apr 1;69(2):123-57.

5. Rogers, E. M. A. prospective and retrospective look at the diffusion model. J Health Commun. 2004;9(S1):13-19.

## A30 A case of collaborative intervention design eventuating in behavior therapy sustainment and diffusion

### Bryan Hartzler (hartzb@uw.edu)

#### Alcohol & Drug Abuse Institute, University of Washington, Seattle, WA, 98105, USA

**Background**

Collaborative intervention design, a process that pools a therapy purveyor’s conceptual expertise and setting leaders’ contextual insights to tailor sustainable therapeutic programming, was applied to contingency management in a type III implementation-effectiveness hybrid trial at an opiate treatment setting. Prior reports [1,2] detail the collaborative intervention design process, and document successful staff training, intervention effectiveness, and leadership support for its sustainment. Current work summarizes post-trial reports of intervention sustainment efforts.

**Materials and methods**

To examine intervention sustainment efforts, a purposeful sampling approach targeted the two setting staff who served as local implementation leaders during the trial. The therapy purveyor contacted each via telephone biannually over a 24-month post-trial period, using open-ended probes to elicit information about intervention sustainment in the setting.

**Results**

Local implementation leaders outlined several encouraging developments. Collectively, their reports: 1) confirmed continuous intervention sustainment for 24 months, 2) attributed perpetual staff enthusiasm for the intervention to setting director involvement in its design, 3) revealed diffusion of the intervention to two affiliated opiate treatment settings amidst expansion of the parent organization, 4) noted creation of a dedicated position for multisite coordination of the intervention, and 5) indicated setting plans to apply collaborative intervention design in future development of additional contingency management programming.

**Conclusions**

This work expands on previously published accounts of trial success after collaborative design of a contingency management intervention at this opiate treatment setting. Given reports of continual sustainment and eventual diffusion, collaborative intervention design may merit application to other empirically supported behavior therapies and health settings.

**Acknowledgements**

This research was supported K23 DA025678.

**References**

1. Hartzler B, Jackson TR, Jones BE, Beadnell B, Calsyn DA. Disseminating contingency management: Impacts of staff training and implementation at an opiate treatment program. J Subst Abuse Treat*.* 2014;46:429-438.

2. Hartzler B. Building a bonfire that remains stoked: Sustainment of a contingency management intervention through collaborative design. Subst Abuse Treat Prev Policy. 2015;10:30.

## A31 Implementation of suicide risk prevention in an integrated delivery system: Mental health specialty services

### Bradley Steinfeld, Tory Gildred, Zandrea Harlin, Fredric Shephard

#### Group Health Cooperative, Seattle, WA, USA

##### **Correspondence:** Bradley Steinfeld (steinfeld.b@ghc.org) – Group Health Cooperative, Seattle, WA, USA

**Background**

Suicide is the major safety concern for patients who are seen in behavioral health specialty settings. The National Action Alliance for Suicide Prevention [1] has identified essential dimensions of suicide prevention: zero suicide culture, screening for suicide at every visit, structured suicide assessment for patients identified as at risk, and crisis response plan including lethal means removal. Yet, this has been found to not consistently occur in usual practice.

**Materials and methods**

Suicide risk assessment was identified as key strategic business initiative. A continuous improvement process engaged front line staff in designing a safe and efficient work flow. Tools were developed to evaluate implementation at clinic and provider level.

**Results**

Screening for suicide risk (Patient Health Questionnaire; PHQ-9) [2] increased from 15 % to 90 % of all adult outpatient mental health visits. For those patients identified as at risk for suicide, structured suicide risk assessment (i.e., Columbia Suicide Severity Scale) [3] increased from 20 % of visits to 90 % of visits.

**Conclusions**

Systematic use of screening and assessment tools was successful in increasing suicide risk assessment in mental health specialty clinics. Issues that emerged were that lethal means protocols were not consistently implemented, there was uncertainty regarding what impact process improvement had on actual suicide rate, and management of patients with chronic suicidality continued to be a challenge.

**References**

1. National Action Alliance for Suicide Prevention: Clinical Workforce Preparedness Task Force. Suicide prevention and the clinical workforce: Guidelines for training. Washington, D.C.: Author, 2014*.*

2. Kroenke K, Spitzer RL, Williams JB. The PHQ-9: Validity of a brief depression severity measure. J Gen Intern Med. 2001;16:606-613.

3. Posner K, Brown GK, Stanley B, Brent DA, Yershova KV, Oquendo MA, Currier GW, Melvin GA, Greenhill L, Shen S, Mann JJ. The Columbia–Suicide Severity Rating Scale: Initial validity and internal consistency findings from three multisite studies with adolescents and adults. Am J Psychiatry. 2011 Dec 1; 168(12):1266-1277.

## A32 Implementation team, checklist, evaluation, and feedback (ICED): A step-by-step approach to Dialectical Behavior Therapy program implementation

### Matthew S. Ditty^1^, Andrea Doyle^2^, John A. Bickel III^1^, Katharine Cristaudo^1^

#### ^1^The Ebright Collaborative, LLC, Wilmington, DE, 19802, USA; ^2^School of Social Policy and Practice, University of Pennsylvania, Philadelphia, PA, 19104, USA

##### **Correspondence:** Matthew S. Ditty (mditty@ebrightcollaborative.com) – The Ebright Collaborative, LLC, Wilmington, DE, 19802, USA

**Background**

Organizational support has been identified as a key facilitator of Dialectical Behavior Therapy (DBT) implementation [1,2]. DBT is a psychosocial treatment that effectively reduces symptoms of borderline personality disorder, suicidality, non-suicidal self-injury, and severe emotional and behavioral dyscontrol [3]. While organizational barriers and facilitators for DBT implementation have been identified, specific behavioral strategies remain unknown.

**Materials and methods**

This research was conducted in two phases. In Phase 1, a secondary thematic analysis of qualitative data from Ditty et al. [1] was conducted to locate behavioral strategies associated with known facilitators of DBT program implementation. In Phase 2, the strategies were refined and piloted in an iterative process of implementing a free standing DBT program, and case material was collected.

**Results**

Phase 1 results were organized as a step-by-step approach per the acronym ICED – implementation team, checklist, evaluation and feedback, and DBT skills. Phase 2 results illustrate examples of each step of ICED in action (e.g. behavioral strategies describing the formation of an actual implementation team were recorded, including emailing interested parties and meeting informally at a coffee shop).

**Conclusions**

ICED is a series of strategies identified by the present research and successfully utilized to implement an actual DBT program. Applied case material illustrates the steps in action, increasing the utility of ICED for those seeking to implement a DBT program. Future research is recommended for refining and testing ICED across organizations and settings.

**References**

1. Ditty MS, Landes SJ, Doyle A, Beidas RS. It takes a village: A mixed method analysis of inner setting variables and Dialectical Behavior Therapy implementation. Adm Policy Ment Hlth. 2015 Nov 1; 42(6):672-81.

2. Swales MA, Taylor B, Hibbs RA. Implementing dialectical behaviour therapy: Programme survival in routine healthcare settings. Journal of Mental Health. 2012 Dec 1; 21(6):548-55.

3. Lynch TR, Trost WT, Salsman N, Linehan MM. Dialectical behavior therapy for borderline personality disorder. Annu. Rev. Clin. Psychol. 2007 Apr 27; 3:181-205.

4. Posner K, Brown GK, Stanley B, Brent DA, Yershova KV, Oquendo MA, Currier GW, Melvin GA, Greenhill L, Shen S, Mann JJ. The Columbia–Suicide Severity Rating Scale: Initial validity and internal consistency findings from three multisite studies with adolescents and adults. Am J Psychiatry. 2011 Dec 1; 168(12):1266-1277.

## A33 The challenges in implementing multiple evidence-based practices in a community mental health setting

### Dan Fox, Sonia Combs

#### Lutheran Community Services Northwest, SeaTac, WA, 98188, USA

##### **Correspondence:** Dan Fox (dfox@lcsnw.org) – Lutheran Community Services Northwest, SeaTac, WA, 98188, USA

**Background**

Lutheran Community Services Northwest (LCSNW) is a community mental health agency that implements multiple evidence-based practices (EBPs) for children, families, and adults. EBPs include: components-based CBT, parent-child interaction therapy, and cognitive processing therapy. Given the numerous EBPs offered at LCSNW, unique challenges arise with respect to maintaining model fidelity, ensuring adequate training in each model, and weaving EBPs into agency culture. Moreover, clinicians struggle to creatively implement each model for a diverse client population.

**Materials and methods**

Approximately 26 masters-level clinicians and 30 interns at the agency were interviewed. Both positive and negative opinions were gathered in supervisory sessions.

**Results**

Overall, the LCSNW case study found high attention to self-care as a factor associated with addressing staff turnover. Given the multiplicity of EBPs, developing an integrated training system was proven highly beneficial at LCSNW, including: 1) EBP information during orientation, 2) ongoing in-house training, and 3) attending outside training in line with EBPs. Moreover, maintaining highly trained supervisory staff to continually educate the inevitable staff turnover was both critical and ultimately challenging. Finally, having routine discussions of how EBPs link to clinicians’ desire to help clients improve increases connection to models, producing positive impacts on fidelity.

**Conclusions**

LCSNW is an exemplary agency, highlighting the benefits observed from fostering an agency-wide culture supporting the use of EBPs and attending to clinician wellbeing. To mirror these benefits, LCSNW suggests creating buy-in at all levels, as well as weaving the EBP lexicon into all aspects of the agency, not simply those relevant to treatment.

## A34 Using electronic health record technology to promote and support evidence-based practice assessment and treatment intervention

### David H. Lischner (dlischner@valantmed.com)^1,2,3^

#### ^1^Valant Medical Solutions, Seattle, WA, 98121, USA; ^2^Evidence Treatment Centers of Seattle, Seattle, WA, 98101, USA; ^3^University of Washington, Seattle, WA, USA

##### **Correspondence:** David H. Lischner (dlischner@valantmed.com) – University of Washington, Seattle, WA, USA

**Background**

The first generation of electronic health record (EHR) software was built with static data models and pre-defined functions. Although designed for process automation, the technology made it difficult for EHRs to adjust when processes changed. Because of this, it was difficult for EHRs to incorporate advances in evidence-based practice (EBP) service delivery.

**Materials and methods**

Valant built an EHR platform on a dynamic data model. This data design enables the software to more easily adapt new treatment protocols and incorporate them into prompted workflows within the EHR. The platform technology supports ecosystems of content producers and consumers.

Content producers are clinical academic researchers developing new EBP’s. Content consumers are clinical organizations enabled by platform technology to adhere to best practice workflows to produce better patient outcomes.

**Results**

The EHR platform will become a distribution method for new academic findings. Advances in evidence-based interventions can now be incorporated into EHR workflows and more easily implemented at the service delivery level. The dynamic data model also makes it easier to aggregate and report on the data captured to enable continuous improvement of these workflows.

**Conclusions**

EHR platforms can support better clinical outcomes at scale by helping to disseminate EBP’s as continuously improving best practice workflows.

## A35 Are existing frameworks adequate for measuring implementation outcomes? Results from a new simulation methodology

### Richard A. Van Dorn, Stephen J. Tueller, Jesse M. Hinde, Georgia T. Karuntzos

#### RTI International, Durham, NC, 27709, USA

##### **Correspondence:** Stephen J. Tueller (stueller@rti.org) – RTI International, Durham, NC, 27709, USA

**Background**

Existing implementation frameworks guide the measurement of implementation outcomes. However, empirically validating implementation outcomes, for example those identified by Proctor and colleagues [1], is often challenged by limited data sources, a constrained item pool, and inadequate sample size. In order to establish the minimum requirements for sufficient power to detect Proctor and colleagues’ 2011 eight implementation outcomes going forward, we used an exploratory factor analysis simulation.

**Materials and methods**

We assumed a fixed population and sampled from an infinite pool of items to simulate realistic item selection processes, where data can be collected from only one sample and there is limited control in selecting the loadings, crossloadings, and error variances from the pool of potential items. Our simulation modeled sample size (200, 500, 1000), item pool size (24, 40, 80), item response distribution (normal, binary, Likert), and a range of (cross)loadings and error variances.

**Results**

Results show the adjusted Bayesian Information Criterion was the most accurate factor extraction criterion, and that item pool size and sample size had larger impacts on correctly detecting eight factors than ideal item characteristics (e.g., high loadings, low crossloadings, low error variance) across response distributions.

**Conclusions**

Implementation researchers undertaking instrument development should focus primarily on a large item pool size and secondarily on a large sample size as these will have the biggest impact on correct extraction of hypothetical factors. Combining extant measures without factor analyzing them in a common sample is inadequate since independent measure development does not provide evidence for factor uniqueness.

**Reference**

1. Proctor E, Silmere H, Raghavan R, Hovmand P, Aarons G, Bunger A, Griffey R, Hensley M. Outcomes for implementation research: Conceptual distinctions, measurement challenges, and research agenda. Admn Policy Ment Health. 2011 Mar 1; 38(2):65-76.

## A36 Taking global local: Evaluating training of Washington State clinicians in a modularized cognitive behavioral therapy approach designed for low-resource settings

### Maria Monroe-DeVita^1^, Roselyn Peterson^1^, Doyanne Darnell^2^, Lucy Berliner^3^, Shannon Dorsey^4^, Laura K. Murray^5^

#### ^1^Department of Psychiatry & Behavioral Sciences, University of Washington, Seattle, WA, 98102, USA; ^2^Department of Psychiatry & Behavioral Sciences, Harborview Medical Center, University of Washington, Seattle, WA, 98122, USA; ^3^Harborview Center for Sexual Assault and Traumatic Stress, University of Washington, Seattle, WA, 98122, USA; ^4^Department of Psychology, University of Washington, Seattle, WA, 98195, USA; ^5^Department of Mental Health, Johns Hopkins Bloomberg School of Public Health, Baltimore, MD, 21205, USA

##### **Correspondence:** Maria Monroe-DeVita (mmdv@uw.edu) – Department of Psychiatry & Behavioral Sciences, University of Washington, Seattle, WA, 98102, USA

**Background**

The Common Elements Treatment Approach (CETA) is a modularized cognitive behavioral treatment to address posttraumatic stress, anxiety, and depression among people in low- and middle-income countries [1]. CETA is efficient, low-cost, accessible, and utilized by clinicians from diverse backgrounds [2]. Implementation in United States community mental health (CMH) agencies seems prudent. The primary outcome of this evaluation is to understand the feasibility and benefits of training providers in CETA.

**Materials and methods**

In December 2014, 45 clinicians and 13 supervisors from nine CMH agencies in Washington State participated in CETA training. Providers evaluated themselves on 17 core CETA skills pre- and post-training, as well as six months post-training. Client cases were presented in bi-weekly consultation calls and consultants assessed case presentation quality.

**Results**

Self-perception of all skills improved after training and consultation. A repeated measures ANOVA showed a significant increase in provider (*N* = 44) self-report of skill from 57.2 to 65.3, (*p* = .013). Providers also answered open-ended implementation questions regarding delivery of CETA. Using specific CBT tools such as the cognitive triangle was endorsed by 31 % for facilitating easier delivery of CETA. In contrast, struggling with specific components (e.g., trauma exposure) was endorsed by 18 % of providers as a barrier to delivering CETA to their CMH clients.

**Conclusions**

CETA is a novel approach that offers many opportunities to greatly impact the way public health treats anxious, depressed, and trauma-exposed populations. By focusing on symptom reduction, CETA has been found to be successful when implemented in the public mental health context.

**References**

1. Murray L, Dorsey S, Haroz E, Lee C, Alsiary M, Haydary A, Weiss W, Bolton P. A common elements treatment approach for adult mental health problems in low- and middle-income countries. Cogn Behav Pract. 2014;21:111-123.

2. Bolton P, Lee C, Haroz E, Murray L, Dorsey S, Robinson C, Ugueto A, Bass J. A trans diagnostic community-based mental health treatment for comorbid disorders: Development and outcomes of a randomized controlled trial among Burmese refugees in Thailand. PLoS Med. 2014;11(11):1-16.

## A37 Attitudes toward evidence-based practices across therapeutic orientations

### Yevgeny Botanov^1,2^, Beverly Kikuta^1^, Tianying Chen^1^, Marivi Navarro-Haro^1^, Anthony DuBose^2^, Kathryn E. Korslund^1^, Marsha M. Linehan^1^

#### ^1^Behavioral Research & Therapy Clinics, Department of Psychology, University of Washington, Seattle, WA, 98195, USA; ^2^Linehan Institute/Behavioral Tech, LLC, Seattle, WA, 98195, USA

##### **Correspondence:** Yevgeny Botanov (ybotanov@uw.edu) – Linehan Institute/Behavioral Tech, LLC, Seattle, WA, 98195, USA

**Background**

Mental health providers’ negative attitudes toward evidence-based practice (EBP) may be influenced by therapeutic orientation and may impede implementation efforts. Thus, the present study examined changes in attitudes toward EBPs and the relationship between attitudes and therapeutic orientations in a sample of practitioners attending a training to implement an evidence-based intervention.

**Materials and methods**

Participants attended two five-day workshops (17 trainings, *N* = 449), separated by a six-month period, designed to implement dialectical behavior therapy (DBT). EBP attitudes were assessed via the Evidence-Based Practice Attitude Scale and participants endorsed the therapeutic approach they most commonly employ when treating individuals with borderline personality disorder. Participants were divided into a cognitive behavior therapy (CBT)/DBT (*N* = 345) or non-CBT/DBT (e.g., client centered, psychodynamic, interpersonal; *N* = 114) group.

**Results**

Participants ranged in degree type including four-year or less (7 %), masters (60 %), and PhD/PsyD/MD (30 %). As hypothesized, openness to adopting an empirical-derived practice was significantly lower in the non-CBT/DBT group. Repeated-measures analyses of variance revealed no significant group-by-time interactions. However, openness scores increased and negative views of EBPs decreased significantly for both groups over the six-month training period.

**Conclusions**

The present study finds that attitudes toward EBPs differ based on therapeutic orientations. However, a training designed to implement an EBP – in this case DBT – is associated with improvement in openness to EBPs and reduced negative feelings toward EBPs independent of preexisting therapeutic approach. Further examinations via controlled trials are warranted to generalize the relationships between therapeutic orientations, attitudes toward EBPs, and EBP implementation success.

## A38 Predicting the use of an evidence-based intervention for autism in birth-to-three programs

### Colleen M. Harker, Elizabeth A. Karp, Sarah R. Edmunds, Lisa V. Ibañez, Wendy L. Stone

#### Department of Psychology, University of Washington, Seattle, WA, 98195, USA

##### **Correspondence:** Colleen M. Harker (charker@uw.edu) – Department of Psychology, University of Washington, Seattle, WA, 98195, USA

**Background**

A research-to-practice gap exists in the use of evidence-based interventions (EBIs) for children with autism in community practice [1]. To increase the use of EBIs, we must understand the context in which providers use them [2, 3].

**Materials and methods**

This study examined factors associated with the use of an EBI for children with autism by community providers (*N* = 94) across Washington State. Providers attended one-day workshops on Reciprocal Imitation Training (RIT), an autism-specialized behavioral intervention, [4] and rated the acceptability and feasibility of RIT and the implementation climate of their workplace immediately post-training and at three- or six-month follow-up [5] and reported whether they used RIT at follow-up.

**Results**

Two-by-two repeated measures ANOVAs revealed main effects for time and RIT use, such that provider ratings of RIT’s acceptability, feasibility, and implementation climate declined between post training and follow-up (*p’*s < .01) and were lower for providers *not* using RIT at follow-up (*p’*s < .01). There were significant interactions between time and RIT use for acceptability and implementation climate, such that ratings declined over time only for providers *not* using RIT at follow-up (*p’*s < .01). Logistic regressions revealed that post-training ratings of acceptability (OR = 3.56, *p* = .01) and implementation climate (OR = 3.59, *p* = .03) predicted RIT use.

**Conclusions**

These results highlight the importance of understanding the environment in which an intervention is delivered. By identifying factors associated with intervention uptake, we can disseminate interventions that are effective and appropriate for use in community practice.

**References**

1. Lord C. Autism: From research to practice. Am Psychol. 2010 Nov; 65(8):815-826.

2. Dingfelder HE, Mandell DS. Bridging the research-to-practice gap in autism intervention: An application of diffusion of innovation theory. J Autism Dev Disord. 2011 May; 41:597-609.

3. Proctor E, Silmere H, Raghavan R, Hovmand P, Aarons G, Bunger A, Griffey R, Hensley M. Outcomes for implementation research: Conceptual distinctions, measurement challenges, and research agenda. Adm Policy Ment Health. 2011 Mar; 38(2):65-76.

4. Ingersoll B. The social role of imitation in autism: Implications for the treatment of imitation deficits. Infants Young Child. 2008 Apr; 21(2):107-19.

5. Chafouleas SM, Briesch AM, Riley-Tillman TC, McCoach DB. Moving beyond assessment of treatment acceptability: An examination of the factor structure of the Usage Rating Profile-Intervention (URP-I). Sch Psychol Q. 2009 Mar; 24(1):36.

## A39 Supervision practices and improved fidelity across evidence-based practices: A literature review

### Mimi Choy-Brown **(**mimi.choybrown@nyu.edu)

#### Silver School of Social Work, New York University, New York, NY, 10003, USA

**Background**

Behavioral health service settings urgently need onsite strategies to integrate evidence-based practices (EBPs). EBP implementation often relies on direct practice supervision. However, limited understanding remains of common supervisory behaviors necessary for fidelity. This study aimed to review literature of the relationship between supervision and fidelity across EBPs.

**Materials and methods**

This review involved a multi-stage process [1] using Substance Abuse and Mental Health Administration’s (SAMHSA) National Registry of Evidence-Based Programs and Practices (NREPP) to identify supervision practices associated with EBPs. Inclusion criteria included outpatient interventions for adolescents/adults that met SAMHSA quality assessment rating of 3+ for the supporting evidence of effectiveness and implementation. EBP research articles and supervision manuals were reviewed.

**Results**

From the 140 EBPs identified, 12 met inclusion criteria and had published empirical findings of the relationship between supervision and fidelity. These 12 EBPs were rooted in three EBP supervision models including Motivational Interviewing (MI), Cognitive-Behavioral Therapy (CBT), and MultiSystemic Therapy (MST). Review of supervision manuals revealed similar requirements for structure (more than 60 minutes per week) data (feedback from taped sessions), content (rehearsal, feedback, and developmental planning), and interpersonal experience (positive, collaborative). Differences identified were specific to EBP requirements (e.g., mirroring MI principles). Limited direction provided for adaptation to contextual factors (e.g. climate, provider expertise).

**Conclusions**

Findings suggest common supervisory behaviors improve fidelity. However, real world service contexts may not consistently provide the structure, data or content identified in trials and manuals [2]. More knowledge of efficient and effective supervision models adaptive to contextual constraints is needed to improve delivery of EBP.

**References**

1. Whittemore R, Knaft K. The integrative review: Updated methodology. J Adv Nurs. 2005;52(5):546-553.

2. Dorsey S, Pullmann MD, Deblinger E, Berliner L, Kerns SE, Thompson K, Unützer J, Weisz JR, Garland AF. Improving practice in community-based settings: A randomized trial of supervision—study protocol. Implement Sci. 2013 Aug; 10;8(89):1-2.

## A40 Beyond symptom tracking: clinician perceptions of a hybrid measurement feedback system for monitoring treatment fidelity and client progress

### Jack H. Andrews, Benjamin D. Johnides, Estee M. Hausman, Kristin M. Hawley

#### Department of Psychological Sciences, University of Missouri, Columbia, Missouri, 65211, USA

##### **Correspondence:** Jack H. Andrews (andrewsjh@missouri.edu) – Department of Psychological Sciences, University of Missouri, Columbia, Missouri, 65211, USA

**Background**

A growing body of research suggests that measurement feedback systems (MFSs) have the potential to produce widespread improvements in mental healthcare quality [1]. Previous studies have focused on MFSs that assess client factors such as symptoms, functioning, and therapeutic alliance, but expanding the scope of MFSs to also target clinicians’ fidelity to specific evidence-based practices (EBPs) may offer additional utility for enhancing EBP implementation efforts and client outcomes. The current study presents preliminary findings from a community- based pilot test of a MFS prototype that assesses clinician fidelity to evidence-based cognitive behavioral therapy (CBT) for youth anxiety, depression, trauma, and/or disruptive behaviors,

in addition to client symptoms and therapeutic alliance.

**Materials and methods**

Therapists (*N* = 33) completed a qualitative interview about their perceptions of the MFS’s potential for adoption and use in routine practice. Twenty-one interviews were transcribed, and conventional qualitative content analysis was employed to identify salient themes and develop an initial coding frame. The proportion of missing data from clinicians and clients was also examined as an indicator of implementation feasibility.

**Results**

The initial coding frame consisted of four main categories (Utility, Limitations, Potential Improvements, and Potential Barriers), each having five subcategories (Questionnaire and/or Process, CBT Feedback and Suggestions, Client Perspectives, Symptom Tracking, and Progress Note). Thirty-two therapists used the MFS at least once; however, only 16 (50 %) therapists and seven (22 %) clients used it for the full requested duration of participation.

**Conclusions**

The MFS prototype is largely acceptable to community therapists, but potential for adoption is limited by concerns with appropriateness, feasibility, and sustainability.

**Acknowledgements**

Supported by NIMH Grant R21 MH090460 (PI: Kristin M. Hawley, Ph.D.)

**Reference**

1. Landes SJ, Carlson EB, Ruzek JI, Wang D, Hugo E, DeGaetano N, Chambers JG, Lindley SE. Provider-driven development of a measurement feedback system to enhance measurement-based care in VA mental health. Cogn Behav Pract. 2015 Feb 28; 22(1):87-100.

## A41 A guideline decision support tool: From creation to implementation

### Beth Prusaczyk^1^, Alex Ramsey^2^, Ana Baumann^1,3^, Graham Colditz^2,3^, Enola K. Proctor^1,3^

#### ^1^George Warren Brown School of Social Work, Washington University in St. Louis, St. Louis, MO, 63130, USA; ^2^School of Medicine, Washington University in St. Louis, MO, 63130, USA; ^3^Institute for Public Health, Washington University in St. Louis, St. Louis, MO, 63130, USA

##### **Correspondence:** Beth Prusaczyk (beth.prusaczyk@wustl.edu) – ^1^George Warren Brown School of Social Work, Washington University in St. Louis, St. Louis, MO, 63130, USA

**Background**

While clinical practice guidelines are important aides to delivery of evidence-based practices, their implementation is problematic. Investigators often conduct clinical practice guideline (CPGs) research in substantive-area silos, unaware of the potentially helpful perspectives of implementation research. The Dissemination and Implementation Research Core (DIRC) of the Institute of Clinical and Translational Sciences (ICTS) at Washington University in St. Louis developed a decision support tool to support CPG research and to foster collaboration on cross- cutting CPG implementation issues.

**Materials and methods**

DIRC leadership facilitated a meeting of implementation researchers and investigators interested in CPG research. The meeting revealed confusion about different research purposes, including CPG creation, effectiveness testing, modification, and implementation.

**Results**

We developed a flowchart distinguishing between different CPG-related research aims. The tool helps investigators clarify whether they wish to create new CPGs, study CPG effectiveness, modify CPGs, or implement CPGs. Those studying guideline implementation are directed to resources, including exemplar reports of CPG implementation and conceptual frameworks and methods for CPG research. The decision support tool has been refined through user feedback.

**Conclusions**

The CPG decision support tool is periodically updated by DIRC staff and is a helpful resource for the ICTS and DIRC. Fostering collaboration and providing tools to investigators is important in advancing and enhancing efficiency of implementation research.

**Acknowledgments**

This research was supported by NIMH Grant T32 MH019960 and NIH CTSA Grant UL1 TR000448.

## A42 Dabblers, bedazzlers, or total makeovers: Clinician modification of a common elements cognitive behavioral therapy approach

### Rosemary D. Meza^1^, Shannon Dorsey^1^, Shannon Wiltsey Stirman^2^, Georganna Sedlar^3^, Leah Lucid^1^

#### ^1^Department of Psychology, University of Washington, Seattle, WA, 98195, USA; ^2^National Center for PTSD, VA Palo Alto Health Care System and Stanford University, Menlo Park, CA, 94024, USA; ^3^Department of Psychiatry and Behavioral Sciences, University of Washington, Seattle, WA, 98102, USA

##### **Correspondence:** Rosemary D. Meza (rdmeza@uw.edu) – Department of Psychology, University of Washington, Seattle, WA, 98195, USA

**Background**

Clinician modification to evidence-based practices (EBP) has largely been discouraged; however, emerging views highlight the possibility for modification to improve EBP fit and sustainability [1, 2]. Common-elements approaches that specifically include flexibility may offer a solution to the fidelity-modification debate. However, few studies have examined modification to a common-element approach [3, 4] and, to our knowledge, none have examined predictors of modification to these approaches.

**Materials and methods**

This study examined the prevalence of clinician modification to a common-elements cognitive- behavioral therapy approach [5] and factors that predict clinician modification following a three- day intensive training. Clinicians (*N* = 99) reported on their intent to modify, intervention fit, EBP implementation climate, and confidence in delivering the intervention immediately post-training. At six months post-training, clinicians reported on the type of modifications performed and reasons for modifying.

**Results**

Ninety-three percent of clinicians reported at least one modification. Clinicians primarily modified with fewer than half of their cases and most frequently made more fidelity-consistent modifications (i.e., tailoring and tweaking, 62 %) as compared to fidelity-inconsistent modifications (i.e., removing core treatment elements, 34 %). The primary reasons for modifying were client-level needs (40.4 %) and clinician style or preference (38.8 %). Clinician intent to modify (B = .57, *p* < .01) and confidence in delivering the intervention (B = -.96, *p* < .01) predicted the number of clinician modifications.

**Conclusions**

The results suggest that intent to modify and confidence are important in explaining clinician modification to a common-elements approach and have implications for training and supervision efforts to maintain quality delivery of EBP.

**References**

1. Aarons GA, Green AE, Palinkas LA, Self-Brown S, Whitaker DJ, Lutzker JR, Silovsky JF, Hecht DB, Chaffin MJ. Dynamic adaptation process to implement an evidence-based child maltreatment intervention. Implement Sci. 2012 Apr 18; 7(32):1–9.

2. Chambers DA, Glasgow RE, Stange KE. The dynamic sustainability framework: addressing the paradox of sustainment amid ongoing change. Implement Sci. 2013;8:117.

3. Park AL, Chorpita BF, Regan J, Weisz JR, Research Network on Youth Mental Health. Integrity of evidence-based practice: are providers modifying practice content or practice sequencing. Admn Policy Ment Health. 2015 Mar 1;42(2):186–96.

4. Palinkas LA, Weisz JR, Chorpita BF, Levine B, Garland AF, Hoagwood KE, Landsverk J. Continued use of evidence-based treatments after a randomized controlled effectiveness trial: a qualitative study. Psychiatr Serv. 2013 Nov 1.

5. Dorsey S, Berliner L, Lyon AR, Pullmann MD, Murray, LK. A statewide common elements initiative for children’s mental health. J Behav Health Serv Res. 2014;1.

## A43 Characterization of context and its role in implementation: The impact of structure, infrastructure, and metastructure

### Caitlin N. Dorsey^1^, Brigid R. Marriott^2^, Nelson Zounlome^3^, Cara Lewis^1^

#### ^1^Department of Psychological and Brain Sciences, Indiana University, Bloomington, IN, 47405, USA; ^2^Department of Psychological Sciences, University of Missouri, Columbia, MO, 65211, USA; ^3^Department of Counseling and Educational Psychology, Indiana University, Bloomington, IN, 47405, USA

##### **Correspondence:** Caitlin N. Dorsey (cadorsey@indiana.edu) – Department of Psychological and Brain Sciences, Indiana University, Bloomington, IN, 47405, USA

**Background**

The role of the organization’s context has received limited attention, despite its likely critical influence on implementation success. The organization’s context consists of three components: structure (i.e., physical parts with direct system influence), infrastructure (i.e., indirect influence of supportive structural factors), and metastructure (i.e., organizational/individual cognitive-rule base), or SIM [1]. To characterize these under-studied components, this study explored 1) expression of SIM within an organization undergoing an implementation effort and 2) relation between SIM components and previously identified critical determinants to implementation.

**Materials and methods**

Data came from a mixed-methods needs assessment of a Cognitive Behavioral Therapy implementation project in a youth residential setting. For Aim 1, focus group transcripts (*N* = 7) of staff members (*N =* 53; e.g., therapist, managers) were qualitatively analyzed for frequency of SIM components. For Aim 2, staff (*N* = 99) completed the Evidence-Based Practice Attitude Scale (EBPAS) [2], the Impact of Infrastructure scale (IOI) [3] and self-report questionnaires assessing burnout, job satisfaction, stress. Correlations between determinants and IOI subscales were conducted.

**Results**

Transcript analysis revealed SIM components were emphasized by approximately half of the focus groups (44.95-67.97 %), with metastructure (23.84-39.76 %) the most frequently discussed component. Correlational analyses revealed significant relations indicating that the need for infrastructure to be flexible and adapted to support an implementation was positively associated with the EBPAS [2] (.45, *p* < .01), burnout (.26, *p* < .05), and stress (.41, *p* < .01).

**Conclusions**

SIM components dominated the needs assessment and infrastructure was significantly related to previously identified determinants. Longitudinal studies are needed to confirm the impact of SIM on implementation.

**References**

1. Stelk W, Slaton E. The role of infrastructure in the transformation of child–adolescent mental health systems. Admn Policy Ment Hlth. 2010 Mar 1; 37(1-2):100–10.

2. Aarons GA. Mental health provider attitudes toward adoption of evidence-based practice: The Evidence-Based Practice Attitude Scale (EBPAS). Ment Health Serv Res. 2004 Jun 1; 6(2):61–74.

3. Comtois K, Keough M, Lewis CC, Landes S: The Impact of Infrastructure Survey. 2012.

## A44 Effects of consultation method on implementation of cognitive processing therapy for post-traumatic stress disorder

### Cassidy A. Gutner^1,2^, Candice M. Monson^3^, Norman Shields^4^, Marta Mastlej^3^, Meredith SH Landy^3^, Jeanine Lane^3^, Shannon Wiltsey Stirman^5,6^

#### ^1^Department of Psychiatry, Boston University School of Medicine, Boston, MA, USA; ^2^National Center for PTSD at VA Boston Healthcare System, Boston, MA, USA; ^3^Ryerson University, Department of Psychology, Toronto, Canada; ^4^Veterans Affairs Canada, Montreal, Canada; ^5^National Center for Posttraumatic Stress Disorder (PTSD), Veterans Affairs (VA) Palo Alto Healthcare System, Palo Alto, CA, USA; ^6^Department of Psychiatry, Stanford University, Menlo Park, CA, 94304, USA

##### **Correspondence:** Cassidy A. Gutner (cgutner@bu.edu) – National Center for PTSD at VA Boston Healthcare System, Boston, MA, USA

**Background**

Limited evidence exists on effective implementation strategies. Quantitative data demonstrated greater symptom change over time with standard consultation versus no consultation or technology-enhanced consultation. This study examines potential reasons for differential outcomes and identifies system-, site- and provider-level barriers and facilitators to implementation through qualitative data analysis of the same dataset.

**Materials and methods**

Data from a recently completed study on post-workshop follow-up strategies on Cognitive Processing Therapy (CPT) clinician attitudes, and clinical outcomes in an effort to implement CPT in Veterans Affairs (VA) Canada’s Operational Stress Injury National Network. Two consultation strategies (standard and technology-enhanced with work sample review) were compared to no consultation. This study focuses on qualitative data from a subset of participants (*N* = 12) who were interviewed about CPT, training experience, and contextual factors that influence key implementation outcomes, with the Consolidated Framework for Implementation Research informing the interview guide [CFIR; 1]

**Results**

A directed content analysis, using a-priori codes based on CFIR constructs, suggested multilevel influences on implementation. Clinicians discussed the importance of consultation and identified challenges and relativeadvantages of each condition. Influential characteristics of individuals included consultant style, clinician style, and patient willingness to engage in a protocol treatment. The technology-enhanced group found technology to be both a help and a hinderance, and the no consultation group emphasized the importance of consultation for implementation of CPT.

**Conclusions**

Understanding multilevel factors that impact implementation and sustainability, including clinician views of the consultation strategy, are important for successful implementation and dissemination effort.

**Reference**

1. Damschroder LJ, Aron DC, Keith RE, Kirsh SR, Alexander JA, Lowery JC. Fostering implementation of health services research findings into practice: a consolidated framework for advancing implementation science. Implement Sci. 2009 Oct; 4(1):50.

## A45 Cross-validation of the Implementation Leadership Scale factor structure in child welfare service organizations

### Natalie K. Finn^1,2^, Elisa M. Torres^1,2^, Mark. G. Ehrhart^3^, Gregory A. Aarons^1,2^

#### ^1^Department of Psychiatry, University of California, San Diego, La Jolla, CA, 92093, USA; ^2^Child and Adolescent Services Research Center (CASRC), San Diego, CA, 92123, USA; ^3^Department of Psychology, San Diego State University, San Diego, CA, 92182, USA

##### **Correspondence:** Natalie K. Finn (nfinn@ucsd.edu) – Child and Adolescent Services Research Center (CASRC), San Diego, CA, 92123, USA

**Background**

The Implementation Leadership Scale [ILS; 1] is a brief and efficient measure to assess leader behaviors and actions that actively support effective implementation of evidence-based practices (EBPs). The ILS was originally validated with mental health clinicians. This study examines the ILS factor structure with child welfare service providers [2].

### ᅟ

**Methods** Participants were 214 service providers working in 12 child welfare organizations in California, Illinois, Washington, and Oklahoma. All participants completed the ILS, reporting on their immediate supervisor. Multilevel confirmatory factor analyses were conducted to examine the factor structure of the ILS, accounting for the nested data structure (i.e., service providers nested within 43 teams), and indicating a hypothesized second order factor structure.

**Results**

Multilevel confirmatory factor analyses showed good fit [3] to the hypothesized first (χ2(50) = 115.02, p < 0.001; CFI = 0.967, TLI = 0.956; RMSEA = 0.078; SRMR = 0.047) and second order factor structure (χ2(50) = 115.18, p < 0.001; CFI = 0.967, TLI = 0.956; RMSEA = 0.078; SRMR = 0.047). First order factor loadings ranged from 0.85-0.95 for Proactive Leadership, from 0.94-0.99 for Knowledgeable Leadership, 0.86-0.95 for Supportive Leadership, and 0.85-0.96 for Perseverant Leadership, and second order factor loadings ranged from 0.83-0.90.

**Conclusions**

The higher order factor structure of the ILS is robust indicating its utility in assessing leadership for implementation of EBPs in mental health and child welfare organizations.

**References**

1. Aarons GA, Ehrhart MG, Farahnak LR. The Implementation Leadership Scale (ILS): Development of a brief measure of unit level implementation leadership. Implement Sci. 2014;9(1):45.

2. Finn NK, Torres EM, Ehrhart MG, Roesch SC, Aarons GA. Cross-validation of the Implementation Leadership Scale (ILS) in child welfare service organizations. Child Maltreat. Forthcoming.

3. Hu L-T, Bentler PM. Cutoff criteria for fit indexes in covariance structure analysis: Conventional criteria versus new alternatives. Struct Equ Modeling. 1999; 6(1):1-55.

## A46 Sustainability of integrated smoking cessation care in Veterans Affairs posttraumatic stress disorder clinics: A qualitative analysis of focus group data from learning collaborative participants

### Carol A. Malte^1^, Aline Lott^1^, Andrew J. Saxon^1,2^

#### ^1^Center of Excellence in Substance Abuse Treatment and Education, Veterans Affairs Puget Sound Health Care System, Seattle, WA, 98108, USA; ^2^Department of Psychiatry and Behavioral Sciences, University of Washington, Seattle, WA, USA

##### **Correspondence:** Carol A. Malte (carol.malte@va.gov) – Center of Excellence in Substance Abuse Treatment and Education, Veterans Affairs Puget Sound Health Care System, Seattle, WA, 98108, USA

**Background**

To address high smoking rates among individuals with mental illness [1], clinical guidelines strongly recommend delivery of cessation treatment in mental health settings [2]. Studies indicate incorporating integrated care (IC) for smoking cessation into routine posttraumatic stress disorder (PTSD) treatment significantly increases long-term quit rates relative to standard care in Department of Veterans Affairs (VA) settings [3]. To facilitate implementation of IC, we conducted a learning collaborative involving multidisciplinary teams from six VA PTSD clinics.

**Materials and methods**

This evaluation consisted of four focus groups (clinicians, clinical champions, clinic directors and prescribers, *N* = 28) to assess how IC fits with clinic structure, necessary adaptions, and sustainability issues. We analyzed qualitative data for key themes using the PARiHS (Promoting Action on Research Implementation in Health Services) framework [4].

**Results**

Although participants were generally enthusiastic about IC, they experienced varying degrees of team and clinic consensus regarding treatment implementation. Emergent themes reflected shifting clinical environments (e.g. changing treatment modalities, transitioning from open-ended to time-limited clinics, and fluctuating staffing) that impacted treatment compatibility, team consensus and available resources. Participants emphasized the importance of adapting the treatment and treatment delivery to address such challenges; sharing across teams fostered adaptations. While teams had active clinic-level leadership support, higher-level support often was passive, which participants viewed as a potential barrier to sustainability and spread.

**Conclusions**

In changing clinical environments, challenges related to fit between treatments and clinic structure must be addressed to improve treatment compatibility and build team consensus. Cross- team sharing may promote treatment adaptations that help to overcome common implementation barriers.

**Acknowledgements**

Tobacco and Health: Policy and Programs, Clinical Public Health; VISN 20 Northwest Mental Health Research and Clinical Education Centers (MIRECC); VISN 6 Mid-Atlantic MIRECC; and the National Center for PTSD Dissemination and Training Division.

**References**

1. Lasser K, Boyd JW, Woolhandler S, Himmelstein DU, McCormick D, Bor DH. Smoking and mental illness: A population based prevalence study. JAMA. 2000;284:2606–2610.

2. Fiore MC, Jaén CR, Baker TB, Bailey WC, Benowitz NL, Curry SJ, Dorfman SF, Froelicher ES, Goldstein MG, Healton CG, Henderson PN, Heyman RB, Koh HK, Kottke TE, Lando HA, Mecklenburg RE, Mermelstein RJ, Mullen PD, Orleans CT, Robinson L, Stitzer ML, Tommasello AC, Villejo L, Wewers ME. Treating tobacco use and dependence. Clinical practice guideline. Rockville, MD: U.S. Department of Health and Human Services. Public Health Service. 2008.

3. McFall M, Saxon AJ, Malte CA, Chow B, Bailey S, Baker DG, Beckham JC, Boardman KD, Carmody TP, Joseph AM, Smith MW, Shih MC, Lu Y, Holodniy M, Lavori PW. Integrating tobacco cessation into mental health care for posttraumatic stress disorder: A randomized controlled trial. JAMA. 2010; 304:2485–2493.

4. Stetler CB, Damschroder LJ, Helfrich CD, Hagedorn HJ. A guide for applying a revised version of the PARIHS framework for implementation. Implement Sci. 2011;6:99.

## A47 Key characteristics of effective mental health trainers: The creation of the Measure of Effective Attributes of Trainers (MEAT)

### Meredith R. Boyd, Kelli Scott, Cara C. Lewis

#### Department of Psychological and Brain Sciences, Indiana University, Bloomington, IN, 47405, USA

##### **Correspondence:** Meredith Boyd (mereboyd@indiana.edu) – Department of Psychological and Brain Sciences, Indiana University, Bloomington, IN, 47405, USA

**Background**

Though a widely used training approach, single exposure didactic training in empirically supported treatments for mental health problems is largely ineffective in producing behavioral changes in providers [1, 2]. To our knowledge, research has yet to explore personal characteristics of trainers that could contribute to effective training. The current study aimed to create a valid and reliable measure of trainer characteristics.

**Materials and methods**

A pool of 58 positive and negative characteristics (i.e. enthusiastic, boring) was collected from relevant literature and from expert mental health trainers and graduate students in structured interviews to establish content validity. The preliminary measure was piloted with graduate students and revised accordingly, followed by expert measure developer review and revision to ensure face validity. Undergraduate participants completed the revised measure using a five- point Likert scale to evaluate trainers in four training videos. Four exploratory factor analyses (EFAs) were performed to delineate measure subscales and assess structural validity of the measure.

**Results**

A two-factor solution was revealed across EFAs. The first factor, “Charisma,” contained items (*N* = 14) that could facilitate positive relationships with the trainee. The second factor, “Credibility,” contained items (*N* = 7) emphasizing qualification of trainers. Across four EFAs, “Charisma” accounted for 42.0-54.7 % of item variance and had excellent internal consistency (*α* = 0.95-0.97); “Credibility” accounted for 13.1 %-24.1 % of variance and had good to excellent internal consistency (*α* = 0.86-0.94).

**Conclusions**

Results suggest the measure displays content, structural and face validity and is reliable. Future research should confirm the measure’s reliability, validity, and factor structure with representative samples of mental health trainees.

**References**

1. Sholomskas DE, Syracuse-Siewert G, Rounsaville BJ, Ball SA, Nuro KF, Carroll KM. We don’t train in vain: A dissemination trial of three strategies of training clinicians in cognitive-behavioral therapy. J Consult Clin Psychol 2005;73:106.

2. Jensen-Doss A, Cusack KJ, de Arellano MA. Workshop-based training in trauma-focused CBT: An in-depth analysis of impact on provider practices. Community Ment Health J. 2008; 44:227–244.

## A48 Coaching to improve teacher implementation of evidence-based practices (EBPs)

### Jennifer D. Pierce **(**jpierce@air.org)

#### Special Education, University of Washington, Seattle, Washington, 98101, USA

**Background**

Coaching consisting of cycles of observation, modeling, and feedback is an effective mechanism for increasing teachers’ implementation of EBPs, thereby leading to improved student outcomes [1]. Although positive alliance (i.e., teacher-coach relationship), is associated with increased fidelity of teacher practice [2] it is unknown if coaches’ use of alliance strategies leads to improved teacher practice. The purpose of this study was to test the effects of an intervention, the Teacher-Coach Support System (TCSS). Under the system, coaches planned to increase their use of alliance strategies. We hypothesized that coaches’ increased use of these strategies would lead to teachers’ improved implementation of EBPs.

**Materials and methods**

This study used a multiple baseline design and interviews to analyze the TCSS’ effects on coaches’ use of alliance strategies and teachers’ use of EBPs.

**Results**

Experimental results showed the TCSS led to increased use of alliance strategies. A treatment effect was found in all teachers’ praise, with means increasing between three to 10 times. Two teachers showed a treatment effect for the use of behavioral interventions. Qualitative data showed participants valued the TCCS as a tool for improving coaching and teaching.

**Conclusions**

Coaching is included in several implementation frameworks to support sustained uptake of EBPs [3,4,5], yet coaches are rarely taught how to improve their practice. Findings suggest the TCSS helped coaches identify specific strategies to use with teachers. Use of these strategies led to improved implementation of EBPs by teachers, indicating that alliance strategies play a powerful role in effective coaching.

**References**

1. Kretlow AG, Bartholomew CC. Using coaching to improve the fidelity of evidence-based practices: A review of studies. Teach Educ Spec Edu. 2010;1:278–99.

2. Wehby JH, Maggin DM, Partin TC, Robertson R. The impact of working alliance, social validity, and teacher burnout on implementation fidelity of the good behavior game. School Ment Health. 2012;4:22-33.

3. Fixsen DL, Naoom SF, Blase KA, Friedman RM. Implementation research: A synthesis of the literature. 2005.

4. Damschroder LJ, Aron DC, Keith RE, Kirsh SR, Alexander JA, Lowery JC. Fostering implementation of health services research findings into practice: a consolidated framework for advancing implementation science. Implement Sci. 2009;4:50.

5. Kitson A, Harvey G, McCormack B. Enabling the implementation of evidence based practice: A conceptual framework. Quality in Health Care. 1998;7:149–58.

## A49 Factors influencing the implementation of peer-led health promotion programs targeting seniors: A literature review

### Agathe Lorthios-Guilledroit^1,2^, Lucie Richard^1,3^, Johanne Filiatrault^2,4^

#### ^1^Institut de recherche en santé publique de l’Université de Montréal (IRSPUM), Montréal, Québec H3C 3 J7, Canada; ^2^Research Centre, Institut universitaire de gériatrie de Montréal, Montréal, Québec H3W 1 W5, Canada; ^3^Faculty of Nursing, Université de Montréal, Montréal, Québec H3C 3 J7, Canada; ^4^School of Rehabilitation, Faculty of Medicine, Université de Montréal, Montréal, Québec H3C 3 J7, Canada

##### **Correspondence:** Agathe Lorthios-Guilledroit (agathe.lorthios-guilledroit@umontreal.ca) – Research Centre, Institut universitaire de gériatrie de Montréal, Montréal, Québec H3W 1 W5, Canada

**Background**

Peer-led health promotion programs (PLHPP) targeting seniors are increasing in popularity. Indeed, several seniors are interested in volunteering activities including a health education component. Although there is growing evidence supporting the benefits of such programs, few efforts have been devoted to the study of factors influencing their implementation. Recent conceptual frameworks suggest that health promotion program implementation is influenced by factors including participants and providers’ characteristics, environmental context, as well as programs’ characteristics. This study reported findings from a literature review on factors influencing the implementation of PLHPP targeting seniors.

**Materials and methods**

MEDLINE, Embase, PsycINFO, CINAHL and ERIC databases were searched with keywords related to implementation, peers, health promotion programs, and seniors.

**Results**

Among the articles identified with our search strategy, 36 concerned the implementation of PLHPP targeting seniors. Participation rate was the most commonly used key indicator of successful implementation. Influencing factors identified in this review were in line with general conceptual frameworks on program implementation. However, specific factors related to peers (selection, training, etc.) were found to be particularly important in this review. Furthermore, influencing factors were often inferred from authors’ opinions rather than empirical data.

**Conclusions**

Findings from this literature review revealed a need for theoretical and empirical developments about factors influencing implementation of PLHPP targeting seniors. Addressing these gaps will be useful to advance research and practice.

**Acknowledgements**

ALG received scholarships from the Fonds de la recherche du Québec–Santé and the Université de Montréal. The Université de Montréal Public Health Research Institute supported this presentation.

## A50 Developing treatment fidelity rating systems for psychotherapy research: Recommendations and lessons learned

### Kevin Hallgren^1^, Shirley Crotwell^2^, Rosa Muñoz^3^, Becky Gius^3^, Benjamin Ladd^4^, Barbara McCrady^3^, Elizabeth Epstein^5^

#### ^1^Department of Psychiatry and Behavioral Sciences, University of Washington, Seattle, WA, 98195, USA; ^2^Boston Veteran Affairs Health Care System, Boston, MA, 02130, USA; ^3^Center on Alcoholism, Substance Abuse, and Addictions, University of New Mexico, Albuquerque, NM, 87131, USA; ^4^Department of Psychology, Washington State University Vancouver, Vancouver, WA, 98686, USA; ^5^Department of Psychiatry, University of Massachusetts Medical School, Worcester, MA, 01655, USA

##### **Correspondence:** Kevin Hallgren (khallgre@uw.edu) – Department of Psychiatry and Behavioral Sciences, University of Washington, Seattle, WA, 98195, USA

**Background**

Measuring fidelity to evidence-based treatments is a key component of dissemination and implementation research. However, developing reliable, valid, and clinically-relevant treatment fidelity measures remains a challenge. Although much of the literature has focused on theoretical and psychometric aspects of measure development, the literature often omits practical considerations for developing and using fidelity measures.

**Materials and methods**

The present study describes the development and testing of a treatment fidelity rating system used in couple-based alcoholism treatment. Over a three-year period, seven coders received extensive training and rated 74 components of treatment fidelity across 284 psychotherapy sessions from four clinical trials [1-4]. A theoretical model underlying the instrument was developed and its psychometric properties were tested.

**Results**

Inter-rater reliability indices for treatment integrity scales indicated variable agreement between coders. Many scales had poor or fair reliability. Nonetheless, several themes emerged based on coders’ and investigators’ impressions of their experiences developing, refining, using, and interpreting this coding system. Major challenges were identified in relation to (1) measure development (e.g., adapting existing fidelity measures for new treatments), (2) defining “treatment integrity” (e.g., conceptual and practical difficulties in rating various therapist behaviors), (3) process improvement (e.g., procedures for improving quality and efficiency of coder training and ongoing monitoring), and (4) inferring information from the ratings (e.g., improving clinical relevance and internal/external validity).

**Conclusions**

Behavioral coding is a challenging but important component of implementation research. Researchers conducting behavioral coding research should attend to the challenges identified here before and during behavioral coding research.

**References**

1. McCrady BS, Epstein EE, Cook S, Jensen N, Hildebrandt T. A randomized trial of individual and couple behavioral alcohol treatment for women. J Consult Clin Psychol. 2009;77(2):243–256.

2. McCrady BS, Epstein EE, Hallgren KA, Cook S, Jensen NK. Women with alcohol dependence: A randomized trial of couple versus individual plus couple therapy. Psychol Addict Behav. Forthcoming.

3. McCrady BS, Epstein EE, Hirsch LS. Maintaining change after conjoint behavioral alcohol treatment for men: Outcomes at six months. Addiction. 1999; 94:1381–1396.

4. McCrady BS, Noel NE, Stout RL, et al. Comparative effectiveness of three types of spouse involvement in outpatient behavioral alcoholism treatment. J Stud Alcohol. 1986;47:459–467.

## A51 Rapid translation of alcohol prevention science

### John D. Clapp, Danielle E. Ruderman

#### College of Social Work, The Ohio State University, Columbus, OH, 43210, USA

##### **Correspondence:** Danielle E. Ruderman (ruderman.5@osu.edu) – College of Social Work, The Ohio State University, Columbus, OH, 43210, USA

**Background**

Alcohol-related problems among college students are a serious public health issue. Approximately 1,800 students die each year due to alcohol-related unintentional injury [1]. The National Institute of Health has invested over 20 million in the past 20 years to address this issue. Nonetheless, very few institutions have implemented any evidence-based prevention measures [2]. This case study documents an attempt to develop adaptation/implementation checklists of an evidence-based college alcohol intervention.

**Materials and methods**

In 2013, a group of five senior College Alcohol and Other Drug (AOD) administrators and five alcohol prevention researchers met. The Delphi method was utilized to gain a consensus of experts based on their “collective intelligence” [3]. Participants were tasked with creating a checklist that could be provided to professionals on college campuses.

**Results**

College AOD administrators and prevention researchers addressed the feasibility of doing research-based intervention. A negotiation was conducted regarding what was conceptually needed to implement the intervention. Necessary steps and resources were identified and a checklist was produced in 1.5 days. The checklist included the following steps: 1) conduct a needs assessment, 2) identify key stakeholders, 3) convene meeting, 4) select interventions, and 5) monitor and evaluate.

**Conclusions**

This rapid model of translation resulted in a scalable, step-by-step checklist. The concise instructions found on checklists on how to implement prevention approaches will likely increase use of evidence-based prevention measures on college campuses. Additionally, the development of this translational approach will have utility in other health-related fields.

**References**

1. Hingson RW, Zha W, Weitzman ER. Magnitude of and trends in alcohol-related mortality and morbidity among U.S. college students ages 18-24, 1998-2005. J Stud Alcohol Drugs Suppl. 2009;16:12–20.

2. Nelson TF, Toomey TL, Lenk KM, Erickson DJ, Winters KC. Implementation of NIAAA College Drinking Task Force recommendations: How are colleges doing 6 years later? ACER. 2010; 34(10):1687–1693.

3. Gordon TJ. The delphi method. Washington, DC: American Council for the United Nations University.

## A52 Factors implicated in successful implementation: evidence to inform improved implementation from high and low-income countries

### Melanie Barwick^1,2,3,4,5^, Raluca Barac ^1,2^, Stanley Zlotkin ^1,4,5,6,7^, Laila Salim^8^, Marnie Davidson^9^

#### ^1^Research Institute, Hospital for Sick Children, Toronto, Ontario M5G 1X8, Canada; ^2^Child and Youth Mental Health Research Unit, Psychiatry, Hospital for Sick Children, Toronto, Ontario M5G 1X8, Canada; ^3^Department of Psychiatry, University of Toronto, Toronto, Ontario M5T 1R8, Canada; ^4^Dalla Lana School of Public Health, University of Toronto, Toronto, Ontario M5G 1X8, Canada; ^5^SickKids Centre for Global Child Health, Hospital for Sick Children, Toronto, Ontario M5G 1X8, Canada; ^6^Department of Nutritional Sciences, University of Toronto, Toronto, Ontario M5G 1X8, Canada; ^7^Department of Paediatrics, University of Toronto, Toronto, Ontario M5G 1X8, Canada; ^8^Health and Nutrition, Save the Children Canada, Toronto, Ontario M2P 2A8, Canada; ^9^Maternal, Newborn and Child Health, CARE Canada, Ottawa, Ontario K2E 7X6, Canada

##### **Correspondence:** Melanie Barwick (melanie.barwick@sickkids.ca) – SickKids Centre for Global Child Health, Hospital for Sick Children, Toronto, Ontario M5G 1X8, Canada

**Background**

Effective implementation of evidence in practice requires knowledge about factors implicated in successful implementation, as outlined in the Consolidated Framework for Implementation Research [CFIR, 1]. Two original studies (i, ii) are contrasted with a published paper (iii), all having different contexts: implementation of (i) motivational interviewing (MI) in child mental health in Canada [high income context, HIC; 2]; (ii) exclusive breastfeeding (EBF) in Ethiopia and Mali [low income global health context, LIC; 3]; and (iii) a weight management program (MOVE) with male veterans in USA [HIC, 4].

**Materials and methods**

The CFIR was used to examine 37 constructs in relation to successful implementation as measured by (i) EBF rates; (ii) clinician MI fidelity; and (iii) MOVE program participation rates. In all studies, qualitative data were coded deductively for frequency and/or valence of CFIR constructs.

**Results**

Eleven constructs were associated with implementation success across the three contexts (adaptability and relative advantage of the intervention; practitioner knowledge/beliefs and self-efficacy; communications, compatibility, relative priority, goals/feedback, leadership engagement, access to knowledge/information in the inner setting; and reflecting and evaluation process).

**Conclusions**

This comparative analysis of CFIR constructs is unique and highlights those that are implicated in successful implementation of interventions. Knowing which CFIR constructs are universally associated with implementation success can inform implementation approach and mitigate barriers across contexts that vary in income, target population, and focus. This can also inform the development of quantitative measures to more precisely target implementation barriers, and provides external validity for implementation methods across contexts.

**References**

1. Damschroder LJ, Aron DC, Keith RE, Kirsh SR, Alexander JA, Lowery JC. Fostering implementation of health services research findings into practice: A consolidated framework for advancing implementation science. Implement Sci. 2009 Oct; 4(1):50.

2. Barwick M, Barac R, Kimber M, Akrong L, Johnson S. with the CIHR Emerging Team for Knowledge Translation in Child and Youth Mental Health. Evaluating evidence- informed implementation and the Consolidated Framework for Implementation Research: A multi-case study of motivational interviewing in child and youth mental health. In preparation.

3. Barwick M, Barac R, Zlotkin S. Evaluation of effective implementation of exclusive breastfeeding in Ethiopia and Mali using the Consolidated Framework for Implementation Research. Hospital for Sick Children, Canada; 2015. http://www.can-mnch.ca/wp-content/uploads/2015/05/EBF-Research-Report-FINAL-July-29-2015.pdf.

4. Damschroder LJ, Lowery JC. Evaluation of a large-scale weight management program using the consolidated framework for implementation research (CFIR). Implement Sci. 2013 May 10; 8(1):51.

## A53 Tracking implementation strategies prospectively: A practical approach

### Alicia C. Bunger^1^, Byron J. Powell^2^, Hillary A. Robertson^1^

#### ^1^College of Social Work, Ohio State University, Columbus, OH, 43210, USA; ^2^Department of Health Policy and Management, Gillings School of Global Public Health, University of North Carolina, Chapel Hill, NC, 27599, USA

##### **Correspondence:** Alicia C. Bunger (bunger.5@osu.edu) – College of Social Work, Ohio State University, Columbus, OH, 43210, USA

**Background**

Published descriptions of implementation strategies often lack precision and consistency, limiting replicability and slowing accumulation of knowledge. Recent publication guidelines for implementation strategies call for improved description of the activities, dose, rationale, and expected outcome(s) of strategies [1]. However, capturing implementation strategies with this level of detail can be challenging, as responsibility for implementation is often diffuse and strategies may be flexibly applied as barriers and challenges emerge. We describe a practical approach to tracking implementation, and illustrate its use for describing strategies used over time and estimating time invested in implementation.

**Materials and methods**

This approach was piloted in an evaluation of a multi-component intervention to improve children’s access to behavioral health services in a county-based child welfare agency. Key project personnel completed a monthly activity log for 14 months. Logs collected information about implementation activities, intent, duration, and individuals involved. Using a consensus approach, two coders categorized each activity based upon Powell et al.’s taxonomy of implementation strategies [2].

**Results**

Participants reported on 420 activities, which represent 38 unique strategies, and account for 652 hours. Quality management strategies (e.g. developing monitoring tools and systems; 38 %), planning (32 %), and education (24 %) strategies were most frequently reported. Prior to intervention launch, implementation focused on planning and education, and accounted for 10-40 hours of effort per month. Post-launch, implementation focused on quality monitoring and accounted for 90-160 hours per month.

**Conclusions**

This prospective approach allows for implementation monitoring over time, estimating “dose,” and describing temporal ordering of implementation strategies.

**References**

1. Proctor EK, Powell BJ, McMillen JC. Implementation strategies: Recommendations for specifying and reporting. Implement Sci. 2013; 8(1):139.

2. Powell BJ, McMillen JC, Proctor EK, Carpenter CR, Griffey RT, Bunger AC, Glass JE, York JL. A compilation of strategies for implementing clinical innovations in health and mental health. Med Care Res Rev. 2012 Apr 1; 69(2):123-157.

## A54 Trained but not implementing: the need for effective implementation planning tools

### Christopher Botsko (christopher.botsko@altarum.org)

#### Altarum Institute, Ann Arbor, MI, 20910, USA

**Background**

A common occurrence when implementing evidence-based practices is that a large number of trained providers do not implement the practice [1]. This study explores the phenomena of failure to implement through an evaluation of the Triple P parenting support program in two communities.

**Materials and methods**

Data assessing progress on implementation was collected through surveys of advisory group members and providers, three years of annual interviews with key informants, and focus groups with parents and providers. Interview and focus group data were analyzed using NVivo.

**Results**

The data showed that the majority of trained practitioners did not implement the program because they were unable to integrate it into their existing services. Project leadership were provided with the newly developed Triple P Implementation Framework and they indicated that it gave them a better understanding of what implementation entailed, but they objected to what they perceived as the overly theoretical nature of the framework and indicated a need for more practical tools and information to effectively use the framework.

**Conclusions**

Using the findings of this study, a simple two-page implementation planning tool was developed that asks implementing organizations to describe who is going to be served by the practice, what staff are going to implement it, how staff are going to integrate the practice into their existing job, the source of short-term funding, and a plan for long-term funding. The tool is intended to stimulate discussion and planning around key implementation issues in a way that responds to the practical needs of practioners.

**Reference**

1. Fixsen DL, Naoom SF, Blase KA, Friedman RM. Implementation research: A synthesis of the literature. 2005.

## A55 Evidence, context, and facilitation variables related to implementation of Dialectical Behavior Therapy: Qualitative results from a mixed methods inquiry in the Department of Veterans Affairs

### Sara J. Landes^1,2^, Brandy N. Smith^1^, Allison L. Rodriguez^1^, Lindsay R. Trent^1^, Monica M. Matthieu^3,4^

#### ^1^National Center for Posttraumatic Stress Disorder (PTSD), Veterans Affairs (VA) Palo Alto Health Care System, Menlo Park, CA, 94025, USA; ^2^Department of Psychiatry, University of Arkansas for Medical Sciences, Little Rock, AR, 72205, USA; ^3^School of Social Work, Saint Louis University, Saint Louis, MO, 63103, USA; ^4^Mental Health Services, Central Arkansas Health Care System, North Little Rock, AR, 72114, USA

##### **Correspondence:** Sara J. Landes (sjlandes@uams.edu) – Department of Psychiatry, University of Arkansas for Medical Sciences, Little Rock, AR, 72205, USA

**Background**

Dialectical Behavior Therapy (DBT) [1] is an evidence-based psychotherapy designed to address suicidal behavior and emotion dysregulation. DBT is effective among female veterans with borderline personality disorder [2] and helpful in reducing Department of Veterans Affairs (VA) healthcare costs [3]. DBT has been implemented locally across VA but little is known about the system as a whole or how it has been implemented.

**Materials and methods**

Using the PARIHS model [4] as a conceptual framework, the study used sequential quantitative and qualitative methods [5, 6] to characterize DBT implementation. For a full description of methods, see Landes et al. [7]. Interviews were conducted with one clinician and one administrator at 16 sites. An a-priori code book was developed based on the PARIHS model and refined via consensus.

**Results**

Six administrator interview transcripts were included in the qualitative analyses. Evidence used to implement included reading the Linehan text [1], research support, and implicit knowledge. Contextual factors that facilitated implementation were leadership support, having an expert, culture, and being multi-disciplinary. Contextual factors that were barriers included lack of funding, training, leadership knowledge, and inclusion of DBT in VA policy. The following processes facilitated implementation: training, champions and opinion leaders, collaboration, technology, and making logistical changes.

**Conclusions**

Results confirm previous findings about barriers and facilitators to implementing evidence-based practices. Interviews offered examples of solutions that could be shared or inform policy changes. For example, logistical changes (e.g., cross clinic services, tiered system) could be included in implementation plans and policy suggestions to support implementation.

**Acknowledgments**

This study was funded by the Department of Veterans Affairs (VA), Mental Health Quality Enhancement Research Initiative (MH QUERI) QLP 55-055 awarded to the first author. The results described are based on data analyzed by the authors and do not represent the views of the VA, Veterans Health Administration (VHA), or the United States Government.

**References**

1. Linehan MM. Cognitive-behavioral treatment of borderline personality disorder. New York, NY: Guilford; 1993.

2. Koons CR, Robins CJ, Tweed JL, Lynch TR, Gonzalez AM, Morse JQ, Bishop GK, Butterfield MI, Bastian LA. Efficacy of dialectical behavior therapy in women veterans with borderline personality disorder. Behav Ther. 2001 Dec 31; 32(2):371–90.

3. Meyers LL, Landes SJ, Thuras P. Veterans’ service utilization and associated costs following participation in dialectical behavior therapy: A preliminary investigation. Mil Med. 2014;179:1368–73.

4. Kitson A, Harvey G, McCormack B. Enabling the implementation of evidence based practice: a conceptual framework. Qual Health Care. 1998;7:149–58.

5. Creswell J, Plano Clark VL. Designing and Conducting Mixed Methods Research. Aust N Z J Publ Heal. 2007;31:388–388.

6. Palinkas LA, Aarons GA, Horwitz S, Chamberlain P, Hurlburt M, Landsverk J. Mixed method designs in implementation research. Adm Policy Ment Hlth. 2011;38:44–53.

7. Landes SJ, Matthieu MM, Smith BN, Trent LR, Rodriguez AL, Kemp J, Thompson C. Dialectical behavior therapy training and desired resources for implementation: Results from a national program evaluation in the Veterans Health Administration. Mil Med. Forthcoming.

## A56 Learning from implementation as usual in children’s mental health

### Byron J. Powell^1^, Enola K. Proctor^2^

#### ^1^Department of Health Policy and Management, Gillings School of Global Public Health, University of North Carolina at Chapel Hill, Chapel Hill, NC 27599, USA; ^2^Brown School, Washington University in St. Louis, St. Louis, MO 63130, USA

##### **Correspondence:** Byron J. Powell (bjpowell@unc.edu) – Department of Health Policy and Management, Gillings School of Global Public Health, University of North Carolina at Chapel Hill, Chapel Hill, NC 27599, USA

**Background**

To ensure that implementation strategies are feasible, acceptable, sustainable, and scalable, efforts to identify and develop implementation strategies need to be grounded by a thorough understanding of real-world service systems and what constitutes “implementation as usual.” The aim of this multiple case study [1] was to identify and characterize the strategies used in six children’s mental health organizations, and to evaluate the extent to which implementation as usual reflects best practices specified in the implementation literature.

**Materials and methods**

Semi-structured interviews and focus groups were conducted with organizational leaders (*N* = 27) and clinicians (*N* = 58) respectively. Interviews were recorded, transcribed verbatim, and analyzed using qualitative content analysis. Further methodological details are reported in the published protocol [1].

**Results**

Across organizations, provider-focused strategies (e.g., training, supervision) were dominant however, these strategies were not offered at the frequency and intensity required to implement EBTs effectively. Multiple areas of implementation were not often addressed, including process, client, organizational, financial, and policy levels. Several problematic trends related were identified, such as the inconsistent provision of training and supervision, monitoring fidelity in unhelpful ways, and failing to measure or appropriately utilize clinical outcome data.

**Conclusions**

By highlighting strengths and weaknesses of implementation as usual in children’s mental health, this study can inform the development of implementation strategies that will be practical and effective. It highlights a need to develop and test a wider range of strategies, particularly those that address the organizational context of service delivery, and to ensure that they are delivered with adequate fidelity.

**Reference**

1. Powell BJ, Proctor EK, Glisson CA, Kohl PL, Raghavan R, Brownson RC, Stoner BP, Carpenter CR, Palinkas LA: A mixed methods multiple case study of implementation as usual in children’s social service organizations: Study protocol. Implement Sci. 2013;8:1–12.

## A57 Rates and predictors of implementation after Dialectical Behavior Therapy Intensive Training

### Melanie S. Harned^1,2^, Marivi Navarro-Haro^1^, Kathryn E. Korslund^1^, Tianying Chen^1^, Anthony DuBose ^2^, André Ivanoff^2^, Marsha M. Linehan^1^

#### ^1^Department of Psychology, University of Washington, Seattle, WA, 98195, USA; ^2^Behavioral Tech, LLC, Seattle, WA, 98105, USA

##### **Correspondence:** Melanie S. Harned (mharned@uw.edu) – Behavioral Tech, LLC, Seattle, WA, 98105, USA

**Background**

Dialectical Behavior Therapy (DBT) Intensive Training is the gold standard for training clinicians to deliver DBT. This team-based training includes two five-day workshops (Part 1 and Part 2) separated by a six-month period for self-study and implementation. Although DBT Intensive Training has been widely used, little research has evaluated its effectiveness. The present study evaluates the rates and predictors of implementation of DBT after DBT Intensive Training.

**Materials and methods**

Participants attended one of nine DBT Intensive Trainings (*N* = 411 clinicians from 81 teams) conducted from 2012-2013. All attendees completed self-report measures at the Part 1 and Part 2 workshops assessing characteristics of the clinician (demographics, education and training background, attitudes, self-efficacy, burnout), team (size, team needs), and organization (barriers to implementation, readiness to change). In addition, team leaders completed a follow-up survey 6-12 months (*M* = 8.7, *SD* = 3.5) after Part 2 to assess implementation.

**Results**

Overall, 75 % of teams had implemented all four DBT modes after training. Only 2 % of teams had not implemented any DBT mode. Predictor analyses were conducted using generalized linear models with the number of DBT modes implemented as a count outcome. Teams with fewer training and program needs at Part 2, a smaller proportion of bachelor’s-level clinicians, and clinicians with more prior experience delivering DBT implemented significantly more DBT modes.

**Conclusions**

These findings provide evidence of the effectiveness of DBT Intensive Training in promoting implementation of DBT among clinicians from diverse practice settings.

## A58 Socio-contextual determinants of research evidence use in public-youth systems of care

### Antonio R. Garcia^1^, Minseop Kim^2^, Lawrence A. Palinkas^3^, Lonnie Snowden^4^, John Landsverk^5^

#### ^1^School of Social Policy and Practice, University of Pennsylvania, Philadelphia, PA, 19104, USA; ^2^Department of Social Work, Chinese University of Hong Kong, Hong Kong, China; ^3^School of Social Work, University of Southern California, Los Angeles, CA, 90089, USA; ^4^School of Public Health, University of California-Berkeley, Oakland, CA, 94610, USA; ^5^School of Social Work, Washington University, St. Louis, MO, 63105, USA

##### **Correspondence:** Antonio R. Garcia (antgar@sp2.upenn.edu) – School of Social Policy and Practice, University of Pennsylvania, Philadelphia, PA, 19104, USA

**Background**

While evidence-based practices (EBPs) exist to promote positive outcomes among at-risk youth, they are not implemented to fidelity [1]. This may, in part, stem from inability of leaders to use research evidence [2]. The implementation of a randomized clinical trial comparing utilization of community development teams versus individual implementation of Multidimensional Treatment Foster Care provided an opportunity to examine Aarons et al. [3] conceptual underpinnings of implementation drivers. The main objective of this study, however, was to identify whether similar socio-contextual drivers of implementation predict research evidence use (REU).

**Materials and methods**

Socio-contextual drivers for 37 counties in California were gathered from public records in 2008; and public youth system leaders’ (*N* = 96) perceptions of REU were measured via the Structured Interview of Evidence Use (SIEU) between 2008 and 2012. The 45-item SIEU [4] asks respondents to indicate the extent they obtain (input), assess validity (process), and use (output) research evidence. Regressions were conducted to examine relationships between contextual determinants and the input, process, output, and total scores.

**Results**

On average, leaders reported a SIEU score of 3.37 (*SD* = .33) on a five-point scale. Higher educational attainment increased the likelihood of REU. Positive relationships between scores on the “input” subscale and racial minority concentration and poverty were detected.

**Conclusions**

Findings suggest leaders gather evidence to work effectively within poor and minority communities, but may decide to not rely on the evidence. Findings highlight the need to understand these relationships and hire leaders who are trained to use evidence.

**References**

1. Axford N, Morpeth L. Evidence-based programs in children's services: A critical appraisal. Child Youth Serv Rev. 2013 Feb 28;35(2):268-77.

2. Nutley SM, Walter I, Davies HT. Using evidence: How research can inform public services. Policy press; 2007.

3. Aarons GA, Hurlburt M, Horwitz SM. Advancing a conceptual model of evidence-based practice implementation in public service sectors. Adm Policy Ment Health. 2011 Jan 1;38(1):4-23.

4. Palinkas LA, Garcia A, Aarons GA, Holloway I, Finno M, Fuentes D, Chamberlain P. Measurement of implementation process: The structured interview of evidence use (SIEU) and cultural exchange inventory (CEI). Paper presented at: 5th Annual NIH Conference on the Science of Dissemination and Implementation; 2012 Washington, DC.

## A59 Community resource mapping to integrate evidence-based depression treatment in primary care in Brazil: A pilot project

### Annika C. Sweetland^1^, Maria Jose Fernandes^2^, Edilson Santos^2^, Cristiane Duarte^1^, Afrânio Kritski^3^, Noa Krawczyk^1^, Caitlin Nelligan^1^, Milton L. Wainberg^1^

#### ^1^Department of Psychiatry, Columbia College of Physicians and Surgeons and New York State Psychiatric Institute, New York, NY, 10032, USA; ^2^Itaboraí Municipality of Health, Itaboraí, Brazil; ^3^Federal University of Rio de Janeiro, Rio de Janeiro, Brazil

##### **Correspondence:** Annika C. Sweetland (acs2124@columbia.edu) – Department of Psychiatry, Columbia College of Physicians and Surgeons and New York State Psychiatric Institute, New York, NY, 10032, USA

**Background**

This pilot study used Global Positioning System (GPS) enabled smartphones to create a map of mental health resources and other relevant infrastructure within the public sector in Itaboraí, Brazil. These preliminary data and the community map will be used in planning for a dissemination and implementation study to integrate evidence-based depression treatment in primary care using tuberculosis (TB) as a model.

**Materials and methods**

Face-to-face interviews were conducted in all public health facilities in Itaboraí. Data were collected over a ten-week period using the open-source application Open Data Kit (ODK) Collect and uploaded to the Ona.io web platform. The survey included questions on mental health services, specialized staff, and resources, as well as procedures and protocols for the management of mental health disorders, particularly among individuals undergoing treatment for TB. Other basic information included the type and size of facility, location, services, staffing, accessibility, and infrastructure.

**Results**

Itaboraí has 50 public health facilities, of which 40 are community-based primary care clinics, and five are specialized mental health clinics. Of the 46 mental health professionals (psychiatrists, psychologists, or psychiatric nurses) in the public health system, only one was based in a primary care facility. Only two primary care clinics offered mental health services beyond referral. Among all facilities, 72 % had reliable access to running water, 30 % had consistent access to a computer, and 12 % had reliable Internet access. No facilities had a consistently functioning landline phone.

**Conclusions**

Community resource mapping using mobile phones is an efficient and valuable strategy for data visualization and planning for implementation and dissemination research.

## A60 The use of concept mapping to efficiently identify determinants of implementation in the National Institute of Health--President’s Emergent Plan for AIDS Relief Prevention of Mother to Child HIV Transmission Implementation Science Alliance

### Gregory A. Aarons^1^, David H. Sommerfeld^1^, Benjamin Chi^2^, Echezona Ezeanolue^3^, Rachel Sturke^4^, Lydia Kline^4^, Laura Guay^5^, George Siberry^6^

#### ^1^Department of Psychiatry, University of California, San Diego, La Jolla, CA, 92093, USA; ^2^Department of Obstetrics and Gynecology, University of North Carolina at Chapel Hill, Chapel Hill, NC, 27599, USA; ^3^School of Community Health Science, University of Nevada Las Vegas, Las Vegas, NV, 89154, USA; ^4^NIH Fogarty International Center, Bethesda, MD, 20892, USA; ^5^Elizabeth Glaser Pediatric AIDS Foundation, Washington, DC, 20036, USA; ^6^National Institute of Health (NIH) National Institute of Child Health and Human Development, Bethesda, MD, 20892, USA

##### **Correspondence:** Gregory A. Aarons (gaarons@ucsd.edu) – Department of Psychiatry, University of California, San Diego, La Jolla, CA, 92093, USA

**Background**

Human Immunodeficiency virus (HIV) acquisition for children in sub-Saharan Africa occurs primarily from mother-to-child transmission during pregnancy, childbirth, or breastfeeding. There is increasing interest in effective implementation of prevention of mother to child HIV transmission (PMTCT) [1,2]. The NICHD in collaboration with the National Institue of Health (NIH) Fogarty International Center and President’s Emergency Plan for AIDS Relief (PEPFAR), established the PMTCT Implementation Science Alliance (ISA) that supports and serves as a platform for NIH R01 implementation science grantees along with program implementers and policy-makers. Studies took place in Kenya, Mozambique, Nigeria, Zambia, South Africa, and the Democratic Republic of Congo. ISA members have a multi-dimensional vantage point to identify key implementation factors for PMTCT interventions across countries, communities, and cultures.

**Materials and methods**

We utilized Concept Mapping (CM), a mixed qualitative/quantitative method, over a two-week period, to distill implementation issues across projects and stakeholders [3]. ISA members responded to the focus question: “In your experience, what factors have facilitated or hindered implementation of PMTCT interventions?” Over 150 responses from ISA members (*N* = 50) online or in-person were distilled to 88 distinct statements. ISA members (*N* = 28) sorted statements into categories based on similarity and sort matrices were analyzed using multidimensional scaling and hierarchical cluster analysis.

**Results**

Key factors that influenced PMTCT implementation were identified (logistical/support services, clinic/provider services, personnel capacity, training/support, leadership-practice intersection, health system resources, tracking/monitoring, data measurement/collection, funding, evidence-based practice guidelines, governmental commitment, maternal-child clinical care, socio-cultural issues, local context, and community engagement).

**Conclusions**

CM can be efficiently utilized for understanding issues for multiple implementation strategies across stakeholders, cultures, countries, and health systems.

## A61 Longitudinal remote consultation for implementing collaborative care for depression

### Ian M. Bennett^1,2^, Rinad Beidas^3^, Rachel Gold^4^, Johnny Mao^1^, Diane Powers^1^, Mindy Vredevoogd^1^, Jürgen Unützer^1^

#### ^1^Department of Psychiatry and Behavioral Sciences, University of Washington, Seattle, WA, 98195, USA; ^2^Department of Family Medicine, University of Washington, Seattle, WA, 98195, USA; ^3^Department of Psychiatry, University of Pennsylvania, Philadelphia, PA, 19104, USA; ^4^Center for Health Research, Kaiser Permanente, Portland, OR, 97227, USA

##### **Correspondence:** Ian M. Bennett (jbennett@uw.edu) – Department of Family Medicine, University of Washington, Seattle, WA, 98195, USA

**Background**

A major obstacle to achieving the benefits to patients observed in effectiveness trials of complex interventions in large-scale implementation efforts is the limit of resources available to support the training to mastery of staff carrying out the intervention. Although ongoing support in the form of training, technical assistance, quality improvement, and tools improves both implementation and patient outcomes through longitudinal consultation by content experts, most large implementation efforts rely primarily on brief intensive training for staff because of cost limitations [1,2,3].

**Materials and methods**

We conceptualize consultants as intervention-specific practice facilitators within the Interactive Systems Framework [3,4]. We have developed an innovative and pragmatic remote model of longitudinal consultation for implementation of the team based collaborative care intervention for treatment of adult depression in primary care. Targeting key elements of the intervention we make use of video conferencing technologies to allow consult liaison psychiatrists to deliver this consultation to many sites simultaneously in an efficient manner.

**Results**

This strategy has been piloted in a multi-site implementation effort to assess acceptability. There is a high level of satisfaction with the approach by the implementation teams and reports of faster time to mastery of case reviews, which are a central component of this intervention.

**Conclusions**

Formal evaluation of this strategy is needed to assess its ability to support implementation of collaborative care in settings remote from the intervention specific practice facilitators.

**References**

1. Nadeem E, Gleacher A, Beidas RS. Consultation as an implementation strategy for evidence-based practices across multiple contexts: Unpacking the black box. Adm Policy Ment Health*.* 2013; 40(6):439- 450.

2. Edmunds JM, Beidas RS, Kendall PC. Dissemination and implementation of evidence-based practices: Training and consultation as implementation strategies. Clin Psychol-Sci Pr*.* 2013; 20(2):152-165.

3. Wandersman A, Chien VH, Katz J. Toward an evidence-based system for innovation support for implementing innovations with quality: Tools, training, technical assistance, and quality assurance/quality improvement. Am J Commun Psychol*.* 2012; 50(3-4):445-459.

4. Wandersman A, Duffy J, Flaspohler P, Noonan R, Lubell K, Stillman L, Blackman M, Dunville R, Saul J. Bridging the gap between prevention research and practice: The interactive systems framework for dissemination and implementation. Am J Commun Psychol*.* 2008; 41(3-4):171-181.

## A62 Integrating a peer coach model to support program implementation and ensure long- term sustainability of the Incredible Years in community-based settings

### Jennifer Schroeder^1^, Lane Volpe^1^, Julie Steffen^2^

#### ^1^The Implementation Group, Louisville, CO, US, 80027; ^2^Invest in Kids, Denver, CO, USA, 80203

##### **Correspondence:** Jennifer Schroeder (jen@theimplementationgroup.com) – The Implementation Group, Louisville, CO, US, 80027

**Background**

The Incredible Years (IY) is an evidence-based, social-emotional, skill-building program implemented in school- and community-based settings. As a community partner, Invest in Kids (IIK) serves as the Intermediary Purveyor in Colorado and provides support functions required for effective implementation, including readiness assessment, and ongoing training and coaching necessary to ensure sustainable replication of evidence-based programs.

**Materials and methods**

Since 2011, 30 teachers with at least two years of experience implementing the program have participated in 10 days of peer coach training. Each participant completed satisfaction and readiness surveys after each day of training so that IIK could support skill development in preparation for implementation of the Peer Coach model.

**Results**

During the 2012-2013 school year, peer coaches provided on-site coaching to their fellow teachers and themselves received ongoing supervision and coaching to ensure consistent delivery of the peer coach model. Peer coaches increased their self-reported understanding of the core components of fidelity to the model and how to best support teachers implementing the model from the beginning of their peer coach training to the beginning of their first year as a peer coach. This understanding increased further from their first to second year serving as a peer coach.

**Conclusions**

Peer coaching has been identified by IIK as an essential strategy for fostering community readiness, site-level sustainability, and ensuring long-term quality implementation. These results also highlight the need to support peer coaches over multiple years to improve their skills in understanding core fidelity and how to better support teachers implementing the model.

## A63 Efficient sustainability: Existing community based supervisors as evidence-based treatment supports

### Shannon Dorsey^1^, Michael D Pullmann^2^, Suzanne E. U. Kerns^2^, Nathaniel Jungbluth^1^, Lucy Berliner^3^, Kelly Thompson^1^, Eliza Segell^1^

#### ^1^Department of Psychology, University of Washington, Seattle, WA, 98195, USA; ^2^Psychiatry and Behavioral Sciences, University of Washington, Seattle, WA, 98102, USA; ^3^Harborview Center for Sexual Assault and Traumatic Stress, Harborview, Seattle, WA, 98104, USA

##### **Correspondence:** Shannon Dorsey (dorsey2@uw.edu) – Department of Psychology, University of Washington, Seattle, WA, 98195, USA

**Background**

Existing community-based supervisors (CBS) are an underutilized resource for supporting evidence-based treatments (EBTs) in community mental health, despite being a potentially efficient and affordable mechanism for EBT support.

**Materials and methods**

Data come from cross-sectional, self-report surveys from 56 supervisors and 209 of their clinicians across the state of Washington, all of whom were trained in a trauma-focused EBT for youth. Participants answered questions about individual and organizational characteristics and supervision experiences.

**Results**

71.8 % of the clinicians reported receiving weekly individual supervision. Over 70 % reported weekly/every other week group supervision, with frequent informal supervision. Supervisors and clinicians reported a high concordance of time spent on a variety of supervision functions in individual supervision, with the majority of the time allocated to clinical functions. Two clinical functions that we perceived as most EBT-relevant—case conceptualization and interventions— comprised about one-third of the supervision hour. Variance in how much time was spent on these EBT-relevant functions clustered more substantially at the supervisor level than for overall clinical functions (32 % vs. 20 %) (EBT ICC = .318; -2 L-D χ^2^ = 18.3, *p* < .001; AIC-D χ^2^ = 16.3, *p* < .001). Positive implementation climate was associated with more time spent on these functions. Notably, both supervisors (98.2 %) and clinicians (89.5 %) overwhelmingly nominated case conceptualization and treatment intervention as functions to which more time should be allocated.

**Conclusions**

These findings suggest that CBS are using a variety of supervision modalities to support clinicians and that they need to be included in implementation efforts.

**Acknowledgements**

NIMH-funded; MH095749 (Dorsey, PI)

## A64 Establishment of a national practice-based implementation network to accelerate adoption of evidence-based and best practices

### Pearl McGee-Vincent^1^, Nancy Liu^1^, Robyn Walser^1,2^, Jennifer Runnals^3,4^, R. Keith Shaw^3^, Sara J. Landes^1,5^, Craig Rosen^1,6^, Janet Schmidt^1^, Patrick Calhoun^4^

#### ^1^National Center for Posttraumatic Stress Disorder (PTSD), Veterans Affairs (VA) Palo Alto Health Care System, Menlo Park, CA, 94025, USA; ^2^Department of Psychology, University of California Berkeley, Berkeley, CA, 94720, USA; ^3^VA Mid-Atlantic Mental Illness Research Educational and Clinical Center (MIRECC), Durham VA Medical Center, Durham, NC, 27705, USA; ^4^Department of Psychiatry and Behavioral Sciences, Duke University School of Medicine, Durham, NC, 27710, USA; ^5^Department of Psychiatry, University of Arkansas for Medical Sciences, Little Rock, AR, 72205, USA; ^6^Department of Psychiatry and Behavioral Sciences, Stanford University School of Medicine, Stanford, CA, 94305, USA

##### **Correspondence:** Sara J. Landes (sara.landes@va.gov) – Department of Psychiatry, University of Arkansas for Medical Sciences, Little Rock, AR, 72205, USA

**Background**

Practice-based research networks are groups of providers and researchers working together to examine health care processes in broad populations of patients and settings to improve outcomes [1]. We adapted this model and developed a practice-based implementation network in the United States Department of Veterans Affairs (VA) and Department of Defense to facilitate adoption and implementation of mental health best practices.

**Materials and methods**

The network utilized two implementation strategies, evidence-based quality improvement paired with external facilitation (EBQI/EF) and technical assistance (TA) [2], to increase routine outcomes monitoring in post-traumatic stress disorder (PTSD) treatment. Program evaluation included quantitative self-report surveys of providers to assess use of the PTSD Checklist (PCL) at baseline, repeated use, and discussion with patients.

**Results**

Eighteen VA clinics (134 providers) across three clinic types (specialty PTSD [*N* = 11], general mental health [*N* = 5], primary care clinics [*N* = 2]) participated in the network. The first 10 sites received EBQI/EF. When additional sites requested participation, they were added to the network and offered TA (*N* = 8). Clinician-reported repeated administration of the PCL increased by about 50 % in EBQI/EF sites, and use of PCL data in decision-making increased by 50 % in EBQI/EF sites and 30 % in TA sites. Discussion of PCL data with patients did not increase.

**Conclusions**

Creation of the network was feasible and both implementation strategies were feasible and appeared to have an impact. The inclusion of different clinic types and sites with different levels of implementation was ideal for this network strategy, as it allowed sites to learn from each other and get support.

**Acknowledgments**

This project was funded by the Department of Veterans Affairs (VA) and Department of Defense (DoD) Joint Incentive Fund (JIF). The results described are based on data analyzed by the authors and do not represent the views of the VA, Veterans Health Administration (VHA), or the United States Government.

**References**

1. Lindbloom EJ, Ewigman BG, Hickner JM. Practice-based research networks: The laboratories of primary care research. Med Care. 2004; 42:III-45-III-49.

2. Powell BJ, Waltz TJ, Chinman MJ, Damschroder LJ, Smith JL, Matthieu MM, Proctor EK, Kirchner JE. A refined compilation of implementation strategies: results from the Expert Recommendations for Implementing Change (ERIC) project. Imp Sci. 2015; 10:21.

## A65 Facilitation as a mechanism of implementation in a practice-based implementation network: Improving care in a Department of Veterans Affairs Posttraumatic Stress Disorder outpatient clinic

### Ruth L. Varkovitzky^1,2^, Sara J. Landes^3,4^

#### ^1^Veterans Affairs (VA) Puget Sound Health Care System – American Lake Division, Tacoma, WA, 98493, USA; ^2^Department of Psychiatry and Behavioral Science, University of Washington School of Medicine, Seattle, WA, 98105, USA; ^3^National Center for Posttraumatic Stress Disorder (PTSD), VA Palo Alto Health Care System, Menlo Park, CA, 94025, USA; ^4^Department of Psychiatry, University of Arkansas for Medical Sciences, Little Rock, AR, 72205, USA

##### **Correspondence:** Ruth L. Varkovitzky (ruth.varkovitzky@va.gov) – Department of Psychiatry and Behavioral Science, University of Washington School of Medicine, Seattle, WA, 98105, USA

**Background**

Facilitation is an implementation strategy that bundles discrete implementation interventions and focuses on partnering with clinical and administrative personnel at the site implementing a practice change. Facilitation was utilized in the Practice-Based Implementation Network created by the United States. Department of Veteran Affairs (VA) and Department of Defense [1]. The goal of the first network project was to increase routine outcomes monitoring. Results from one clinic are presented.

**Materials and methods**

Eight mental health providers in a VA posttraumatic stress disorder (PTSD) outpatient clinic aimed to increase use of the PTSD Checklist for Diagnostic and Statistical Manual-5 [PCL-5; DSM-5; 2] for treatment planning and program development. A champion managed the project locally and liasoned with a facilitator. Qualitative data on the experience of the clinic and champion were collected in problem-solving meetings with the facilitator.

**Results**

Challenges included: clinician concern about time to participate, inefficient methods for completion of measures, problems with the electronic medical record integrating scores to allow tracking of PCL use in the clinic, and difficulty easily using the PCL to make treatment decisions. Solutions included: champion lessened burden of data collection and made participation reinforcing, patient self-service kiosk, clinic dataset, and data graphing function.

The team was recognized by hospital leadership for their efforts with the project.

**Conclusions**

Lessons learned included: the need for clarifying system-level and team-level goals, the importance of reinforcing project participants, and highlighting the value of programmatic data. After completion of this project, two additional clinicians requested to participate, an additional assessment tool was added, and the project is now clinic-wide.

**Acknowledgments**

The results described are based on data analyzed by the authors and do not represent the views of the VA, Veterans Health Administration (VHA), or the United States Government.

**References**

1. McGee-Vincent P, Liu N, Walser R, Runnals, Shaw JR, Landes SJ, Rosen C, Schmidt J, Calhoun P, Establishment of a national practice-based implementation network to accelerate adoption of evidence-based and best practices. A64.

2. Weathers, FW, Litz, BT, Keane, TM, Palmieri, PA, Marx, BP, Schnur, PP. The PTSD Checklist for DSM-5 (PCL-5). Scale available from the National Center for PTSD at www.ptsd.va.gov.

## A66 The ACT SMART Toolkit: An implementation strategy for community-based organizations providing services to children with autism spectrum disorder

### Amy Drahota^1,2*^, Jonathan I. Martinez^3^, Brigitte Brikho^1,2^, Rosemary Meza^4^, Aubyn C. Stahmer^2,5^, Gregory A. Aarons^2,6^

#### ^1^Department of Psychology, San Diego State University, San Diego, CA, 92182, USA; ^2^Child and Adolescent Service Research Center, San Diego, CA, 92123, USA; ^3^Department of Psychology, California State University Northridge, Northridge, CA, 91330, USA; ^4^Department of Psychology, University of Washington, Seattle, WA, 98195, USA; ^5^Department of Psychiatry and Behavioral Sciences, University of California Davis, Medical Investigation of Neurodevelopmental Disorders (MIND) Institute, Davis, CA, 95616, USA; ^6^Department of Psychiatry, University of California San Diego, San Diego, CA, 92093, USA

##### **Correspondence:** Amy Drahota – Child and Adolescent Service Research Center, San Diego, CA, 92123, USA

**Background**

One in 68 children are diagnosed with autism (ASD), costing $268 billion for services annually [1,2]. While evidence-based practices (EBPs) are available for use with individuals with ASD [3,4], utilization of EBPs in for-profit community-based organizations (ASD-CBO) varies considerably. Evaluating implementation-as-usual practices and factors influencing EBP implementation may help the development of setting-specific implementation tools.

**Materials and methods**

ASD-CBO agency leaders (AL, *N* = 20) and direct providers (DP, *N* = 27) completed the Autism Model of Implementation Survey. Ten AL completed an additional qualitative interview. Data (Quan→→QUAL) were converged for triangulation within and across methods.

**Results**

**Implementation-as-Usual Practices***.* Needs Evaluation: Few ASD-CBO (31 %) reported evaluating service and delivery gaps to guide EBP identification and implementation, and this process is informal. EBP Identification and Adoption: AL commonly learned of EBPs through conferences (25 %) and literature (63 %); DP reported learning of EBP through literature (32 %) or were uncertain (26 %). EBP adoption was not linked to agency need. AL reported adoption decisions involve gathering staff opinions (25 %), DP were not sure how adoption decisions were made (37 %). Implementation Strategies: AL and DP reported utilizing few implementation strategies: piloting and revising (AL:19 %, DP:11 %), staff training only (AL:25 %, DP:0 %), and “just using [EBP]” (AL:31 %, DP:21 %). **Factors Influencing Implementation.** Client need/progress, EBP evidence, EBP adaptability/flexibility, EBP feasibility, funding, EBP fit with climate/culture, staff training requirements, and staff expertise.

**Conclusions**

A comprehensive implementation strategy, the ACT SMART Toolkit©, supports EBP implementation by systematically identifying service delivery gaps; matching EBPs to ASD- CBO needs; facilitating adoption decisions; planning for implementation; and evaluating EBP implementation efforts.

**References**

1. Centers for Disease Control and Prevention. Prevalence of autism spectrum disorder among children aged 8 years – Autism and Developmental Disabilities Monitoring Network, 11 Sites, United Stated, 2010. MMWR Surveill Summ. 2014;63(SS02);1-21.

2. Leigh JP, Du J. Brief report: Forecasting the economic burden of autism in 2015 and 2025 in the United States. J Autism Dev Disord. 2015;45:4135-4139.

3. National Autism Center. Findings and conclusions: National standards project, phase 2. Randolph, MA: National Autism Center; 2015.

4. Wong C, Odom SL, Hume KA, Cox AW, Fettig A, Kucharczyk S, Brock ME, Plavnick JB, Fleury VP, Schultz TR. Evidence-based practices for children, youth, and young adults with autism spectrum disorder: A comprehensive review. J Autism Dev Disord. 2015 Jul 1; 45(7):1951-66.

## A67 Supporting Policy In Health with Research: An intervention trial (SPIRIT) - protocol and early findings

### Anna Williamson (anna.williamson@saxinstitute.org.au)

#### The Sax Institute, Sydney, NSW, 2000, Australia

**Background**

Although many programs have been designed to assist policy agencies to better use research in their work, there have been few tests of the effectiveness of such programs. This paper describes the protocol and early findings from Supporting Policy In Health with Research: an Intervention Trial (SPIRIT).

**Materials and methods**

SPIRIT was a stepped wedge cluster randomized trial set in Sydney, Australia involving six health policy agencies [1]. It was designed to test the effectiveness of a highly tailored, multifaceted program to build organizational capacity for the use of research evidence in policy development and was based on the SPIRIT Action Framework [2]. The primary aim was to determine whether SPIRIT increased the extent to which participating agencies accessed, appraised, generated and used research in the development of policies. The current paper draws on quantitative and qualitative data from the baseline measures and from the SPIRIT process evaluation.

**Results**

Early findings from SPIRIT suggested that participating agencies valued research evidence. Although the trial was intensive, agencies were actively engaged. At time one, agencies differed in relation to staff confidence regarding research use and in the agency-wide systems and structures that were in place to support it. Nevertheless, some common themes emerged in relation to areas in which capacity building was sought, with the majority of agencies requesting sessions on evaluation, critical appraisal and social media messaging.

**Conclusions**

The SPIRIT intervention was feasible to implement and agencies were highly engaged in the process.

**Acknowledgements**

This paper was presented on behalf of the CIPHER Investigators. The authors wish to thank the people and organizations participating in the CIPHER project.

**References**

1. Investigators C, others: Supporting Policy In health with Research: an Intervention Trial (SPIRIT)—protocol for a stepped wedge trial. BMJ open 2014, 4.

2. Redman S, Turner T, Davies H, Williamson A, Haynes A, Brennan S, Milat A, O'Connor D, Blyth F, Jorm L, Green S. The SPIRIT Action Framework: A structured approach to selecting and testing strategies to increase the use of research in policy. Soc Sci Med. 2015 Jul 31; 136:147-55.

## A68 From evidence based practice initiatives to infrastructure: Lessons learned from a public behavioral health system’s efforts to promote evidence based practices

### Ronnie M. Rubin^1^, Byron J. Powell^2^, Matthew O. Hurford^3^, Shawna L. Weaver^1^, Rinad S. Beidas^4^, David S. Mandell^4^, Arthur C. Evans^1^

#### ^1^Department of Behavioral Health and Intellectual disAbilities Services, Philadelphia, PA, 19107, USA; ^2^University of North Carolina, Chapel Hill, NC, 27599, USA; ^3^Community Care Behavioral Health Organization, Pittsburgh, 15222, USA; ^4^University of Pennsylvania, Philadelphia, PA, 19104, USA

##### **Correspondence:** Ronnie M. Rubin (ronnie.rubin@phila.gov) – Department of Behavioral Health and Intellectual disAbilities Services, Philadelphia, PA, 19107, USA

**Background**

Over the past decade, implementation science has shifted from a focus on therapist knowledge, skills, and behavior to include contextual and system factors that influence implementation and clinical outcomes. The Philadelphia Department of Behavioral Health and Intellectual disAbilities Services (DBHIDS) has undergone a similar shift in how evidence based practices (EBPs) are promoted from a system-level perspective.

**Materials and methods**

DBHIDS supported several EBP initiatives, including Cognitive Behavioral Therapy (CBT), Prolonged Exposure, Trauma-focused CBT and Dialectical Behavior Therapy. These initiatives included training, consultation, and implementation support to 62 agencies. Implementing multiple EBPs simultaneously provided a unique vantage point to formulate lessons learned about community EBP implementation. Input was gathered from key partners and system leaders throughout the course of these efforts. System-level challenges and opportunities were identified.

**Results**

Limitations of an initiatives-based approached to EBP implementation included generating a “catalog” of EBPs without an overarching strategy, and challenges with sustainability, scale, and measuring impact in a large system. Strategies to address these limitations included taking a broader approach, 1) from training therapists to engaging organizations, 2) from a focus on the development of practice skill to including the development of clinical and operational support for EBPs within agencies, and 3) from approaching EBPs as “add-ons” to integrating them within agencies and system operations.

**Conclusions**

Training initiatives provide important opportunities to begin to develop EBP capacity within agencies and to identify system-level challenges, but long-term impact of EBPs requires strategies that integrate EBPs into the clinical, organizational and operational infrastructure of the system.

## A69 Applying the policy ecology model to Philadelphia’s behavioral health transformation efforts

### Byron J. Powell^1^, Rinad S. Beidas^2^, Ronnie M. Rubin^3^, Rebecca E. Stewart^2^, Courtney Benjamin Wolk^2^, Samantha L. Matlin^4^, Shawna Weaver^3^, Matthew O. Hurford^5^, Arthur C. Evans^3^, Trevor R. Hadley^2^, David S. Mandell^2^

#### ^1^Department of Health Policy and Management, Gillings School of Global Public Health, University of North Carolina at Chapel Hill, Chapel Hill, NC, 27599, USA; ^2^Center for Mental Health Policy and Services Research, Department of Psychiatry, Perelman School of Medicine, University of Pennsylvania, Philadelphia, PA, 19104, USA; ^3^Department of Behavioral Health and Intellectual disAbility Services, Philadelphia, PA, 19107, USA; ^4^Scattergood Foundation, Philadelphia, PA, 19102, USA; ^5^Community Care Behavioral Health Organization, Philadelphia, PA, 19107, USA

##### **Correspondence:** Byron J. Powell (bjpowell@unc.edu) – Department of Health Policy and Management, Gillings School of Global Public Health, University of North Carolina at Chapel Hill, Chapel Hill, NC, 27599, USA

**Background**

Implementation frameworks emphasize the importance of support at the intervention, individual, team, organizational, and broader system levels [1, 2] however, most implementation strategies focus narrowly on educating and supporting clinicians [3, 4]. Raghavan et al. [5] argued the importance of addressing the ‘policy ecology,’ which includes organizations, regulatory and purchasing agencies, political entities, and broader social forces. The present study applied the policy ecology model [5] to characterize the Philadelphia Department of Behavioral Health and Intellectual disAbility Services’ (DBHIDS) efforts to implement evidence-based practices (EBPs).

**Materials and methods**

Published reports, meeting notes, and informal interviews with DBHIDS leadership were used to document implementation strategies that have been used in Philadelphia’s large-scale implementation efforts. These strategies were then matched to the four levels of Raghavan et al.’s [5] policy ecology framework to illustrate how multi-faceted, multi-level strategies can be aligned to facilitate the implementation of EBPs.

**Results**

DBHIDS has used strategies to address implementation barriers at each level of the policy ecology, including the organizational- (e.g., paying for clinicians’ lost billable hours, building organizational capacity), funding or regulatory agency- (e.g., ensuring care managers are well informed about EBPs), political- (e.g., engaging stakeholders to build buy-in), and social-levels (e.g., sponsoring public events that address stigma and raise awareness of EBPs).

**Conclusions**

This study contributes to the emerging literature on system-level implementation strategies, demonstrates how they can be used to promote the integration of effective practices, and broadens the scope of activities typically described or empirically tested in the implementation literature.

**References**

1. Damschroder LJ, Aron DC, Keith RE, Kirsh SR, Alexander JA, Lowery JC. Fostering implementation of health services research findings into practice: A consolidated framework for advancing implementation science. Implement Sci 2009;4:1–15.

2. Aarons GA, Hurlburt M, Horwitz SM. Advancing a conceptual model of evidence-based practice implementation in public service sectors. Adm Policy Ment Health Ment Health Serv Res. 2011;38:4–23.

3. Powell BJ, Proctor EK, Glass JE. A systematic review of strategies for implementing empirically supported mental health interventions. Res Soc Work Pract. 2014;24:192– 212.

4. Novins DK, Green AE, Legha RK, Aarons GA. Dissemination and implementation of evidence-based practices for child and adolescent mental health: A systematic review. J Am Acad Child Adolesc Psychiatry. 2013;52:1009–1025.

5. Raghavan R, Bright CL, Shadoin AL. Toward a policy ecology of implementation of evidence-based practices in public mental health settings. Implement Sci. 2008;3:1–9.

## A70 A model for providing methodological expertise to advance dissemination and implementation of health discoveries in Clinical and Translational Science Award institutions

### Donald R. Gerke, Beth Prusaczyk, Ana Baumann, Ericka M. Lewis, Enola K. Proctor

#### Brown School of Social Work, Washington University in St. Louis, St. Louis, MO, 63130, USA

##### **Correspondence:** Donald R. Gerke (dgerke@wustl.edu) – Brown School of Social Work, Washington University in St. Louis, St. Louis, MO, 63130, USA

**Background**

Institutions supported by Clinical and Translational Science Awards (CTSAs) are tasked with advancing translational science [1]. The Dissemination and Implementation Research Core (DIRC) at Washington University’s CTSA supports investigators by providing methodological expertise to advance scientific agenda and grant writing towards dissemination and implementation (D&I) of health discoveries.

**Materials and methods**

Strategies employed by DIRC include: providing consultation to investigators during one-on-one appointments and weekly walk-in clinic; creating “toolkits” for each area of D&I to assist DIRC members during consultations and provide investigators with tools to strengthen their own capacity to conduct D&I research; working with a strong team comprising masters and doctoral- level research assistants and faculty, each with a focused area of expertise in D&I (e.g. outcomes and measurement, design, strategies, etc.). DIRC team building activities include semi-monthly meetings for quality assurance, and to provide mentoring and peer support of each members’ own work in D&I research.

**Results**

Since its inception in 2011, the number of DIRC customers has steadily increased. In 2011, 19 investigators sought DIRC resources, followed by 29 in 2012 and 30 in 2013. Although there was a slight decrease in 2014 (*N* = 24), as of September 2015, DIRC had assisted more customers in 2015 than in any other year (*N* = 32).

**Conclusions**

DIRC may serve as a model for other CTSAs supporting investigators in the development of translational research proposals.

**Reference**

1. Clinical and Translational Science award programs fact sheet [Internet]. Bethesda: National Center for Advancing Translational Sciences; 2015 [cited 2016 Feb 08]. Available from: https://ncats.nih.gov/files/CTSA-factsheet.pdf

## A71 Establishing a research agenda for the Triple P Implementation Framework

### Jenna McWilliam^1^, Jacquie Brown^1^, Michelle Tucker^2^

#### ^1^Triple P International, Brisbane, Queensland, Australia; ^2^Queensland University of Technology, Brisbane, Queensland, Australia

##### **Correspondence:** Jenna McWilliam (jenna@triplep.net) – Triple P International, Brisbane, Queensland, Australia

**Background**

The Triple P Implementation Framework (the Framework) was developed by Triple P International, the purveyor organisation for the Triple P–Positive Parenting Program® (Triple P). The Framework supports communities to develop the capacity for effective, sustainable implementation and is a package of strategies, methods, and techniques to enhance the adoption, implementation, and sustainability of Triple P. These two studies are the first step towards establishing a research agenda to evaluate the effectiveness of the Framework.

**Materials and methods**

The first study explored reports from practitioners of how informed and prepared they felt at training during Group Triple P courses between 2012-2015 (*N* = 15,562) and of the appropriateness of Triple P for their work (*N* = 15,590). The second study explored implementation experiences from a sample of these practitioners within three years of training. The relationship between the training experience, organizational practices, perceptions of implementation climate, and use of Triple P was examined (*N* = 161). Both studies used qualitative self-report data.

**Results**

Study 1 found that since the introduction of the Framework practitioners reported that Triple P is more appropriate for their work (*t*(15561) = 16.77, *p* < .0001) and that they were more informed and prepared ahead of training (*t*(15589) = 7.51, *p* < .0001). Study 2 found a significant correlation (*r*_*s*_ = .188, *p =* .017) between the number of families a practitioner had delivered the program to and their mean score on the measure of Implementation Climate.

**Conclusions**

This study provides preliminary findings for the impact of the Framework. Preliminary analyses are in the expected directions and further research is proposed to evaluate the effectiveness of the Framework.

**Acknowledgements**

The Triple P – Positive Parenting Program® (Triple P) is owned by The University of Queensland. The University, through its technology transfer company, UniQuest Pty Ltd., has licensed Triple P International Pty Ltd to publish and disseminate the program worldwide. Royalties stemming from published Triple P resources are distributed to the Parenting and Family Support Centre, School of Psychology, Faculty of Health and Behavioural Sciences, and

contributory authors. The development of the Triple P Implementation Framework was funded by Triple P International Pty Ltd.

**Completing Interests**

No author has any share or ownership in Triple P International Pty Ltd. Jenna McWilliam is an employee of Triple P International. Jacquie Brown is a consultant with Triple P International.

## A72 Cheap and fast, but what is “best?”: Examining implementation outcomes across sites in a state-wide scaled-up evidence-based walking program, Walk With Ease

### Kathleen P Conte (kathleen.conte@sydney.edu.au)

#### Menzies Centre for Health Policy, University of Sydney, New South Wales, Sydney, 2004, Australia

**Background**

Scaling-up programs through established delivery systems can accelerate dissemination and reduce costs [1], however, research guiding best-practices for scaling-up and evaluating outcomes is lacking [2]. This mixed-method study examines outcomes of a two-year state-wide scale-up of a simple, evidence-based walking program in relationship to cost, speed, and effectiveness of implementation.

**Materials and methods**

To facilitate implementation and share resources, multi-sector community partnerships were established. Partners contributed volunteer/staff time to delivery and received free training and program materials. Participant outcomes (*N* = 598) were assessed via registration/satisfaction forms; scale-up outcomes were assessed via interviews with leaders (*N* = 39), administrative reports and observations.

**Results**

In-person leader trainings (versus online) accelerated leader recruitment and initiation. Classes implemented by staff [OR = 3.1, p < .05] and senior centers [OR = 3.0, p < .05] best retained program participants. Interviews indicated implementation was enhanced in sites whose leaders demonstrated a clear understanding of program goals and saw the program as good fit.

Participants reported significant reduction in pain and fatigue (β = -0.47, p < .01, β = -0.58, p < .05), and increased physical activity (β = 0.86, p < .001). Most programs were delivered with high fidelity, however, adaptations and participant retention posed threats to the quality of program delivery.

**Conclusions**

Despite limited funding, scale-up goals were met in terms of participant enrollment, and program effectiveness was evidenced. Maximizing partnerships contributed to fast and cheap wide-scale implementation. By engaging program leaders in personal interaction via in-person trainings, scope, speed, and quality of implementation were improved. Our findings, however, evidence potential threats to quality delivery and highlight the need for ongoing monitoring and evaluation of scale-up efforts.

**Acknowledgements**

This research was conducted at Oregon State University and supported in part by a grant from the John Erkkila Foundation at the Good Samaritan Foundation.

**References**

1. Brady TJ, Sniezek J, Ramsey LA. News from the CDC: Scaling up sustainable intervention delivery–lessons learned from the CDC arthritis program. Transl Behav Med. 2012; 2(1), pp.3-5.

2. Milat A, Bauman, Redman S. Narrative review of models and success factors for scaling up public health interventions*.* Implement Sci. 2015;10(1):113.

## A73 Measurement feedback systems in mental health: Initial review of capabilities and characteristics

### Aaron R. Lyon^1^, Meredith R. Boyd^2^, Abigail Melvin^2^, Cara C. Lewis^2^, Freda Liu^1^, Nathaniel Jungbluth^1^

#### ^1^University of Washington, Seattle, WA, 98115, USA; ^2^ Indiana University, Bloomington, IN, 47405 USA

##### **Correspondence:** Aaron R. Lyon (lyona@uw.edu)

**Background**

Measurement Feedback Systems (MFS) are emerging Health Information Technologies (HIT) that provide feedback to clinicians about client progress and outcomes, allowing for data-driven clinical decision-making. Moreover, HIT like MFS provide avenues for efficient methodological approaches to data collection in the context of implementation. Despite the existence of many MFS and strong evidence of benefits of their use, information about systems and their functionality is fragmented, limiting uptake and utility. It is also unclear the extent to which MFS design has incorporated relevant theories and frameworks, such as Feedback Intervention Theory [1].

**Materials and methods**

This project sought to identify every MFS available and document their specific features. Forty- nine MFS were identified via systematic literature and internet searches and coded for 56 capabilities and characteristics (e.g., tracks interventions delivered by providers; provides standard-gap feedback) informed by relevant literature and feedback from experts and community stakeholders.

**Results**

Our review of systems suggests incredible variability in MFS. For example, ten systems are highly customizable, allowing for the addition of new tools and measures. The remaining 39 offer a set library of measures that cannot be altered.

**Conclusions**

Our findings emphasize the critical need for MFS information consolidation and comparison. Little is currently known about which MFS capabilities are most facilitative of evidence-based practice implementation. The current project provides researchers and stakeholders with rich information supporting efficient MFS selection. Results are discussed with respect to system capabilities, alignment with FIT, and the use of MFS as an efficient methodology for supporting implementation-related data collection. Subsequent project phases will evaluate system implementability and spread.

**References**

1. Kluger AN, DeNisi A. The effects of feedback interventions on performance: A historical review, a meta-analysis, and a preliminary feedback intervention theory. Psychol Bull. 1996;119:254–284.

## A74 A qualitative investigation of case managers’ attitudes toward implementation of a measurement feedback system in a public mental health system for youth

### Amelia Kotte^1^, Kaitlin A. Hill^1^, Albert C. Mah^1^, Priya A. Korathu-Larson^1^, Janelle R. Au^2^, Sonia Izmirian^1^, Scott Keir^3^, Brad J. Nakamura^1^, Charmaine K. Higa-McMillan^2^

#### ^1^University of Hawaii at Manoa, Honolulu, HI, 96822, USA; ^2^University of Hawaii at Hilo, Hilo, HI, 96720, USA; ^3^Child and Adolescent Mental Health Division (CAMHD), Honolulu, HI, 96720, USA

##### **Correspondence:** Charmaine K. Higa-McMillan (higac@hawaii.edu) – University of Hawaii at Hilo, Hilo, HI, 96720, USA

**Background**

Hawai’i’s Child and Adolescent Mental Health Division (CAMHD) initiated a state-wide quality improvement effort for administering the Ohio Scales (OS) on a monthly basis as part of a measurement feedback system (MFS) initiative.

**Materials and methods**

Surveys are collected by system case managers (CM). Reports are generated monthly to longitudinally track youth outcomes and increase client-level data-driven decision-making. This qualitative study seeks to understand barriers and facilitators associated with implementing an MFS. CMs (*N =* 61) received training on OS administration and MFS interpretation prior to implementation. Interviews were conducted three months after training and implementation with the thirty-nine CMs who consented to the interview. After transcription, 25 interviews were coded by seven trained coders using an open inductive approach and an external auditor.

**Results**

Central themes related to facilitators of implementation included perceptions that the OS adds to clinical decision-making and facilitates good practices. Most common barriers were: reports that the OS/MFS was not routinely utilized in supervision and utilization management meetings, and that administration of the OS is too time consuming.

**Conclusions**

In order to help with sustainment efforts, a number of system inner context features must be developed. These efforts should leverage facilitators and address barriers of implementation, such as building clearly defined workflows, providing ongoing support for CMs, and instituting fidelity monitoring strategies.

## A75 Multiple pathways to sustainability: Using Qualitative Comparative Analysis to uncover the necessary and sufficient conditions for successful community-based implementation

### Brittany Rhoades Cooper, Angie Funaiole, Eleanor Dizon

#### Department of Human Development, Washington State University, Pullman, WA, 99164, USA

##### **Correspondence:** Brittany Rhoades Cooper (brittany.cooper@wsu.edu) – Department of Human Development, Washington State University, Pullman, WA, 99164, USA

**Background**

A clear understanding of the conditions necessary and sufficient for successful sustainability is critically important to realizing the public health impact of programs. Existing research conceptualizes sustainability as a static binary endpoint and few studies account for the multifaceted nature of program sustainability.

**Materials and methods**

This mixed-method study explores the multiple factors associated with sustainability of Strengthening Families Program (SFP; an evidence-based, family-focused, youth substance use prevention program) sites across WA State. Facilitators (*N* = 59) completed the Program Sustainability Assessment Tool (PSAT) [1] and reported their sustainability level. Twenty also participated in semi-structured interviews. Qualitative Comparative Analysis (QCA), was used to identify sets of conditions necessary, sufficient, or both to attain sustainability success.

**Results**

Bivariate correlations showed that all but one PSAT scale were positively related with sustainability, including environmental support, funding stability, partnerships, organizational capacity, program evaluation, communication, and strategic planning. QCA analyses revealed that having a supportive internal and external climate for the program (environmental support), in combination with strong internal support and resources needed to effectively manage the program (organizational capacity), were conditions consistently present in those sites that achieved high levels of sustainability. These themes were validated by the interviews.

**Conclusions**

QCA offers a more comprehensive picture of which combinations of factors promote successful sustainability. For SFP, it appears that the combination of both environmental support and organizational capacity are key ingredients.

**Reference**

1. Luke DA, Calhoun A, Robichaux, CB, Elliott, MB, Moreland-Russell, S. The Program Sustainability Assessment Tool: A new instrument for public health programs. Prev Chronic Dis. 2014;11:E12.

## A76 Prescribers’ perspectives on opioids and benzodiazepines and medication alerts to reduce co-prescribing of these medications

### Eric J. Hawkins^1,2,4,5^, Carol A. Malte^1,2^, Hildi J. Hagedorn^3,4^, Douglas Berger^6,8^, Anissa Frank^1,2^, Aline Lott^1,2^, Carol E. Achtmeyer^1^, Anthony J. Mariano^5,7^, Andrew J. Saxon^1,2^

#### ^1^Health Services Research & Development (HSR&D) Seattle Center of Innovation for Veteran- Centered and Value-Driven Care, Seattle, WA, USA; ^2^Center of Excellence in Substance Abuse Treatment and Education, Veterans Affairs (VA) Puget Sound Health Care System, Seattle, WA, USA; ^3^Minneapolis VA Health Care System, Minneapolis, VA, USA; ^4^VA Quality Enhancement Research Initiative for Substance Use Disorders, Palo Alto, CA, USA; ^5^Department of Psychiatry and Behavioral Sciences, University of Washington, Seattle, WA, 98195, USA; ^6^Primary and Specialty Medical Care Service, VA Puget Sound Health Care System, Seattle, WA, 98108, USA; ^7^Veteran Integrated Service Network 20 Pain Medicine and Functional Rehabilitation Center, VA Puget Sound Health Care System, Seattle, WA, 98108, USA; ^8^Department of Medicine, University of Washington, Seattle, WA, 98195, USA

##### **Correspondence:** Eric J. Hawkins (eric.hawkins@va.gov) – Department of Psychiatry and Behavioral Sciences, University of Washington, Seattle, WA, 98195, USA

**Background**

Due to trends in pharmaceutical overdoses involving opioids and benzodiazepines, reducing the co-prescribing of these medications is a national priority [1], particularly among patients with high-risk conditions (e.g., substance use disorders). Medication alerts have been identified as interventions with potential to improve patient safety [2]. However, little is known about prescribers’ perspectives on these medications and use of alerts to reduce co-prescribing.

**Materials and methods**

The Promoting Action on Research Implementation in Health Services framework [3] guided survey and semi-structured interview development. Prescribers of opioids or benzodiazepines at one multisite Veterans Affairs (VA) healthcare system were invited to participate in an anonymous survey to assess perspectives on opioid and benzodiazepine co-prescribing among Veterans with high-risk conditions (*N* = 186; response rate = 47.3 %). Qualitative interviews were conducted with a subset of prescribers (*N* = 26) with exposures to the alert to identify facilitators and barriers to modifying prescribing practices and using the alert.

**Results**

Most prescribers agreed with clinical guidelines that discourage opioid and benzodiazepine co- prescribing to patients with high-risk conditions. However, barriers to discontinuing co- prescribing included insufficient time, unpleasant interactions with patients reluctant to discontinue the medication(s) and frustration with responsibility of tapering patients inherited from other prescribers. Factors supporting use of the alert included that it was easy to use and identified patients at high risk, while barriers included alert fatigue and additional time required to process the alert.

**Conclusions**

Prescribers reported several barriers that contribute to opioid and benzodiazepine co-prescribing and challenge their ability to discontinue these medications. While the alert was well accepted, multiple interventions likely are needed to reduce opioid and benzodiazepine co-prescribing.

**References**

1. Kitson AL, Rycroft-Malone J, Harvey G, McCormack B, Seers K, Titchen A. Evaluating the successful implementation of evidence into practice using the PARiHS framework: Theoretical and practical challenges. Implement Sci*.* 2008;3:1.

2. Wolfstadt JI, Gurwitz JH, Field TS, Lee M, Kalker S, Wu W, Rochon PA. The effect of computerized physician order entry with clinical decision support on the rates of adverse drug events: A systematic review. J Gen Intern Med*.* Apr 2008; 23(4):451–458.

3. Park TW, Saitz R, Ganoczy D, Ilgen MA, Bohnert AS. Benzodiazepine prescribing patterns and deaths from drug overdose among US veterans receiving opioid analgesics: Case-cohort study*.* Brit Med J*.* 2015; 350.

## A77 Adaptation of Coordinated Anxiety Learning and Management for comorbid anxiety and substance use disorders: Delivery of evidence-based treatment for anxiety in addictions treatment centers

### Kate Wolitzky-Taylor^1^, Richard Rawson,^1^ Richard Ries,^2^ Peter Roy-Byrne,^2^ Michelle Craske^3^

#### ^1^Department of Psychiatry and Biobehavioral Sciences, University of California-Los Angeles, Los Angeles, CA, USA; ^2^Department of Psychiatry, University of Washington, Seattle, WA, USA; ^3^Department of Psychology, University of California-Los Angeles, Los Angeles, CA, USA

##### **Correspondence:** Kate Wolitzky-Taylor (kbtaylor@mednet.ucla.edu) – University of California-Los Angeles, Department of Psychiatry and Biobehavioral Sciences, Los Angeles, CA, USA

**Background**

Most treatment-seeking individuals with anxiety disorder and substance use disorder (SUD) comorbidity are treated for SUD in specialty clinics but do not receive treatment for their anxiety disorders, which are associated with poor SUD treatment outcomes. This study developed and evaluated an adaptation of a computerized, therapist-directed CBT program for anxiety disorders (CALM) to increase access to EBTs for anxiety in this comorbid population.

**Materials and methods**

In this effectiveness/implementation hybrid study, the CALM program was adapted to to be suitable for delivery in addictions treatment centers. After training addictions treatment counselors to deliver the treatment, we conducted a randomized clinical trial comparing usual care at the addiction clinic (UC) to UC + the CALM adaptation (CALM for Addiction Recovery Centers; CALM ARC). Currently, 49 patients at the community clinic with comorbid anxiety disorders and SUD have been randomized. Preliminary outcomes (measured at baseline, post- treatment, and 6-mo follow-up) included measures of feasibility, acceptability, and anxiety and substance use symptom outcomes.

**Results**

Therapists demonstrated competency in delivering CALM ARC. At post-treatment, CALM ARC outperformed UC on treatment satisfaction, quality of life, anxiety reduction, depression reduction, drinking in the past 30 days, and drug use in the past 30 days. CALM ARC patients had greater percentages of negative urinalysis compared to those in UC (66 % v. 45 % in UC).

**Conclusions**

Findings thus far suggest delivery of CBT for anxiety in addictions counseling centers is feasible, acceptable, and effective. We await additional data to draw conclusions about our follow-up assessment.

## A78 Opportunities and challenges of measuring program implementation with online surveys

### Dena Simmons, Catalina Torrente, Lori Nathanson, Grace Carroll

#### Yale University, Department of Psychology, New Haven, CT, 06511 USA

##### **Correspondence:** Dena Simmons (dena.simmons@yale.edu) – Yale University, Department of Psychology, New Haven, CT, 06511 USA

**Background**

This study used online surveys to explore whether individual characteristics of school-based trainers predicted successful implementation of RULER, a K-8th school-based approach to social and emotional learning. School-based trainers (e.g., counselors, teachers, school leaders) attended a four-day RULER train-the-trainer institute in 2013 or 2014 as the starting point for implementing RULER. We hypothesized implementation would be positively associated with individual self-efficacy and favorable attitudes about the program, and negatively with perception of challenges.

**Materials and methods**

Data were collected during and six months post-training, including: demographic and professional characteristics, self-efficacy, attitudes, knowledge about the program, implementation activities, and challenges. We fitted multilevel models to account for nesting of individuals within schools.

**Results**

Forty-six participants (51 % response rate) completed the surveys and reported implementing five out of ten RULER activities on average. Self-efficacy (*b* = 7.48, *p* < .005; ES = 2.67) and attitudes about the program (*b* = 2.46, p < .005; ES = .88) were positively associated with implementation, whereas perception of challenges had a negative association (*b* = -4.37, *p* < .005; ES = 1.56).

Other characteristics (e.g., other trainings, experience) had smaller impacts.

**Conclusions**

Forty-six participants (51 % response rate) implemented half of the RULER activities, and there were significant positive associations between RULER implementation and personal characteristics such as self-efficacy and positive attitudes about the program. As such, when train-the-trainer models prioritize activities that strengthen participant’s self-efficacy and positive attitudes about the program, higher levels of implementation are likely. In addition, trainings that proactively address common implementation challenges (e.g., lack of time, difficulties aligning the program with other initiatives) may yield stronger implementation.

## A79 Observational assessment of fidelity to a family-centered prevention program: Effectiveness and efficiency

### Justin D. Smith^1^, Kimbree Brown^2^, Karina Ramos^3^, Nicole Thornton^4^, Thomas J. Dishion^4,5^, Elizabeth A. Stormshak^6^, Daniel S. Shaw^7^, Melvin N. Wilson^8^

#### ^1^Department of Psychiatry and Behavioral Sciences, Feinberg School of Medicine, Northwestern University, Chicago, IL, 60657, USA; ^2^Oregon Social Learning Center, Eugene, OR, 97401, USA; ^3^University Health Services, University of California Berkeley, Berkeley, CA, 94720, USA; ^4^Oregon Research Institute, Eugene, OR, 97403, USA; ^5^REACH Institute, Department of Psychology, Arizona State University, Tempe, AZ, 85287, USA; ^6^Prevention Science Institute, University of Oregon, Eugene, OR, 97403, USA; ^7^Department of Psychology, University of Pittsburgh, Pittsburgh, PA, 15260, USA; ^8^Department of Psychology, University of Virginia, Charlottesville, VA, 22904, USA

##### **Correspondence:** Justin D. Smith (jd.smith@northwestern.edu) – Department of Psychiatry and Behavioral Sciences, Feinberg School of Medicine, Northwestern University, Chicago, IL, 60657, USA

**Background**

Accurate assessment of fidelity to evidence-based programs has critical implications for implementation at scale. A breakdown in fidelity inhibits the ability to draw valid inferences regarding the nature of a putative treatment effect. In this study, we conducted a randomized experiment to determine whether ratings of fidelity to the Family Check-Up (FCU) [1] provided on the COACH rating system [2] could differentiate levels of therapist training in the model.

**Materials and methods**

Trained coders observationally rated 75 videotaped sessions for fidelity using the COACH. We randomly selected 25 sessions each from the the intervention arm of an efficacy trial [4]; the intervention arm of an effectiveness trial [5]; and the control arm of the effectiveness trial where family-based services were provided by licensed therapists with no FCU training. A non- parametric test (Kruskal-Wallis) was used due to non-normality fidelity score distributions.

**Results**

Only one of the COACH dimensions (conceptually accurate in the FCU) was significantly different between the conditions after applying a Bonferroni correction (*χ*^2^ = 44.63, p < 0.00001). There was a trend for the dimension of Caregiver Engagement (*χ*^2^ = 13.47, p = 0.00119); these were in the expected direction.

**Conclusions**

These findings indicate that the COACH reliably differentiates fidelity level to the FCU on a critical dimension. The next step involves testing whether this dimension accounts for variability in outcomes, as previous studies have shown [5, 6].

**Acknowledgements**

National Institute on Drug Abuse (DA016110, DA027828), National Institute of Mental Health (MH020012), and Centers for Disease Control and Prevention (CE001389-01).

**References**

1. Dishion TJ, Stormshak EA. Intervening in children's lives: An ecological, family- centered approach to mental health care*.* Washington, DC: American Psychological Association; 2007.

2. Dishion TJ, Smith JD, Gill AM, Shaw DS, Knutson N. Family Check-Up & Everyday Parenting Fidelity COACH Rating Manual: Version 4.0. Phoenix, AZ: ASU REACH Institute, Arizona State University; 2014.

3. Dishion TJ, Shaw DS, Connell A, Gardner FEM, Weaver C, Wilson M. The Family Check-Up with high-risk indigent families: Preventing problem behavior by increasing parents’ positive behavior support in early childhood. Child Dev*.* 2008; 79(5):1395–1414.

4. Smith JD, Stormshak EA, Kavanagh K. Results of a pragmatic effectiveness- implementation hybrid trial of the Family Check–Up in community mental health agencies. Adm Policy Ment Hlth*.* 2015;42(3):265–278.

5. Smith JD, Dishion TJ, Shaw DS, Wilson MN. Indirect effects of fidelity to the Family Check-Up on changes in parenting and early childhood problem behaviors. J Consult Clin Psychol*.* 2013;81(6):962–974.

6. Chiapa A, Smith JD, Kim H, Dishion TJ, Shaw DS, Wilson MN. The trajectory of fidelity in a multiyear trial of the Family Check-Up predicts change in child problem behavior. J Consult Clin Psychol*.* 2015; 83(5):1006–1011.

## A80 Strategies and challenges in housing first fidelity: A multistate qualitative analysis

### Mimi Choy-Brown^1^, Emmy Tiderington^2^, Bikki Tran Smith^3^, Deborah K. Padgett^1^

#### ^1^Silver School of Social Work, New York University, New York, NY, 10003, USA; ^2^School of Social Work, Rutgers University, New Brunswick, NJ, 08901, USA; ^3^School of Social Service Administration, University of Chicago, Chicago, IL 60637, USA

##### **Correspondence:** Mimi Choy-Brown (mimi.choybrown@nyu.edu) – Silver School of Social Work, New York University, New York, NY, 10003, USA

**Background**

Pathways Housing First (PHF), an evidence-based model of permanent housing and supportive services for homeless adults, has been widely disseminated in the United States and internationally [1]. However, model fidelity has varied widely across settings [2]. Less understood are on-the-ground experiences of providers at different stages of implementation and organizational contextual factors influencing fidelity. Further knowledge of these provider experiences can inform more efficient and effective strategies to support fidelity in multiple contexts. The primary aim of this study was to understand the strategies and challenges providers encounter in the uptake and fidelity of PHF.

**Materials and methods**

Data were derived from a National Institute of Mental Health-funded study investigating perspectives of front-line staff and supervisors. Six focus groups were conducted with staff at PHF sites in three East Coast cities (*N* = 33). Thematic analysis [2] was utilized to code and develop themes regarding challenges to model fidelity.

**Results**

Funding context, turnover and logistics of home-based service provision challenged PHF fidelity. Strategies included the use of technology, supervision and team leadership, ongoing training, and community engagement. Challenges and strategies differentiated across fidelity criteria as providers adapted limited resources to respond to barriers to fidelity with some success.

**Conclusions**

Similar challenges to PHF fidelity have been identified across community mental health services. However, PHF may be particularly sensitive to outer-contextual factors of funding and housing requirements. PHF scale-up efforts would benefit from more in-depth knowledge of how technology, direct practice supervision and community engagement strategies contribute to successful PHF fidelity.

**References**

1. Tsemberis S, Gulcur L, Nakae M. Housing first, consumer choice, and harm reduction for homeless individuals with a dual diagnosis. Am J Public Health. 2004 Apr; 94(4):651-6.

2. Padgett DK, Henwood BF, Tsemberis SJ. Housing First: Ending homelessness, Transforming systems, and Changing Lives. New York: Oxford, 2016.

3. Boyatzis RE. Transforming Qualitative Information. Cleveland: Sage, 1998.

## A81 Procurement and contracting as an implementation strategy: Getting To Outcomes® contracting

### Ronnie M. Rubin^1^, Marilyn L. Ray^2^, Abraham Wandersman^3^, Andrea Lamont^3^, Gordon Hannah^4^, Kassandra A. Alia^3^, Matthew O. Hurford^5^, Arthur C. Evans^1^

#### ^1^Department of Behavioral Health and Intellectual disAbilities Services, Philadelphia, PA, 19107, USA; ^2^Finger Lakes Law and Social Policy Center, Ithaca, NY, 14850, USA; ^3^University of South Carolina, Columbia, SC, 29208, USA; ^4^Gordon Hannah Consulting, Pittsburgh, PA, 15201, USA; ^5^Community Care Behavioral Health Organization, Pittsburgh, 15222, USA

##### **Correspondence:** Ronnie M. Rubin (ronnie.rubin@phila.gov) – Department of Behavioral Health and Intellectual disAbilities Services, Philadelphia, PA, 19107, USA

**Background**

Billions of dollars are spent each year on behavioral health services and there is a movement to require the use of evidence based practices (EBPs) in these services. Financing, contracting and regulatory strategies are proposed in several implementation science frameworks; however, there are few systematic evaluations of how these strategies are deployed. Utilizing the Getting To Outcomes (GTO®) framework, the Philadelphia Department of Behavioral Health and Intellectual Disabilities Services (DBHIDS) engaged in an initiative to integrate EBPs and outcomes into the procurement and contracting functions of its public behavioral health managed care organization.

**Materials and methods**

In the Getting To Outcomes Contracting (GTOC) Initiative, GTO was used in the development of a Request for Proposals (RFP) for a behavioral health service. To evaluate the impact of GTOC, three independent experts rated the GTOC RFP and a comparable RFP on eight components of quality program planning. Interviews of key personnel were conducted for a qualitative evaluation of changes to procurement processes.

**Results**

The post-GTOC RFP had higher quality descriptions of program planning components (38.5 points out of 40) compared to the pre-GTOC RFP (11.5 points). Qualitative interviews identified strengths of GTOC initiative, including the structure and interdepartmental collaboration in RFP development, and clarification of needs and resources, goals and objectives, and outcomes of the service being procured. Challenges identified included, time commitment, need for role clarification and staff turnover.

**Conclusions**

This effort provides an example of how a behavioral health system can leverage quality implementation processes to procure evidence based services.

## **A82 Web-based feedback to aid successful implementation: The interactive Stages of Implementation Completion (SIC)**^TM^**tool**

### Lisa Saldana, Holle Schaper, Mark Campbell, Patricia Chamberlain

#### Oregon Social Learning Center, Eugene, Oregon 97401, USA

##### **Correspondence:** Lisa Saldana (lisas@oslc.org) – Oregon Social Learning Center, Eugene, Oregon 97401, USA

**Background**

The Stages of Implementation Completion (SIC) is an eight-staged measure that was developed to evaluate the implementation of evidence-based practices (EBPs). Each stage maps onto three phases of implementation: pre-implementation, implementation, and sustainability. The SIC measures adopting sites’ implementation performance, as indicated by activity completion and duration. Pre-implementation performance has been shown to predict successful implementation outcomes. The SIC has sound psychometrics.

**Materials and methods**

This project aims to provide efficient tools to increase the uptake of EBPs, thereby increasing the availability of services and decreasing wasted resources from failed implementation efforts.

Leveraging advances in technology and website development, the Interactive SIC was developed to allow for (a) a growing repository of SIC data to increase the sensitivity and accuracy of SIC prediction models, and (b) real-time, web-based feedback to be delivered by purveyors to sites to provide guidance toward successful implementation.

**Results**

In collaboration with a computer programming team, the Interactive SIC was designed and programmed. To date, 11 EBP groups have utilized the tool, reporting ease of use and interpretability. The purveyor enters an organization’s completion dates for implementation activities. Drawing from the data repository, the organization’s performance as compared to successful peer organizations is graphed and available to inform feedback regarding the pace and thoroughness of the implementation effort. The website functions on both Mac and PC platforms.

**Conclusions**

Real-time tools can help improve the odds of successful implementations by identifying areas in need of intervention (e.g., additional support/consultation) and strategies that best meet the needs of organizations.

## A83 Efficient methodologies for monitoring fidelity in routine implementation: Lessons from the Allentown Social Emotional Learning Initiative

### Valerie B. Shapiro^1^, B.K. Elizabeth Kim^1^, Jennifer L. Fleming^2^, Paul A. LeBuffe^2^

#### ^1^School of Social Welfare, University of California Berkeley, Berkeley, CA, 94618, USA; ^2^Devereux Center for Resilient Children, Devereux Foundation, Villanova, PA, 19085, USA

##### **Correspondence:** Valerie B. Shapiro (vshapiro@berkeley.edu) – School of Social Welfare, University of California Berkeley, Berkeley, CA, 94618, USA

**Background**

The Promoting Alternative Thinking Strategies (PATHS) curriculum is an effective school-based prevention program [1,2] that hinges upon quality implementation [3]. PATHS practice guidelines recommend technical assistance (TA) providers monitor 20 % of lessons but monitoring eight to ten lessons district-wide is challenging in routine practice. Research- informed guidance is unavailable to suggest how implementation quality should be efficiently monitored. This paper explores the relationship between various observation elements, observation completion rates, and overall implementation quality to improve monitoring efficiency.

**Materials and methods**

Two TAs attempted to observe 170 classrooms (15 schools) eight times. Observations included teacher characteristics (e.g., teacher is committed to implementation), adherence (e.g., teacher uses PATHS techniques), participant responsiveness (e.g., students enjoy PATHS activities), and Overall Implementation Quality (an independent point-in-time rating). Multilevel statistical modeling accounted for data clustering.

**Results**

Completion Rates: Observation completion rates ranged from 94 % (Time 1) to 32 % (Time 8). Implementation Quality was high and correlated across the school year among those observed. Initial Implementation Quality was unrelated to observation completion rates. Teachers’ initial commitment to high-level of implementation predicted completion rates.

Implementation Quality: Within each time point, teacher characteristics and participant responsiveness generally predicted Implementation Quality. Adherence, however, was only predictive for some lessons. Across time, teacher characteristics and participant responsiveness were stable, but adherence varied by lessons. Finally, initial Participant Responsiveness predicted end-of-year Sustained Implementation Quality ratings among those observed at Time 6-8.

**Conclusions**

Findings suggest fewer/briefer observations may be sufficient and that initial monitoring could suggest areas for targeted coaching to sustain observations and implementation quality.

**Acknowledgements**

This project was enabled through the support of the Pennsylvania Departments of Education, Health, and Public Welfare and the Hellman Foundation. The authors acknowledge Gerald L.

Zahorchak, C. Russell Mayo, Susan Lozada, Robin Powlus, John Monahan, Katherine Ross, Julie Koenigsberg, and Jennifer Croner who each played an essential role in the completion of this work.

**References**

1. Greenberg M, Kusche C, Cook E, Quamma J. Promoting emotional competence in school-aged children: The effects of the PATHS curriculum. Dev Psychopathol. 1995; 7:117-136.

2. Kam C, Greenberg M, Kusche C. Sustained effects of the PATHS curriculum on the social and psychological adjustment of children in special education. J Emot Behav Disord. 2004;12:66-78.

3. Kam C, Greenberg M, Walls C. Examining the role of implementation quality in school- based prevention using the PATHS curriculum. Prev Sci. 2003;4:55-63.

## A84 The Society for Implementation Research Collaboration (SIRC) implementation development workshop: Results from a new methodology for enhancing implementation science proposals

### Sara J. Landes^1,2^, Cara C. Lewis^3,4^, Allison L. Rodriguez^1^, Brigid R. Marriott^3^, Katherine Anne Comtois^4^

#### ^1^National Center for PTSD, VA Palo Alto Health Care System, Menlo Park, CA, 94025, USA; ^2^Department of Psychiatry, University of Arkansas for Medical Sciences, Little Rock, AR, 72205, USA; ^3^Department of Psychological and Brain Sciences, Indiana University, Bloomington, IN, 47405, USA; ^4^Department of Psychiatry and Behavioral Sciences, University of Washington School of Medicine, Seattle, WA, 98104, USA

##### **Correspondence:** Sara J. Landes (sjlandes@uams.edu) – Department of Psychiatry, University of Arkansas for Medical Sciences, Little Rock, AR, 72205, USA

**Background**

There is a dearth of training and technical assistance opportunities in the field of implementation science. The Society for Implementation Research Collaboration (SIRC) developed the Implementation Development Workshop (IDW) to provide critical and rich feedback that enhances the rigor and relevance of proposals in development. This highly structured and facilitated IDW is based on the Behavioral Research in Diabetes Group Exchange (BRIDGE) model [1] and was modified by SIRC to deliver it in two formats, face-to-face and virtual.

**Materials and methods**

A mixed method approach was used to evaluate the effectiveness and acceptability of the IDW and compare the two formats. IDW participants (*N* = 38) completed an anonymous quantitative survey assessing perceptions of the IDW. Presenters (*N* = 13) completed a funding survey to assess grant submission and funding success. Qualitative interviews were conducted with IDW participants who participated in both formats (*N* = 8).

**Results**

Face-to-face and virtual participants agreed they had a better understanding of implementation science principles and methods and thought they could apply what they learned. Of the presenters who completed the survey, 83 % submitted their proposal for funding and of those who submitted, 40 % received funding and 27 % plan to resubmit. There was a preference for the face-to-face format; however, both formats were deemed acceptable and satisfying. Qualitative interviews indicated that the structured process of the IDW appeared to impact acceptability (e.g., clear structure, facilitator, note taker).

**Conclusions**

Results indicated that participants found IDWs helpful and both formats were acceptable. SIRC will continue to host and evaluate IDWs in both formats.

**Acknowledgments**

The preparation of this presentation was supported, in kind, through the National Institutes of Health R13 award R13MH086159 entitled, “Development and Dissemination of Rigorous Methods for Training and Implementation of Evidence-Based Behavioral Health Treatments” granted to PI: KA Comtois from 2010-2015. The results described are based on data analyzed by the authors and do not represent the views of the Department of Veterans Affairs (VA), Veterans Health Administration (VHA), or the United States Government.

**Reference**

1. Behavioral Research in Diabetes Group Exchange - BRIDGE - Psychosocial Aspects of Diabetes (PSAD) Study Group [Internet]. [cited 2016 Feb 12]. Available from: http://uvtapp.uvt.nl/fsw/spits.ws.frmShowpage?v_page_id=9618214012013366

## A85 An update on the Society for Implementation Research Collaboration (SIRC) Instrument Review Project

### Cara C. Lewis^1,4^, Cameo Stanick^2^, Bryan J. Weiner^3^, Heather Halko^2^, Caitlin N. Dorsey^1^

#### ^1^Psychological and Brain Sciences, Indiana University, Bloomington, IN, 47405, USA; ^2^Department of Psychology, University of Montana, Missoula, MT 59812, USA; ^3^University of North Carolina at Chapel Hill, Chapel Hill, NC 27599, USA; ^4^Department of Psychiatry and Behavioral Sciences, 4899 University of Washington School of Medicine, Seattle, WA, 98104, USA

##### **Correspondence:** Cara C. Lewis (clewis11@uw.edu) – Department of Psychiatry and Behavioral Sciences, 4899 University of Washington School of Medicine, Seattle, WA, 98104, USA

**Background**

Significant gaps related to measurement issues are among the most critical barriers to advancing implementation science [1]. Notably, there is a lack of stakeholder involvement in defining pragmatic measure qualities and unknown psychometric and pragmatic strength of existing measures. The Society for Implementation Research Collaboration Instrument Review Project aims to address these gaps by first generating a stakeholder-driven operationalization of the pragmatic measures construct [2].

**Materials and methods**

The preliminary dimensions of the pragmatic construct were delineated via inductive and deductive methods. First, a systematic literature review was conducted. All synonyms of the ‘pragmatic’ construct (e.g., ‘usefulness’) and/or dimension terms/phrases (e.g., ‘ease of scoring’) were included. Second, interviews with seven stakeholder representatives from a variety of mental health settings (e.g., inpatient, outpatient, residential, school) were conducted and qualitatively coded. The results from both methods were combined to reveal preliminary pragmatic dimensions.

**Results**

The literature review revealed 32 unique domains/dimensions, whereas the interviews revealed 25 domains (e.g., cost) and 11 dimensions (e.g., less than $1.00 per measure) of the pragmatic construct, as well as 16 antonyms (e.g., costly). A final list of 47 items (both domains and dimensions) was retained after removing redundant and/or confusing items.

**Conclusions**

Results from the inductive and deductive methods revealed significantly more and diverse pragmatic measure qualities than those articulated in the recent literature [2]. The next phase of the project will clarify the internal structure of the pragmatic construct using concept mapping, followed by stakeholder prioritization using Delphi methodology.

**Acknowledgements**

Research reported in this publication was supported by the National Institute of Mental Health of the National Institutes of Health under Award Number R01MH106510. The preparation of this manuscript was also supported, in kind, through the National Institutes of Health R13 award entitled, “Development and Dissemination of Rigorous Methods for Training and Implementation of Evidence-Based Behavioral Health Treatments” granted to PI: KA Comtois from 2010–2015.

**References**

1. Martinez RG, Lewis CC, Weiner BJ. Instrumentation issues in implementation science. Implement Sci. 2014;9:118.

2. Lewis CC, Weiner BJ, Stanick C, Fisher SM. Advancing implementation science through measure development and evaluation: A study protocol. Implement Sci. 2015 Jul 22;10:102

3. Glasgow RE, Riley WT. Pragmatic measures: what they are and why we need them. Am J Prev Med. 2013;45:237–43.

